# Renewable Resources for Enantiodiscrimination: Chiral Solvating Agents for NMR Spectroscopy from Isomannide and Isosorbide

**DOI:** 10.1021/acs.joc.2c01244

**Published:** 2022-09-08

**Authors:** Federica Balzano, Anna Iuliano, Gloria Uccello-Barretta, Valerio Zullo

**Affiliations:** Dipartimento di Chimica e Chimica Industriale, Università di Pisa, Via Giuseppe Moruzzi, 13, 56124 Pisa, Italy

## Abstract

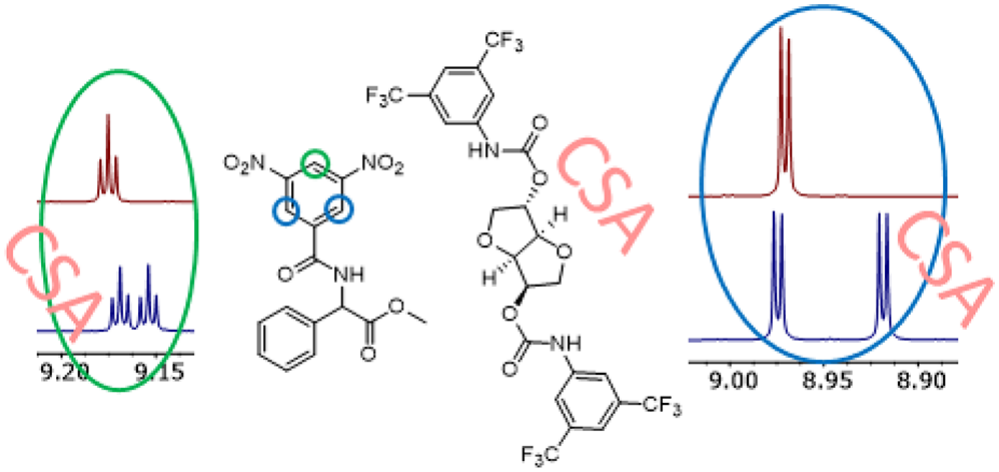

A new family of chiral selectors was synthesized in a single synthetic step with yields up to 84% starting from isomannide and isosorbide. Mono- or disubstituted carbamate derivatives were obtained by reacting the isohexides with electron-donating arylisocyanate (3,5-dimethylphenyl- or 3,5-dimethoxyphenyl-) and electron-withdrawing arylisocyanate (3,5-bis(trifluoromethyl)phenyl-) groups to test opposite electronic effects on enantiodifferentiation. Deeper chiral pockets and derivatives with more acidic protons were obtained by derivatization with 1-naphthylisocyanate and *p*-toluenesulfonylisocyanate, respectively. All compounds were tested as chiral solvating agents (CSAs) in ^1^H NMR experiments with *rac*-*N*-3,5-dinitrobenzoylphenylglycine methyl ester in order to determine the influence of different structural features on the enantiodiscrimination capabilities. Some selected compounds were tested with other racemic analytes, still leading to enantiodiscrimination. The enantiodiscrimination conditions were then optimized for the best CSA/analyte couple. Finally, a 2D- and 1D-NMR study was performed employing the best performing CSA with the two enantiomers of the selected analyte, aiming to determine the enantiodiscrimination mechanism, the stoichiometry of interaction, and the complexation constant.

## Introduction

Chirality plays a crucial role in medicinal, biological, and synthetic chemistry. Since most of the active pharmaceutical ingredients (APIs) are optically active molecules, there is a growing need for simple, fast, easy, and robust methods for determining the purity of scalemic mixtures. To this aim, the main analytical methods of interest are chiral chromatography (chiral gas chromatography^1^ or chiral high-performance liquid chromatography^2,3^), chiral electrophoresis,^4,5^ and chiral spectroscopies.^6^ Among these latter, NMR spectroscopy, a reliable routine technique, has received much attention.^7,8^

The main strategies to determine the enantiomeric composition via NMR are the use of chiral derivatizing agents (CDAs), chiral solvating agents (CSAs), chiral lanthanide shift reagents (CLSRs), or chiral liquid crystals (CLCs).^9−12^ In particular, CSAs are interesting compounds: they are simply added to an analyte solution without the need for time-consuming derivatization steps, and the enantiomeric composition of the chiral compound can be directly determined from a ^1^H NMR spectrum.

Enantiodifferentation relies on secondary interactions, such as ion pairing, π–π, Coulombic and hydrogen bond interactions: diastereomeric adducts are formed *in situ*, by means of a fast (on the NMR time-scale) complexation equilibrium, and hence, the final measured spectrum is a time-average of the bound and unbound form of the substrate. Therefore, if the two enantiomers of the analyte are characterized by different association constants, an additional differentiation of the chemical shift could occur, even though thermodynamic differentiation is not necessary to observe enantiodiscrimination in NMR. Furthermore, given that no covalent derivatization is employed, in principle analytes can be recovered at the end of the analysis. This aspect is of primary importance when difficult-to-access costly compounds are analyzed.^12^

A common strategy for the synthesis of new CSAs relies on the use of simple and easy to functionalize chiral platforms.^7,13−22^ These compounds are easily derivatized, and their enantiodiscrimination properties are modulated by the introduction of suitable functional groups. Rigid structures have proven to be suitable in enhancing the selectivity toward particular analytes,^18,21^ while more flexible structures have been employed to enhance CSA versatility. Usually, the observed chemical-shift differentiation derives from the anisotropy of aromatic groups present in the structure of chiral agents. In fact a tweezer-like bis-thiourea derivative, which possesses the above-discussed structural features, has been successfully used as a CSA.^13^

**Figure 1:**
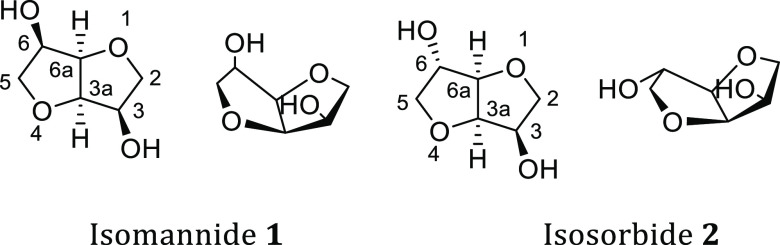
Isomannide (**1**) and isosorbide (**2**).

Starting from these considerations, we reasoned that a fast and easy way to obtain new CSAs could be represented by the use of isohexides. Isohexides, namely (3*R*,3a*R*,6*R*,6a*R*)-hexahydrofuro[3,2-*b*]furan-3,6-diol and (3*R*,3a*R*,6*S*,6a*R*)-hexahydrofuro[3,2-*b*]furan-3,6-diol respectively known as isomannide **1** and isosorbide **2** (Figure 1), are byproducts of the starch industry, arising from dehydration of d-mannitol and d-sorbitol.^23^

These commercially available starting materials provide an easy and inexpensive access to optically pure functionalized compounds. Indeed, through a simple derivatization of the two hydroxyl groups, the characteristic chiral cavity of their scaffold (Figure 1) can be functionalized, thus leading to new compounds, whose properties depend not only on the nature of the introduced moieties but also on the different stereochemistry of the native hydroxyl groups. Due to these characteristics, isohexides were successfully employed as starting materials in the preparation of chiral ligands,^24^ organocatalysts,^25^ and chiral ionic liquids.^26,27^ In particular, starting from isomannide, bidentate ligands^24,28−30^ and ionic molecular tweezers^27,31^ were obtained by virtue of the *endo* arrangement of the hydroxyl groups, which allows the appended units to be sufficiently close to each other. However, the interaction of two appended moieties was even observed in some isosorbide derivatives, due to their particular spatial arrangement.^31^

**Figure 2:**
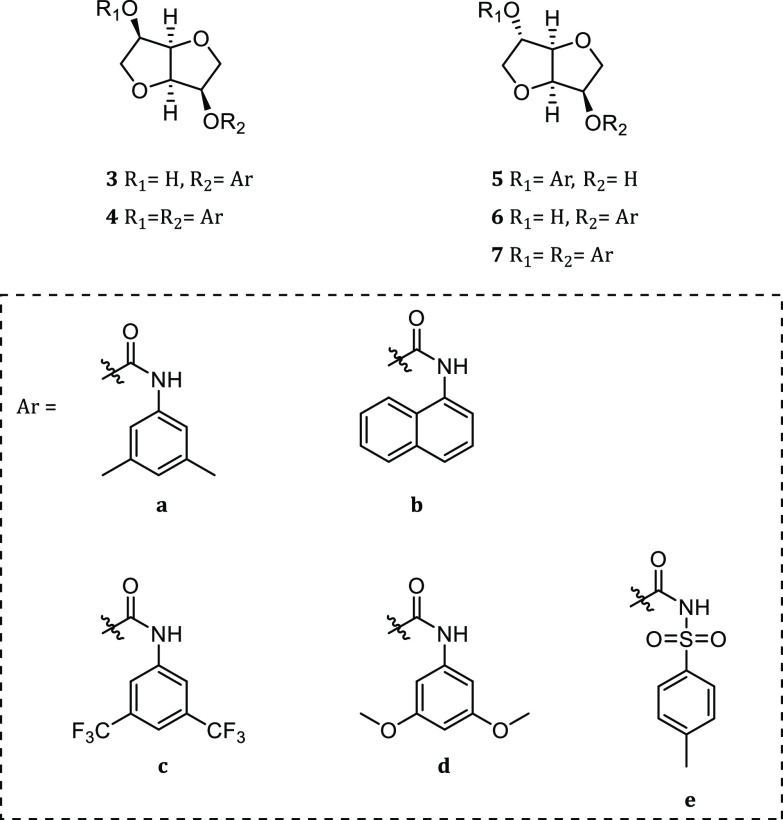
Chiral solvating agents (CSAs) obtained from isomannide **1** and isosorbide **2**.

In our previous works, we demonstrated that some isohexide derivatives could be successfully employed as CSAs in NMR studies.^32,33^ These positive preliminary results prompted us to expand the scope, synthesizing new isohexide derivatives to be employed in NMR enantiodiscrimination studies (Figure 2). In particular, derivatization of the hydroxyl groups as arylcarbamates was chosen to obtain new chiral shift agents that could establish multiple intermolecular interactions, such as π–π interactions through the aromatic groups and dipole–dipole interactions and/or hydrogen bond interactions through the carbamoyl group. To study the influence of different parameters on the enantiorecognition process, such as isohexide stereochemistry and nature, number, and position of the derivatizing moieties on the chiral scaffold, a family of mono- and dicarbamates was easily synthesized from parent isomannide and isosorbide. Arylcarbamoyloxy derivatives, containing respectively electron-donating groups (3,5-dimethyl-, **3a**–**7a**, or 3,5-dimethoxy-, **3d**–**7d**) or electron-withdrawing groups (3,5-bis(trifluoromethyl)-, **3c**–**7c**) were selected to test the influence of opposite electronic effects on enantiodifferentiation. 1-Naphthylcarbamoyloxy derivatives **3b**–**7b** were synthesized to obtain a deeper chiral pocket, and *p*-toluenesulfonylcarbamoyloxy derivatives **3e**–**7e** were endowed with more acidic protons (Figure 2). All products were fully characterized (Figures S1–S55, Supporting Information), and their enantiodiscrimination ability was studied by ^1^H NMR spectroscopy.

## Results and Discussion

### Synthesis of Compounds **3**–**7**

**Scheme 1:**
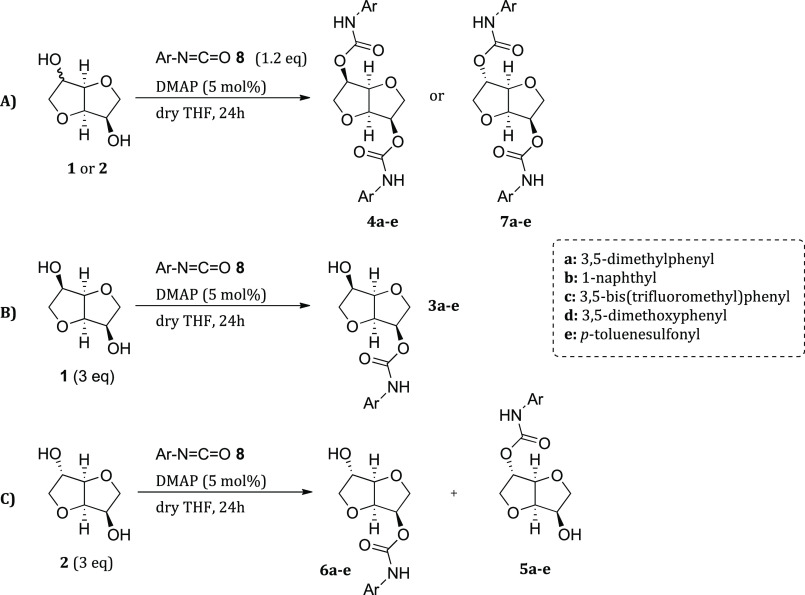
Synthesis of CSAs from Isomannide and Isosorbide: (A) Synthesis of Disubstituted Derivatives **4** and **7**; (B) Synthesis of Monosubstituted Derivatives **3** from Isomannide **1**; and (C) Synthesis of Monosubstituted Derivatives **5** and **6** from Isosorbide **2**

The synthesis of compounds **3**–**7** was performed following a general protocol: isomannide **1** or isosorbide **2** was reacted with an aryl-isocyanate **8** employing a catalytic amount of dimethylaminopyridine (DMAP) in dry tetrahydrofuran as the solvent (Scheme 1). In order to obtain mono- or disubstituted derivatives, a different stoichiometry was employed. For the synthesis of compounds **4** and **7**, an excess of aryl-isocyanate was added, while to enhance the selectivity toward the monoderivatization a 3-fold excess of the reacting isohexide **1** or **2** was used. In both cases, DMAP was employed as the catalyst, except for compounds **3e**, **5e**, and **6e**. While monoderivatization of symmetric isomannide **1** led to one product, reaction of isosorbide **2** afforded a mixture of the two possible isomers **5** and **6** in an ∼1:1 ratio. The nonselective monoderivatization of isosorbide was not an issue, since we were interested in both isomers; a selective derivatization could be easily obtained exploiting an initial selective protection as reported in the literature.^32,34^ In all cases, pure compounds were obtained in good yields after chromatographic purification, with the only exception of **4a**–**d**, **7a**–**c** that crystallized from the crude and/or were recrystallized after a simple workup.

### Enantiodiscrimination Tests

**Figure 3:**
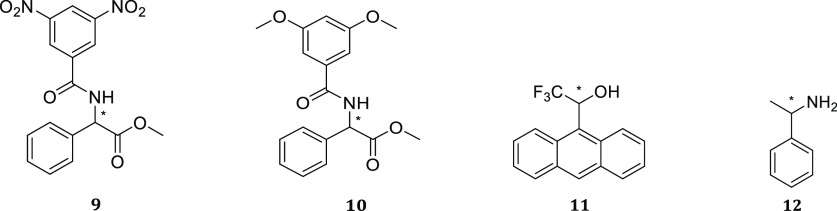
Racemic chiral analytes employed in enantiodiscrimination studies.

Compounds **3**–**7** were tested for their enantiodiscriminating properties toward selected racemic substrates (Figure 3) in ^1^H NMR experiments. In the initial part of the work we focused on the enantiodiscrimination of amino acid derivatives. Initially, *rac*-*N*-3,5-dinitrobenzoylphenylglycine methyl ester (3,5-DNBPhGlyCOOMe, **9**) was used to test the different chiral auxiliaries, as the 3,5-DNB aromatic moiety can establish π–π interactions with the CSAs, leading to an enhancement in enantiodifferentiation. Furthermore, as already reported,^13^ the introduction of a 3,5-DNB moiety allows for having some diagnostic signals to study enantiodiscrimination phenomena, since its protons resonate in a spectral region free from CSA signals (Figure S62, Supporting Information).

Enantiodiscrimination tests were performed by adding 1 equiv of CSA (**3**-**7**) to a 30 mM solution of **9** in CDCl_3_ as the solvent. Splitting of selected NMR signals was employed as a measure of enantiodiscrimination magnitude (Table 1).

**Table 1:**
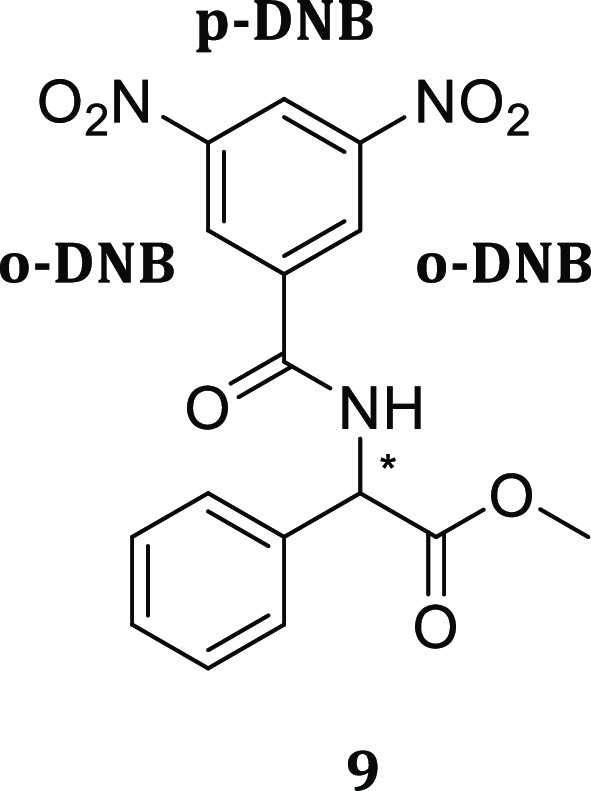
^1^H NMR (500 MHz, CDCl_3_, 21 °C) Nonequivalences (ΔΔδ, ppm)^a^ of Selected Proton Signals of 3,5-DNBPhGlyCOOMe (**9**, 30 mM) in the Presence of an Equimolar Amount of Compounds **3**–**7** (30 mM)

Entry	CSA	*p*-DNB	*o*-DNB	NH	CH	COOMe
1	**3a**	0.010	0.019	0.030	0	0.006
2	**5a**	0	0	0	0	0
3	**6a**	0.020	0.020	0.071	0.003	0.010
4	**4a**	0.005	0.013	0.011	0	0.005
5	**7a**	0.017	0.039	0.111	0.014	0.017
6	**3b**	0.002	0	nd^b^	0.003	0
7	**5b**	0.001	0	0.011	0	0
8	**6b**	0.006	0.002	0.009	0	0
9	**3c**	0.007	0.021	0.041	0.014	0.006
10	**5c**	0	0	0	0	0
11	**6c**	0.010	0.020	0.077	0.014	0.009
12	**4c**	0.005	0.006	0.013	0	0.001
13	**7c**	0.015	0.056	0.159	0.036	0.023
14	**3d**	0.012	0.027	0.050	0.014	0.008
15	**5d**	0	0	0	0	0
16	**6d**	0.018	0.021	0.084	0	0.009
17	**7d**	0.016	0.047	0.136	0.022	0.016

a ΔΔδ = |Δδ_R_ – Δδ_S_| where Δδ_R_ = δ^R^_mixture_ – δ_free_ and Δδ_S_ = δ^S^_mixture_ – δ_free_, being δ^R^_mixture_ and δ^S^_mixture_ the chemical shifts of the two enantiomers in the presence of the CSA.

b Signal not detected due to overlapping with the resonance of aromatic protons.

Compounds **4b**, **7b**, **4d**, and **6e** were completely insoluble in CDCl_3_; therefore, little to moderate amounts (from 30 to 150 μL) of DMSO-*d*_*6*_ were added to accomplish complete dissolution of the CSA. In the presence of the polar coordinating solvent, detectable nonequivalences (0.013 for o-DNB protons and 0.021 for the NH proton) were observed only for **4d**. Because of the different experimental conditions, these data cannot be used to compare the effectiveness of the CSAs. However, DMSO, even to a very low extent, causes a drastic decrease in the nonequivalences measured for a CSA completely soluble in CDCl_3_, as **7c**: in this case the nonequivalence decreased from 0.056 ppm to 0.018 for *o*-DNB protons and dropped from 0.159 to 0.009 ppm for NH proton.

**Figure 4:**
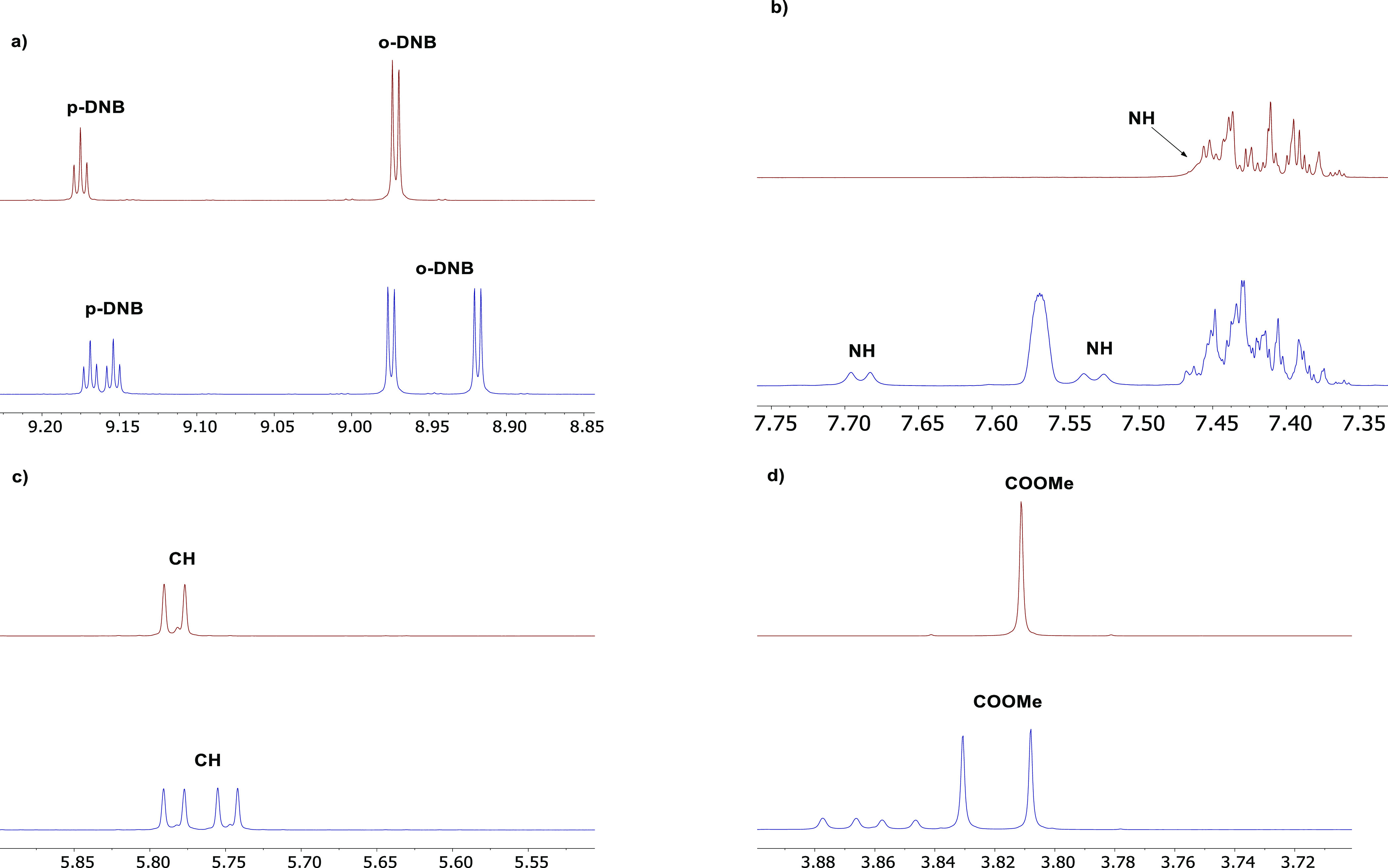
^1^H NMR spectra (500 MHz, CDCl_3_, 21 °C) of **9** (30 mM, red line) and of an equimolar mixture **7c**/**9** (30 mM, blue line): (a) spectral region corresponding to the *para*- and *ortho-*DNB protons of **9**; (b) spectral region corresponding to the NH proton of **9**; (c) spectral region corresponding to the CH proton of **9**; (d) spectral region corresponding to the COOMe of **9**.

Focusing on CSAs’ structures, good results were obtained with derivatives endowed with 3,5-dimethyl (**3a**, **4a**, **6a**, **7a**, *entries 1, 4, 3, and 5* respectively), 3,5-bis(trifluoromethyl) (**3c**, **4c**, **6c**, **7c**, *entries 9, 12, 11, and 13* respectively), or 3,5-dimethoxy (**3d**, **6d**, **7d**, *entries 14, 16, and 17* respectively) substituted aromatic rings. The best results were obtained with derivative **7c** (Table 1, *entry 13* and Figure 4) containing two electron-poor 3,5-bis(trifluoromethyl)phenylcarbamoyloxy groups. This suggests that π–π interactions between electronically complementary aromatic rings play a minor role in the enantiodifferentiation mechanism. Furthermore, very low ΔΔδ values were recorded when using derivatives endowed with sterically hindered 1-naphthylcarbamoyloxy (**3b**, **5b**, and **6b**) or with *p*-toluenesulfonylcarbamoyloxy (**3e**, **4e**, **5e**, and **7e**) groups.

In each series of compounds (i.e., compounds containing the same arylcarbamoyloxy group), a general trend could be observed (Table 1). The best results were obtained with isosorbide derivatives possessing two arylcarbamoyloxy units (**7a**, **7c**, **7d**; *entries 5, 13, and 17* respectively). A deeper analysis suggests that monofunctionalized CSAs, endowed with an *endo* aromatic substituent (**3a**, **3c**, **3d** and **6a**, **6c**, **6d**), work better than those having the same group with *exo* stereochemistry (**5a**, **5c**, **5d**) (compare *entries 1–2–3*, *entries 9–10–11*, and *entries 14–15–16* in Table 1). Furthermore, among compounds possessing an *endo* aromatic substituent, higher nonequivalences could be observed when the free hydroxyl group presented an *exo* stereochemistry, as in derivatives **6a**, **6c**, **6d** (compare *entries 1–3*, *entries 9–11,* and *entries 14–16* in Table 1). Considering derivatives **4** and **7**, the results showed that the presence of two aromatic moieties was beneficial when they have a *trans* arrangement (**7a, c–d**, *entries 5, 13*, and 17), while worse results were observed when these groups were both *endo* (**4a**, **c**, *entries 4 and 12*).

Regarding the signal shift, a common trend could be observed for all the CSAs, showing a low-frequency shift for the *p*-DNB (up to 0.098 ppm) proton and a high-frequency shift for the −NH proton of **9** (up to 0.252 ppm) (Figures S63–64, S66–67, and S69, Supporting Information). This evidence suggests an interaction between the aromatic groups of CSAs and the electron-poor phenyl ring of **9**, with the *p*-DNB proton in the shielding cone of CSAs. It is also possible to assume −NH as one of the major sites of intermolecular interaction, as suggested by the high shifts always observed.

To assess if the higher nonequivalence observed for **7c** derives from a cooperative action of the two aromatic groups, 1:2 mixtures of **9** and monoderivatives **5c** and **6c** (Table 2, *entries 2 and 4*) were analyzed. Doubling the concentration of monoderivative **5c** did not give any enantiodiscrimination (*entry 2*). Conversely, better enantiodifferentiation could be observed for **6c**, but the nonequivalence values were lower than those obtained with compound **7c** (compare *entries 4–5*). These results suggest the cooperative effect of the two substituents in **7c** (Table 2).

**Table 2:** ^1^H NMR (500 MHz, CDCl_3_, 21 °C) Nonequivalences (ΔΔδ, ppm)^a^ of Selected Proton Signals of 3,5-DNBPhGlyCOOMe **9** (30 mM) in the Presence of Compounds **5c**, **6c**, **7c**

Entry	CSA	[CSA]	*p*-DNB^b^	*o*-DNB^c^	NH	CH^d^	COOMe
1	**5c**	30 mM	0	0	0	0	0
2	**5c**	60 mM	0	0	0	0	0
3	**6c**	30 mM	0.010	0.020	0.077	0.014	0.009
4	**6c**	60 mM	0.017	0.032	0.121	0.014	0.015
5	**7c**	30 mM	0.015	0.056	0.159	0.036	0.023

a ΔΔδ = |Δδ_R_ – Δδ_S_| where Δδ_R_ = δ^R^_mixture_ – δ_free_ and Δδ_S_ = δ^S^_mixture_ – δ_free_, being δ^R^_mixture_ and δ^S^_mixture_ the chemical shifts of the two enantiomers in the presence of the CSA.

b *Para-*proton of the 3,5-DNB moiety.

c *Ortho*-protons of the 3,5-DNB moiety.

d Methyne proton of the chiral center.

It is to note that the nonequivalences measured for protons of **9** in the presence of **7c** were comparable^13,35,36^ or even higher^37−39^ than those reported in the literature for the same analyte.

Compound **7c** was then tested with other racemic analytes (**10**–**12**). Nonequivalences were detected only for proton signals of **10**, while 2,2,2-trifluoro-1-(9-anthryl)ethanol **11** or α-methylbenzylamine **12** was not discriminated (Figures S72–S77, Supporting Information). However, ΔΔδ values measured for the proton signals of derivative **10**, the electron-rich analogue of **9**, were lower than those recorded for the protons of **9** (Figure 4 and Figure S73, Supporting Information), so confirming that the π–π interactions between electronically complementary aromatic rings play a minor if not negligible role in the enantiorecognition process.

Derivatives **3e**–**7e**, possessing a more acidic carbamoyl proton, were tested with amine **12** (Table 3). For this substrate, the signals of the proton of the stereocenter and the methyl protons were chosen as diagnostic, since they resonate in sufficiently free spectral regions. In particular, nonequivalence of the signals of the methyl protons was always observed, while the signal of the methine proton was split only employing compounds **4e**, **6e**, **7e** (*entries 5–10*, Table 3). In all cases, a strong interaction between CSAs and the amine can be inferred on the basis of the large shift of the signals (Figure S78–S82, Supporting Information). Compound **4e** was the best CSA for this substrate, leading to good nonequivalences for diagnostic signals. The use of a higher amount of CSAs did not lead to significantly better results, as shown in Table 3.

**Table 3:** ^1^H NMR (500 MHz, CD_2_Cl_3_, 21 °C) Nonequivalences (ΔΔδ, ppm)^a^ Recorded for Selected Proton Signals of α-Methylbenzylamine (**12**, 30 mM) in the Presence of CSAs **3**–**7e**

Entry	CSA	[CSA]	CH^b^	–Me
1	**3e**	30 mM	0	0.016
2	**3e**	60 mM	0	0.012
3	**5e**	30 mM	0	0.006
4	**5e**	60 mM	nd	0.005
5	**6e**	30 mM	0.027	0.012
6	**6e**	60 mM	0.028	0.014
7	**4e**	30 mM	0.041	0.021
8	**4e**	60 mM	0.058	0.017
9	**7e**	30 mM	0.027	0.014
10	**7e**	60 mM	0.029	0.005

a ΔΔδ = |Δδ_R_ – Δδ_S_| where Δδ_R_ = δ^R^_mixture_ – δ_free_ and Δδ_S_ = δ^S^_mixture_ – δ_free_, being δ^R^_mixture_ and δ^S^_mixture_ the chemical shifts of the two enantiomers in the presence of the CSA.

b Methine proton of the stereocenter.

Optimization of enantiodiscrimination tests was performed, aimed at determining the best conditions for the enantiorecognition study, on the best CSA-analyte couple (**7c** and **9**). To this aim 1:1 mixtures of **7c** and **9** at higher concentrations (45 and 60 mM) and 5 mM solutions of **9** containing different amounts of **7c** were analyzed (Table 4, Figure S83–S84 Supporting Information).

**Table 4:**
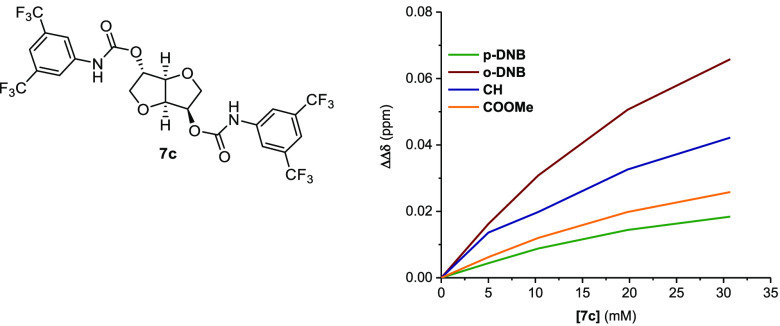
^1^H NMR (500 MHz, CDCl_3_, 2 1°C) Nonequivalences (ΔΔδ, ppm)^a^ for Selected Protons of 3,5-DNBPhGlyCOOMe **9** in the Presence of **7c** (Top Right: ΔΔδ Variation for All the Diagnostic Protons of **9** (5 mM) at Different **7c** Concententrations)

Entry	[**7c**]	[**9**]	*p*-DNB^b^	*o*-DNB^c^	NH	CH^d^	COOMe
1	30 mM	30 mM	0.015	0.056	0.159	0.036	0.023
2	45 mM	45 mM	0.016	0.064	0.168	0.041	0.027
3	60 mM	60 mM	0.018	0.075	0.185	0.045	0.031
4	5 mM	5 mM	0.004	0.016	nd^e^	0.014	0.006
5	10 mM	5 mM	0.009	0.031	nd^e^	0.020	0.012
6	20 mM	5 mM	0.014	0.051	nd^e^	0.033	0.020
7	30 mM	5 mM	0.018	0.066	nd^e^	0.042	0.026

a ΔΔδ = |Δδ_R_ – Δδ_S_| where Δδ_R_ = δ^R^_mixture_ – δ_free_ and Δδ_S_ = δ^S^_mixture_ – δ_free_, being δ^R^_mixture_ and δ^S^_mixture_ the chemical shifts of the two enantiomers in the presence of the CSA.

b *Para-*proton of the 3,5-DNB moiety.

c *Ortho*-protons of the 3,5-DNB moiety.

d Methine proton of the stereocenter.

e Signal not detected due to superimposition with the resonance of aromatic protons.

The nonequivalence undergoes a significant increase in the range 5 mM to 30 mM, whereas only small changes are observed further increasing the concentration up to 60 mM (Table 4, Figure S84, Supporting Information).

Interestingly, for a 5 mM substrate concentration, in the presence of 6 equiv of CSA a 4-fold increase of enantiomer differentiation was obtained (Table 4, Figure S83, Supporting Information). The best results were obtained working with a 60 mM or a 45 mM equimolar solution of **9** and **7c** (Table 4, *entry 3* and *entry 2*). In this case, the signal of −NH proton could be clearly detected, making these two conditions both suitable for enantiodifferantion mechanism studies (Figure S84, Supporting Information). The choice fell on 45 mM solutions to avoid the risk of observing precipitation of the analytes over time and to use a slightly lower amount of the prepared CSA.

Finally, additional experiments have been carried out to compare the enantiomeric ratio (er) in **7c**/**9** mixtures (nominal enantiomeric ratio (*R*)-**9**/(*S*)-**9** 79:21 and 98.5:1.5), on the basis of NMR spectroscopy and chiral chromatography determinations. The results from the two techniques were in very good agreement (Figures S88–S90).

### NMR Characterization of CSA **7c**

**Figure 5:**
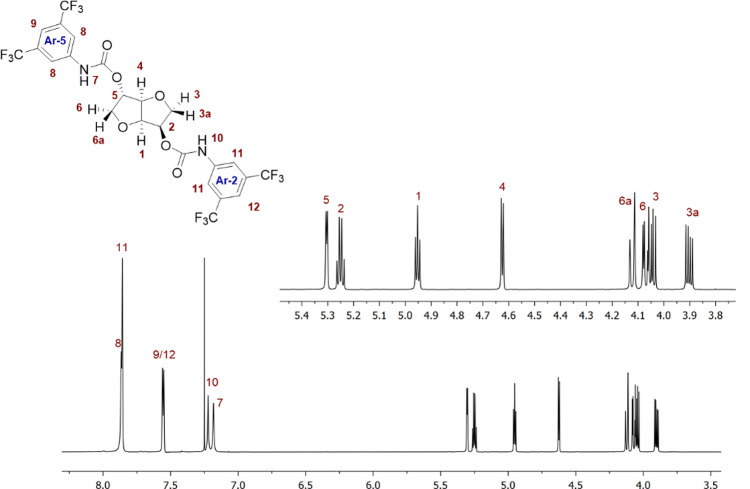
^1^H NMR (600 MHz, CDCl_3_, 45 mM, 25 °C) spectrum of **7c**.

In order to analyze the enantiodiscrimination mechanism between CSA **7c** and compound **9**, complete characterization of CSA **7c** was needed. To this aim, 1D and 2D NMR experiments were performed, in CDCl_3_ at 45 mM concentration. Discussion of homo- and heterocorrelations detected in COSY, ROESY, and HSQC spectra is reported in the Supporting Information. Characterization data are collected in Table S1, Supporting Information and reported in Figure 5.

Considering the conformation of CSA **7c** in solution, the two 3,5-bis(trifluoromethyl)phenyl moieties bound to the rigid core did not show any particular conformational prevalence: as an example, the magnitude of ROE effects given by the NH-7 proton at the frequencies of H_5_, H_1_, H_6a_, and H_4_ were comparable (Figure S86b, Supporting Information), indicating that the 3,5-bis(trifluoromethyl)phenyl moiety bound to the C_5_ site, named Ar-5, is freely rotating around the C_5_–O bond. Analogously, the NH-10 proton gave dipolar interactions with protons H_1_, H_2_, and H_3_ (Figure S86b, Supporting Information). These last effects were once again comparable in magnitudes; therefore, the 3,5-bis(trifluoromethyl)phenyl moiety bound to the C_2_ carbon, named Ar-2, is itself freely rotating around the C_2_–O bond.

### Interaction Mechanism in the Diastereomeric Pairs (*S*)-3,5-DNBPhGlyCOOMe/**7c** and (*R*)-3,5-DNBPhGlyCOOMe/**7c**

**Figure 6:**
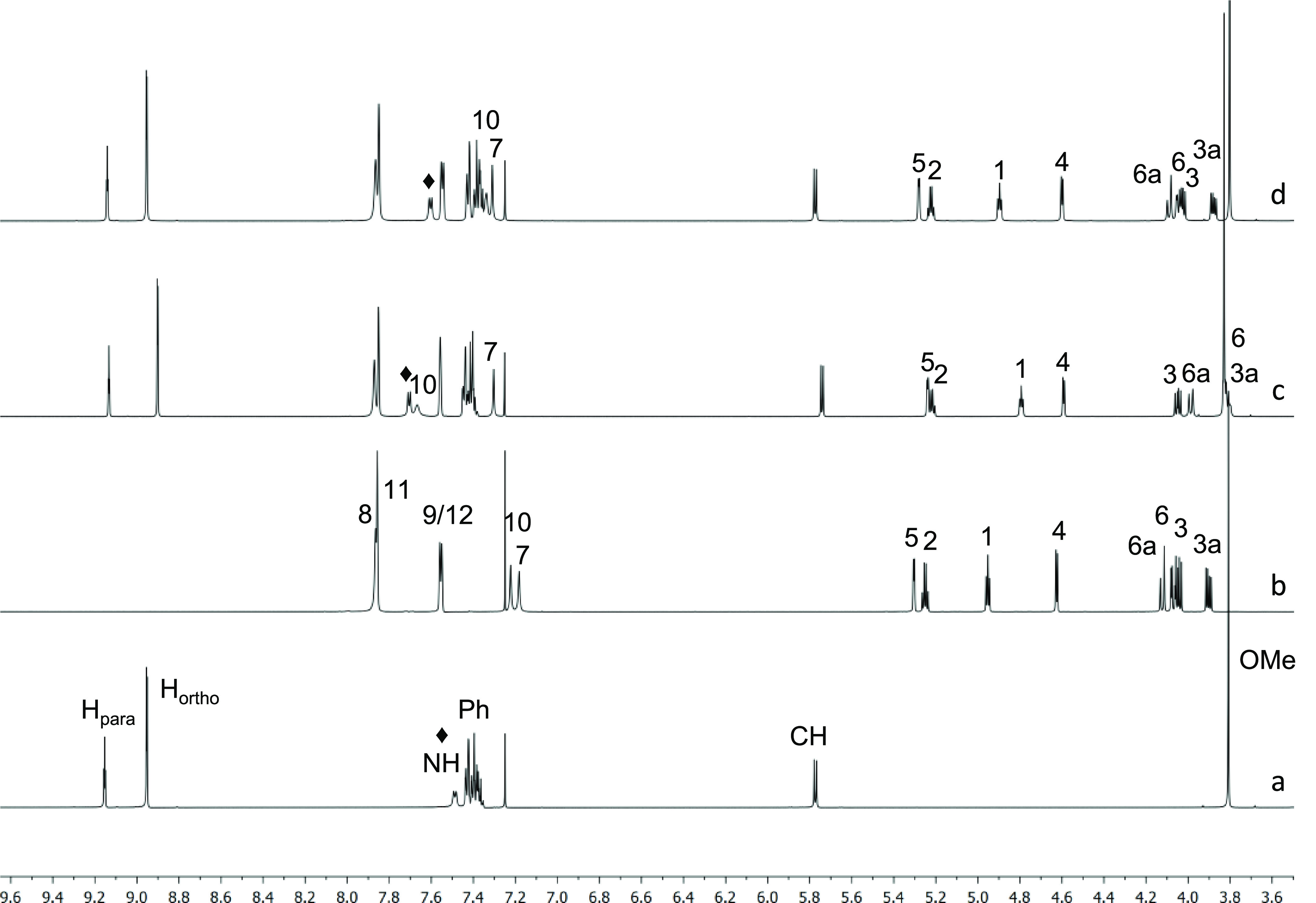
^1^H NMR (600 MHz, CDCl_3_, 25 °C) spectra of (a) 3,5-DNBPhGlyCOOMe (**9**, 45 mM); (b) **7c** (45 mM); (c) **7c**/(*R*)-**9** (1:1, total concentration 90 mM); (d) **7c**/(*S*)-**9** (1:1, total concentration 90 mM).

**Figure 7:**
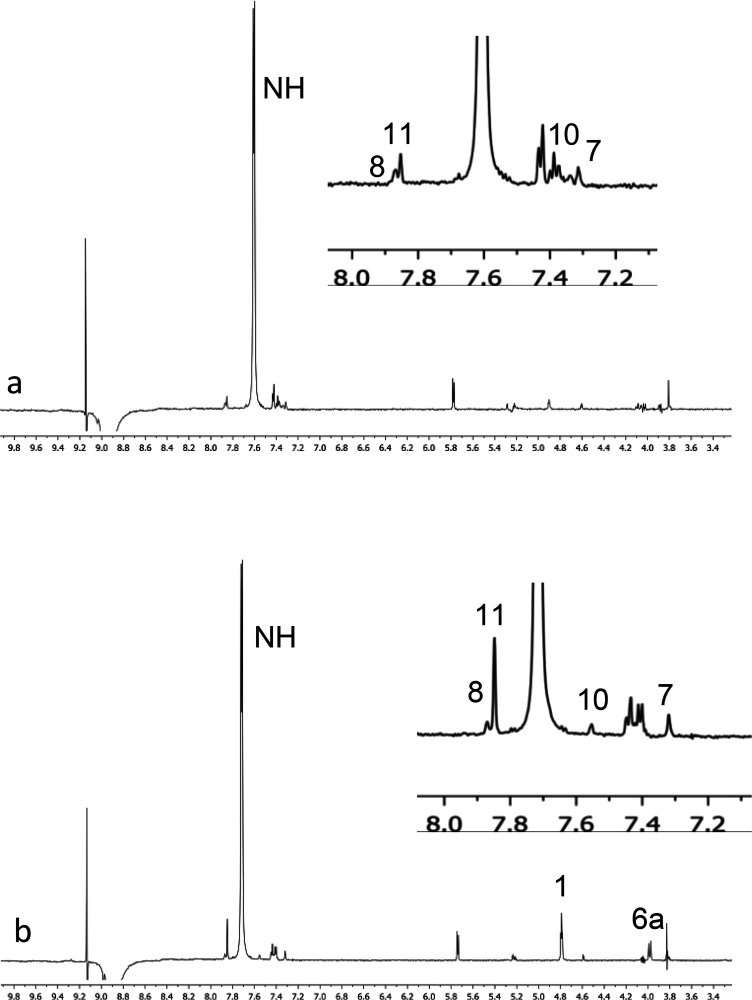
1D ROESY (600 MHz, CDCl_3_, 25 °C, mix = 0.5 s) spectra corresponding to the perturbation of ortho protons of 3,5-dinitrophenyl moiety of 3,5-DNBPhGlyCOOMe (**9**, 45 mM) in the presence of 1 equiv of **7c** for (a) **7c**/(*S*)-**9** and (b) **7c**/(*R*)-**9**.

Information regarding the nature of intermolecular interactions responsible for chiral discrimination in solution was obtained on the basis of the analysis of complexation shifts (Δδ = δ_mix_ – δ_free_, ppm) and intermolecular ROE effects detected in equimolar mixtures CSA/(*S*)-3,5-DNBPhGlyCOOMe (**7c**/(*S*)-**9**) and CSA/(*R*)-3,5-DNBPhGlyCOOMe (**7c**/(*R*)-**9**) at the concentration 45 mM (Tables 5 and 6 and Figures 6 and 7).

**Table 5:** ^1^H NMR (600 MHz, CDCl_3_, 25 °C) Complexation Shifts (Δδ = δ_mix_ – δ_free_, ppm) of CSA **7c** (45 mM) in the Presence of 1 equiv of 3,5-DNBPhGlyCOOMe **9**

Δδ		
Proton	(*S*)-3,5-DNBPhGlyCOOMe	(*R*)-3,5-DNBPhGlyCOOMe
1	–0.052	–0.160
2	–0.026	–0.032
3	–0.018	+0.003
*3a*	–0.023	–0.081
4	–0.026	–0.033
5	–0.024	–0.069
6	–0.022	–0.260
*6a*	–0.033	–0.135
*NH-7*	+0.127	+0.123
8	0	+0.006
*NH-10*	+0.116	+0.444
11	–0.008	–0.006

**Table 6:** ^1^H NMR (600 MHz, CDCl_3_, 25 °C) Complexation Shifts (Δδ = δ_mix_ – δ_free_, ppm) of (*S*)- and (*R*)-3,5-DNBPhGlyCOOMe (**9**, 45 mM) in the Presence of 1 equiv of CSA **7c**

Δδ		
Proton	(*S*)-3,5-DNBPhGlyCOOMe	(*R*)-3,5-DNBPhGlyCOOMe
*CH*	0	–0.035
*NH*	+0.116	+0.219
*OMe*	–0.006	+0.021
*H*_*orthoDNB*_	0	–0.054
*H*_*paraDNB*_	–0.010	–0.018

Compound (*R*)-**9** showed a strong preference for the interaction at the 3,5-bis(trifluoromethyl)phenyl moiety Ar-2. In fact, a remarkably high complexation shift of +0.444 ppm was measured for the proton NH-10, as well as very high complexation shifts were measured for the protons H_1_ and H_6_ (Table 5, Figure 6). Interestingly, negligible complexation shifts were measured for the protons H_11_/H_8_ of the 3,5-bis(trifluoromethyl)phenyl group (Table 5). Therefore, the intermolecular adduct is mainly stabilized by a strong network of hydrogen-bond interactions involving the hydrogen-bond donor group NH-10 of the CSA and, reasonably, the electron-acceptor oxygen atoms of its rigid isosorbide skeleton. Every proton of (*R*)-**9** showed relevant complexation shifts in the presence of the CSA, with higher values for its NH proton (Table 6). Even if a strong preference for the interaction at the NH-10 moiety of Ar-2 can be assessed, the interaction must also involve moiety Ar-5, although to a minor extent, as witnessed by the complexation shifts measured for NH-7 (+0.123 ppm), H_6a_ (Δδ = −0.135 ppm), and H_5_ (Δδ = −0.069 ppm). The very high shift value of −0.260 ppm measured for H_6_ probably comes from the contribution of the interaction both at the NH-10 and NH-7, which likely entails closeness of the aromatic moiety of (*R*)-**9** to the isosorbide skeleton causing shielding of proton H_6_.

The interaction of CSA **7c** with (*S*)-**9** involves once again both the NH-7 and NH-10 moieties, with a slight preference for NH-7 (Table 5). Only NH proton of (*S*)-**9** showed significant complexation shift (Table 6). Therefore, it can be concluded that the acidic NH group of moiety Ar-5 is mainly involved in nonselective interactions with both enantiomers, whereas the enantiodiscrimination mainly originates from the strong preference of NH-10 for (*R*)-**9**.

The above conclusions were supported also by the nature of proximity constraints arising from intermolecular ROE effects detected in the two mixtures. In particular, relevant inter-ROEs were found between the ortho protons of (*R*)-**9** and proton H_1_, H_6a_, NH-7, and NH-10 (Figure 7b). The ROE effect at H_11_ was more intense than it was at H_8_, confirming that the 3,5-dinitrophenyl group of the substrate mainly lies on the rigid skeleton of the CSA with a preference for the interaction at NH-10 of Ar-2.

Analogous but weaker and less selective intermolecular ROE effects were detected in the mixture (*S*)-**9**/**7c**, indicating a minor preference for the interaction at NH-10 of Ar-2 (Figure 7a).

**Figure 8:**
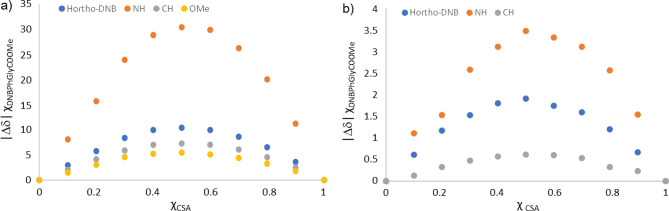
Job plots derived from ^1^H NMR spectra (600 MHz, CDCl_3_, total concentration of 45 mM, 25 °C): (*R*)-**9**/**7c** (a) and (*S*)-**9**/**7c** (b).

Complexation stoichiometries of the two complexes CSA/(*S*)-**9** and CSA/(*R*)-**9** were established by using Job’s method.^40^ By plotting the complexation shifts (Δδ) of selected protons of derivative **9** multiplied by its molar fraction (χ_DNBPhGlyCOOMe_) versus the molar fraction of CSA **7c** (χ_CSA_), symmetrical bell curves with a maximum at χ_CSA_ = 0.5 were obtained for protons H_ortho-DNB_/NH/CH of both enantiomers of 3,5-DNBPhGlyCOOMe **9**, indicating a well-defined 1-to-1 interaction (Figure 8).

**Figure 9:**
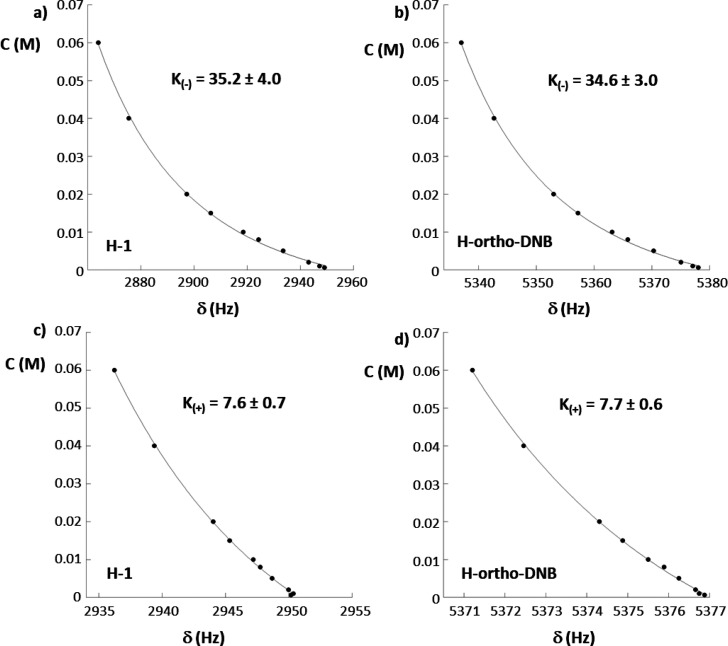
Association constants determination based on dilution data in (*R*)-**9**/**7c** mixtures for H_1_ of CSA **7c** (a) and H_ortho_-DNB of amino acid **9** (b) and in (*S*)-**9**/**7c** mixtures for H_1_ of CSA **7c** (c) and H_ortho_-DNB of amino acid **9** (d).

Finally, association constants determined by dilution data (Figure 9) were calculated: ca. 35 M^–1^ for (*R*)-**9**/**7c** and ca. 7 M^–1^for (*S*)-**9**/**7c**.

## Conclusions

A new family of chiral solvating agents (CSAs) **3**–**7** was easily synthesized starting from isomannide and isosorbide. Following the same protocol, by a single synthetic step new mono- and disubstituted carbamates were easily obtained and purified. Different phenyl isocyanates were selected to test different aspects, such as the influence of opposite electronic effects, the elongation of the arms of the chiral clamp, and the influence of the acidity of carbamic −NH on the enantiodiscrimination.

All the prepared CSAs were tested employing *rac*-*N*-3,5-dinitrobenzoylphenylglycine methyl ester **9** as a representative analyte. The results clearly showed that the chiral structure of isohexides is well suitable for building-up chiral auxiliaries that can be successfully employed in enantiodiscrimination processes. The large portfolio of derivatives allowed us to study the influence of different parameters, such as stereochemistry and degree of derivatization of the central chiral scaffold as well as structural and electronic properties of the derivatizing agent, on the enantiodiscriminating capabilities. The cooperative action of two derivatizing moieties, the interaction with the NH groups, the minor role played by π–π interaction between electronically complementary aromatic rings emerged as peculiar characteristics of the enantiorecognition and the best nonequivalences were obtained with derivative **7c** containing two electron-poor 3,5-bis(trifluoromethyl)phenylcarbamoyloxy groups.

The study of the enantiodiscrimination mechanism allowed us to establish that (*R*)-**9** showed a stronger interaction with CSA **7c** than its enantiomer, with a strong preference for the interaction with one of the 3,5-bis(trifluoromethyl)phenyl moieties of the CSA and with the intermolecular adduct being mainly stabilized by a strong network of hydrogen bonds interactions. In particular, enantiodiscrimination mainly originated from the NH-10 preference for (*R*)-**9**.

## Experimental Section

### Materials and General Methods

All the reactions involving sensitive compounds were carried out under dry Ar, in flame-dried glassware. If not noted otherwise, reactants and reagents were commercially available and used as received from TCI-Chemicals and Sigma-Aldrich. TLC analyses were carried out with Merk 60 F254 plates (0.2 mm). ^1^H NMR spectra were recorded in Chloroform-*d*, Acetone-*d*_*6*_, Methanol-*d*_*4*_, and DMSO-*d*_*6*_ on a JEOL ECZ400S or JEOL ECZ500R spectrometer. The following abbreviations are used: s = singlet, bd = broad singlet, d = doublet, dd = double doublet, ddd = double double doublet, dt = double triplet, t = triplet, td = triple doublet, tdd = triple double doublet, q = quartet, h = heptet m = multiplet. ^13^C NMR spectra were recorded at 101 MHz on a JEOL ECZ400S or at 126 MHz on a JEOL ECZ500R spectrometer. ^19^F spectra were recorded at 471 MHz on a JEOL ECZ500R spectrometer. ^1^H and ^13^C NMR chemical shifts (ppm) are referred to TMS as the external standard. Melting points were measured on a Reichert Thermovar Type 300429 Microscope. Optical rotations were measured in 1 dm cells at the sodium D line, using an Anton Paar MCP 300 Polarimeter. NMR characterization of compound **7c** and the study of the interaction mechanism were performed on an INOVA600 spectrometer operating at 600 MHz for ^1^H nuclei. The samples were analyzed in CDCl_3_ solution; the temperature was controlled (25 °C). For all the 2D NMR spectra the spectral width used was the minimum required in both dimensions. The gCOSY (gradient COrrelation SpectroscopY) map was recorded by using a relaxation delay of 1 s, 128 increments of 8 transients, each with 2K points. The 2D-ROESY (Rotating-frame Overhauser Enhancement SpectroscopY) maps were recorded by using a relaxation time of 3 s and a mixing time of 0.5 s; 128 increments of 16 transients of 2K-points each were collected. The 1D-ROESY spectra were recorded using a selective inversion pulse with 1024 transients, a relaxation delay of 1 s, and a mixing time of 0.5 s. The gHSQC (gradient Heteronuclear Single Quantum Coherence) map was recorded with a relaxation time of 1.2 s, 128 increments with 32 transients, each of 2K-points. Elemental analyses were obtained using an Elementar Vario MICRO cube equipment.

HPLC analyses were performed on a JASCO PU-1580 intelligent HPLC pump equipped with a JASCO UV-975 detector. The column temperature was controlled with a JASCO HPLC Column oven.

Isomannide (**1**), isosorbide (**2**), aryl isocyanates (**8**), *rac*-propylene oxide, 3,5-dinitrobenzoyl chloride, 3,5-dimethoxybenzoyl chloride, and diazabiciclo[5,4,0]undec-7-en (DBU) were purchased from Merck and used as received.

### General Procedure for the Synthesis of Derivatives **3**–**7**

Under an Ar atmosphere, phenyl aryl isocyanate **8** and 4-(dimethylamino)pyridine (DMAP) were added to a solution of isohexide **1** or **2** in dry THF. The reaction was followed by TLC analysis, and the crude was processed as described in the Supporting Information.

## Supporting Information Available

Detailed procedures for the synthesis and characterization of compounds **3**–**7**, **9**–**10**. ^1^H, ^13^C, and ^19^F NMR spectra of compounds **3**–**7**, **9**–**10** (Figures S1–S61). Enantiodiscrimination tests on compound **9** employing CSAs **3**–**7** (Figures S62–S71). Enantiodiscrimination tests on compounds **10**–**12** employing CSA **7c** (Figures S72–S77). Enantiodiscrimination tests on compound **12** employing CSAs **3e**–**7e** (Figures S78–S82). Optimization of enantiodiscrimination conditions of compound **9** employing CSA **7c** (Figures S83–S84). NMR characterization of compound **7c**: ^1^H–^13^C HSQC map (Figure S85), COSY and ROESY maps (Figure S86), 1D ROESY spectra (Figure S87), ^1^H NMR characterization data (Table S1). Details for the determination of the association constants (p S81). Determination of enantiomeric ratio in scalemic mixtures of **9** by chiral chromatography (HPLC) and ^1^H NMR employing **7c** as CSA (Figures S88–S90). (PDF)
