# Supplementary material for: Renewable Resources for Enantiodiscrimination: Chiral Solvating Agents for NMR Spectroscopy from Isomannide and Isosorbide
Source: J Org Chem. 2022 Sep 8;87(19):12698–709. doi: 10.1021/acs.joc.2c01244 (PMC9552179; doi:10.1021/acs.joc.2c01244)
Supplement: Supplementary file 1 — jo2c01244_si_001.pdf [file jo2c01244_si_001.pdf]

Supporting information

Renewable resources for enantiodiscrimination: chiral solvating agents for NMR spectroscopy from isomannide and isosorbide

Federica Balzano*, Anna Iuliano, Gloria Uccello-Barretta and Valerio Zullo*

Dipartimento di Chimica e Chimica Industriale, Università di Pisa, Via Giuseppe Moruzzi, 13, 56124, Pisa, Italy, e-mail: federica.balzano@unipi.it, valerio.zullo@phd.unipi.it

Table of contents

General procedure for the synthesis of compounds **3** 3

General procedure for the synthesis of compounds **5** and **6** 6

General procedure for the synthesis of compounds **4** and **7** 11

Synthesis of phenylglycine methylesters (compounds **9** and **10**) 18

^{1}H, ^{13}C and ^{19}F NMR spectra of compounds **3-7** and of compounds **9-10** 22

Enantiodiscrimination tests on compound **9** employing CSAs **3-7** 55

Enantiodiscrimination tests on compounds **10-12** employing CSA **7c** 65

Enantiodiscrimination tests on compound **12** employing CSAs **3e-7e** 70

Optimization of enantiodiscrimination conditions of compound **9** employing CSA **7c**...... 75

NMR characterization of CSA **7c**.......... 77

Determination of association constants of the diastereomeric complexes 82

Determination of enantiomeric ratio in scalemic mixtures of **9** by chiral chromatography (HPLC) and ^{1}H NMR employing **7c** as CSA....83

References....86

*General procedure for the synthesis of compounds **3***

Under an Ar atmosphere, phenyl isocyanate **8** (1 equiv.) and 4-(dimethylamino)pyridine (DMAP) (0.05 equivs) were added to a solution of isomannide **1** (6 equivs.) in dry THF (8 mL/equiv. of **8**). The reaction was monitored by TLC analysis. After 18 h the solvent was removed under reduced pressure, the crude was dissolved in CH_2Cl_2 (15 mL) and washed with water (3x10 mL). The organic phase was dried over anhydrous Na_2SO_4 and the solvent was removed under pressure to give the crude product. The crude was processed as described in the following sections.

(3R,3aR,6R,6aR)-hexaydrofuro[3,2-b]furan-6-(3,5-dimethylphenyl)carbamoyl-3-ol (3a)

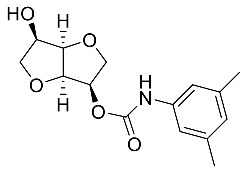

Isomannide **1** (1.34 g, 9.2 mmol), dry THF (25 mL), 3,5-dimethylphenylisocyanate **8a** (500 μL, 3.6 mmol), DMAP (17 mg, 0.1 mmol). TLC analysis (CH_2Cl_2:Acetone 9:1). The crude was purified by Biotage® Isolera (CH_2Cl_2:Acetone) to give the chemically pure product **3a** as a white solid (713 mg, 69%).

R_f 0.17 (CH_2Cl_2:Acetone 9:1). **^{1}H NMR** (500 MHz, Chloroform-*d*) δ 7.00 (s, 2H), 6.75 (bs, 1H), 6.73 – 6.71 (m, 1H), 5.20 (ddd, *J* = 7.3, 6.6, 5.2 Hz, 1H), 4.71 (t, *J* = 5.0 Hz, 1H), 4.54 (t, *J* = 5.2 Hz, 1H), 4.34 (q, *J* = 6.0 Hz, 1H), 4.21 (dd, *J* = 9.1, 6.6 Hz, 1H), 4.00 (dd, *J* = 9.3, 6.1 Hz, 1H), 3.84 (dd, *J* = 9.1, 7.3 Hz, 1H), 3.67 (dd, *J* = 9.4, 6.4 Hz, 1H), 2.29 (d, *J* = 0.7 Hz, 6H), 1.92 (bs, 1H). **^{13}C{^{1}H} NMR** (126 MHz, DMSO-*d_6*) δ 152.8, 139.0, 137.7, 124.1, 116.0, 81.5, 80.2, 74.4, 72.1, 71.6, 69.9, 21.2. **m.p.** 146-149 °C. **[α]$_D^{25°C}$**= +92.0 (c= 0.493, $CHCl_3$). **Elemental Analysis:** Calcd. for $C_{15}H_{19}NO_5$: C, 61.42; H, 6.53; N, 4.78; O, 27.27. Found C, 61.09; H, 6.76; N, 4.85.

(3R,3aR,6R,6aR)-hexaydrofuro[3,2-b]furan-6-(1-naphthyl)carbamoyl-3-ol (3b)

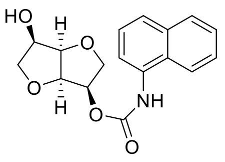

Isomannide **1** (1.37 g, 9.4 mmol), dry THF (100 mL), 1-naphthylisocyanate **8b** (450 µL, 3.1 mmol), DMAP (20 mg, 0.2 mmol). TLC analysis (CH_2Cl_2: Acetone 9:1). The crude was purified by Biotage® Isolera (CH_2Cl_2:Acetone) to give the chemically pure product **3b** as a white solid (550 mg, 56%).

R_f 0.24 (CH_2Cl_2:Acetone 9:1). **^{1}H NMR** (500 MHz, DMSO-d_6) δ 9.71 (bs, 1H), 8.12 – 8.07 (m, 1H), 7.95 – 7.90 (m, 1H), 7.76 (d, *J* = 8.1 Hz, 1H), 7.58 (d, *J* = 7.4 Hz, 1H), 7.55 – 7.51 (m, 2H), 7.49 (t, *J* = 7.9 Hz, 1H), 5.09 (q, *J* = 6.2 Hz, 1H), 4.95 (d, *J* = 6.5 Hz, 1H), 4.67 (t, *J* = 5.1 Hz, 1H), 4.32 (t, *J* = 4.9 Hz, 1H), 4.19 – 4.12 (m, 1H), 3.99 (dd, *J* = 9.2, 6.3 Hz, 1H), 3.86 (t, *J* = 7.5 Hz, 1H), 3.83 – 3.77 (m, 1H), 3.49 (bs, 1H). **^{13}C{^{1}H} NMR** (126 MHz, DMSO-d_6) δ 154.4, 133.8, 133.7, 128.1, 128.1, 126.1, 125.8, 125.6, 125.2, 122.9, 121.5, 81.5, 80.4, 74.7, 72.1, 71.5, 70.1. **m.p.** 161-163 °C. **$[\alpha]_D^{25°C}$**= +72.7 (c= 0.520, $CHCl_3$). **Elemental Analysis:** Calcd. for $C_{17}H_{17}NO_5$: C, 64.75; H, 5.43; N, 4.44; O, 25.3. Found C, 64.36; H, 5.61; N, 4.49.

(3R,3aR,6R,6aR)-hexaydrofuro[3,2-b]furan-6-(3,5-bis(trifluoromethyl)phenyl) carbamoyl-3-ol (3c)

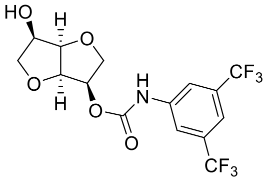

Isomannide **1** (1.33 g, 9.1 mmol), dry THF (100 mL), 3,5-bis-(trifluoromethyl)phenylisocyanate **8c** (500 µL, 2.9 mmol), DMAP (20 mg, 0.2 mmol). TLC analysis (CH_2Cl_2: Acetone 9:1). The crude was purified by Biotage® Isolera (CH_2Cl_2:Acetone) to give the chemically pure product **3c** as a glassy white solid (791 mg, 68%).

R_f 0.31 (CH_2Cl_2:Acetone 9:1). **^{1}H NMR** (500 MHz, Chloroform-*d*) δ 7.88 (s, 2H), 7.57 – 7.55 (m, 1H), 7.42 (s, 1H), 5.27 – 5.20 (m, 1H), 4.77 (t, *J* = 5.2 Hz, 1H), 4.57 (t, *J* = 5.3 Hz, 1H), 4.35 (q, *J* = 5.9 Hz, 1H), 4.20 (dd, *J* = 9.5, 6.3 Hz, 1H), 4.01 (dd, *J* = 9.4, 5.9 Hz, 1H), 3.92 (dd, *J* = 9.5, 6.4 Hz, 1H), 3.69 (dd, *J* = 9.3, 6.3 Hz, 1H), 2.67 (s, 1H). **^{19}F NMR** (471 MHz, Chloroform-*d*) δ -

63.02. **$^{13}C\{^1H\}$ NMR** (126 MHz, Chloroform-*d*) δ 152.3, 139.3, 132.6 (q, *J* = 33.6 Hz), 123.2 (q, *J* = 272.8 Hz), 118.3 (bs), 117.0 (h, *J* = 3.9 Hz), 82.0, 80.9, 75.0, 74.4, 72.2, 71.0. **m.p.** 103-106 °C. **$[\alpha]_D^{25°C}$**= +73.4 (c= 0.593, $CHCl_3$).). **Elemental Analysis:** Calcd. for $C_{15}H_{13}F_6NO_5$: C, 44.90; H, 3.27; F, 28.41; N, 3.49; O, 19.94 Found C, 44.62; H, 3.51; N, 3.19.

(3R,3aR,6R,6aR)-hexaydrofuro[3,2-b]furan-6-(3,5-dimethoxyphenyl)carbamoyl-3-ol (3d)

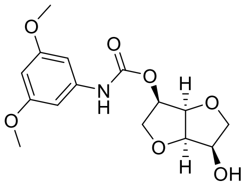

Isomannide **1** (1.38 g, 9.4 mmol), dry THF (100 mL), 3,5-dimethoxyphenylisocyanate **8d** (555 mg, 3.1 mmol), DMAP (24 mg, 0.2 mmol). TLC analysis (CH_2Cl_2: Acetone 9:1). The crude was purified by Biotage® Isolera (CH_2Cl_2:Acetone) to give the chemically pure product **3d** as a white foam (801 mg, 79%).

R_f 0.13 (CH_2Cl_2:Acetone 9:1). **^1H NMR** (500 MHz, Chloroform-*d*) δ 6.86 (bs, 1H), 6.60 (d, *J* = 2.2 Hz, 2H), 6.20 (t, *J* = 2.2 Hz, 1H), 5.20 (ddd, *J* = 7.2, 6.5, 5.2 Hz, 1H), 4.71 (t, *J* = 5.0 Hz, 1H), 4.54 (t, *J* = 5.2 Hz, 1H), 4.34 (q, *J* = 6.1 Hz, 1H), 4.20 (dd, *J* = 9.1, 6.5 Hz, 1H), 4.00 (dd, *J* = 9.3, 6.1 Hz, 1H), 3.87 – 3.80 (m, 1H), 3.77 (s, 6H), 3.66 (dd, *J* = 9.3, 6.4 Hz, 1H), 2.17 (bs, 1H). **$^{13}C\{^1H\}$ NMR** (126 MHz, Chloroform-*d*) δ 161.2, 152.5, 139.5, 97.1, 96.0, 81.8, 80.9, 74.4, 74.1, 72.3, 70.7, 55.4. **m.p.** 118-120 °C. **$[\alpha]_D^{25°C}$**= +84.9 (c= 0.638, $CHCl_3$). **Elemental Analysis:** Calcd. for $C_{15}H_{19}NO_7$: C, 55.38; H, 5.89; N, 4.31; O, 34.43. Found C, 54.99; H, 6.13; N, 4.05.

(3R,3aR,6R,6aR)-hexaydrofuro[3,2-b]furan-6-(p-toluensulfonyl)carbamoyl-3-ol (3e)

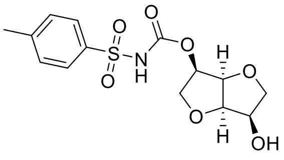

Isomannide **1** (1.37 g, 9.4 mmol), dry THF (100 mL), p-toluensulfonylisocyanate **8e** (460 µL, 3.0 mmol). TLC analysis ($CHCl_3$:MeOH 97:3, 1% HCOOH). The crude was purified by Flash chromatography on silica gel ($CHCl_3$:MeOH 97:3, 1% HCOOH) to give the chemically pure product **3e** as a white solid (463 mg, 45%).

Rf 0.18 ($CHCl_3$:MeOH 97:3, 1% HCOOH). **^{1}H NMR** (500 MHz, Chloroform-*d*) δ 7.97 – 7.89 (m, 3H), 7.35 (d, *J* = 8.1 Hz, 2H), 5.07 (q, *J* = 5.8 Hz, 1H), 4.63 (t, *J* = 5.2 Hz, 1H), 4.44 (t, *J* = 5.3 Hz, 1H), 4.26 (q, *J* = 6.1 Hz, 1H), 4.04 (dd, *J* = 9.8, 5.9 Hz, 1H), 3.89 (dd, *J* = 9.3, 6.1 Hz, 1H), 3.82 (dd, *J* = 9.8, 5.8 Hz, 1H), 3.46 (dd, *J* = 9.3, 6.8 Hz, 1H), 2.56 (bs, 1H), 2.45 (s, 3H). **^{13}C{^{1}H} NMR** (126 MHz, Methanol-*d_4*) δ 152.6, 146.0, 137.9, 130.6, 129.1, 83.0, 82.2, 77.6, 73.3, 72.6, 72.0, 21.5. **m.p.** 158 - 159 °C. **[α]$_D^{25°C}$**= +63.1 (c= 0.390, $CHCl_3$). **Elemental Analysis:** Calcd. for $C_{14}H_{17}NO_7S$: C, 48.97; H, 4.99; N, 4.08; O, 32.62; S, 9.34. Found C, 48.57; H, 5.22; N, 4.28.

*General procedure for the synthesis of compounds **5** and **6***

Under an Ar atmosphere, phenyl isocyanate **8** (1 equiv.) and 4-(dimethylamino)pyridine (DMAP) (0.05 equivs.) were added to a solution of isosorbide **1** (6 equivs.) in dry THF (8 mL/equiv of **8**). The reaction was monitored by TLC analysis. After 18 h the solvent was removed under reduced pressure, the crude was dissolved in CH_2Cl_2 (15 mL) and washed with water (3x10 mL). The organic phase was dried over anhydrous Na_2SO_4 and the solvent was removed under pressure to give the crude product. The crude was processed as described in the following sections.

(3R,3aR,6S,6aR)-hexaydrofuro[3,2-b]furan-6-(3,5-dimethylphenyl)carbamoyl-3-ol (5a) and (3R,3aR,6S,6aR)-hexaydrofuro[3,2-b]furan-3-(3,5-dimethylphenyl) carbamoyl-6-ol (6a)

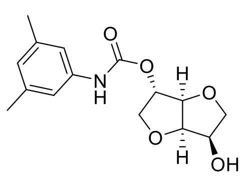

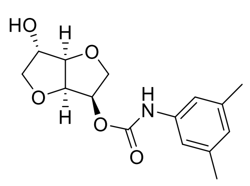

Isosorbide **2** (1.33 g, 9.1 mmol), dry THF (25 mL), 3,5-dimethylphenylisocyanate **8a** (500 μL, 3.6 mmol), DMAP (21 mg, 0.2 mmol). TLC analysis (CH_2Cl_2: Acetone 9:1). The crude was purified by Biotage® Isolera (CH_2Cl_2:Acetone) to give chemically pure **5a** as a glassy white solid (387 mg, 37%) and chemically pure **6a** as a white foam (290 mg, 28%).

5a: **R_f** 0.22 (CH_2Cl_2:Acetone 9:1). **^{1}H NMR** (500 MHz, Chloroform-*d*) δ 6.99 (s, 2H), 6.75 – 6.72 (m, 1H), 6.56 (bs, 1H), 5.26 (d, *J* = 2.8 Hz, 1H), 4.65 (t, *J* = 4.9 Hz, 1H), 4.55 (dt, *J* = 4.4, 1.0 Hz, 1H), 4.33 (q, *J* = 5.8 Hz, 1H), 4.14 (d, *J* = 10.7 Hz, 1H), 4.03 (dd, *J* = 10.7, 3.5 Hz, 1H), 3.91 (dd, *J* = 9.5, 6.0 Hz, 1H), 3.59 (dd, *J* = 9.5, 5.9 Hz, 1H), 2.29 (d, *J* = 0.7 Hz, 6H), 1.85 (bs, 1H). **^{13}C{^{1}H} NMR** (126 MHz, Chloroform-*d*) δ 152.3, 138.9, 137.3, 125.7, 116.6, 85.7, 82.1, 79.0, 73.7, 73.5, 72.4, 21.4. **m.p.** 105 - 107 °C **$[\alpha]_D^{25°C}$**= +51.0 (c= 0.628 , $CHCl_3$). **Elemental Analysis:** Calcd. for $C_{15}H_{19}NO_5$: C, 61.42; H, 6.53; N, 4.78; O, 27.27. Found C, 61.12; H, 6.68; N, 4.80.

6a: **R_f** 0.09 (CH_2Cl_2:Acetone 9:1). **^{1}H NMR** (500 MHz, Chloroform-*d*) δ 7.00 (s, 2H), 6.75 (bs, 1H), 6.74 – 6.71 (m, 1H), 5.23 – 5.15 (m, 1H), 4.88 (t, *J* = 4.7 Hz, 1H), 4.46 (dd, *J* = 4.3, 1.0 Hz, 1H), 4.40 – 4.34 (m, 1H), 4.03 (dd, *J* = 9.5, 6.4 Hz, 1H), 3.96 (d, *J* = 2.2 Hz, 2H), 3.78 – 3.72 (m, 1H), 2.28 (s, 6H), 1.82 – 1.62 (m, 1H). **^{13}C{^{1}H} NMR** (126 MHz, Chloroform-*d*) δ 152.9, 138.9, 137.4, 125.6, 116.6, 88.1, 80.8, 76.3, 75.8, 74.6, 70.1, 21.4. **m.p.** 104 - 106 °C. **$[\alpha]_D^{25°C}$**= +62.1 (c= 0.620, $CHCl_3$). **Elemental Analysis:** Calcd. for $C_{15}H_{19}NO_5$: C, 61.42; H, 6.53; N, 4.78; O, 27.27. Found C, 61.03; H, 6.80; N, 4.62.

(3R,3aR,6S,6aR)-hexaydrofuro[3,2-b]furan-6-(1-naphthyl)carbamoyl-3-ol (5b) and (3R,3aR,6S,6aR)-hexaydrofuro[3,2-b]furan-3-(1-naphthyl)carbamoyl-6-ol (6b)

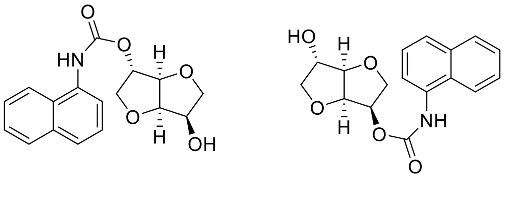

Isosorbide **2** (1.42 g, 9.7 mmol), dry THF (100 mL), 1-naphthylisocyanate **8b** (450 µL, 3.1 mmol), DMAP (25 mg, 0.2 mmol). TLC analysis (CH_2Cl_2: Acetone 9:1). The crude was purified by Biotage® Isolera (CH_2Cl_2:Acetone) to give chemically pure **5b** as a white solid (318 mg, 32%) and chemically pure **6b** as a white foam (271 mg, 27%).

5b: **R_f** 0.24 (CH_2Cl_2:Acetone 9:1). **^{1}H NMR** (500 MHz, DMSO-*d_6*) δ 9.75 (bs, 1H), 8.09 – 8.04 (m, 1H), 7.95 – 7.91 (m, 1H), 7.76 (d, *J* = 8.2 Hz, 1H), 7.60 (d, *J* = 7.5 Hz, 1H), 7.56 – 7.51 (m,

2H), 7.49 (t, *J* = 7.9 Hz, 1H), 5.09 (d, *J* = 3.2 Hz, 1H), 4.95 (d, *J* = 6.4 Hz, 1H), 4.56 (d, *J* = 4.4 Hz, 1H), 4.50 (bs, 1H), 4.21 – 4.14 (m, 1H), 4.03 – 3.94 (m, 2H), 3.78 (dd, *J* = 8.5, 6.5 Hz, 1H), 3.40 (dd, *J* = 8.5, 7.5 Hz, 1H). **$^{13}C\{^1H\}$ NMR** (126 MHz, DMSO-d_6) δ 154.1, 133.7, 133.5, 128.1, 128.0, 126.1, 126.0, 125.9, 125.8, 125.6, 125.2, 122.8, 121.4, 85.3, 81.8, 79.0, 72.8, 72.0, 71.5. **m.p.** 131 - 132 °C. **[α]$_D$$^{25°C}$**= +37.8 (c= 0.558, $CHCl_3$). **Elemental Analysis:** Calcd. for $C_{17}H_{17}NO_5$: C, 64.75; H, 5.43; N, 4.44; O, 25.3. Found C, 64.41; H, 5.58; N, 4.47.

6b: R$_f$ 0.09 (CH_2Cl_2:Acetone 9:1). **^1H NMR** (500 MHz, DMSO-d_6) δ 9.68 (s, 1H), 8.13 – 8.07 (m, 1H), 7.97 – 7.89 (m, 1H), 7.76 (d, *J* = 8.3 Hz, 1H), 7.60 – 7.56 (m, 1H), 7.55 – 7.51 (m, 2H), 7.49 (t, *J* = 7.9 Hz, 1H), 5.21 (d, *J* = 3.9 Hz, 1H), 5.12 (q, *J* = 5.3 Hz, 1H), 4.77 (t, *J* = 5.0 Hz, 1H), 4.30 (d, *J* = 4.6 Hz, 1H), 4.14 (s, 1H), 3.92 – 3.84 (m, 2H), 3.81 – 3.73 (m, 2H). **$^{13}C\{^1H\}$ NMR** (126 MHz, DMSO-d_6) δ 154.4, 133.8, 133.7, 128.2, 128.1, 126.1, 125.8, 125.6, 125.2, 122.9, 121.5, 88.2, 80.6, 75.3, 75.0, 74.5, 69.7. **m.p.** 126 - 129 °C. **[α]$_D$$^{25°C}$**= +44.3 (c= 0.580, $CHCl_3$). **Elemental Analysis:** Calcd. for $C_{17}H_{17}NO_5$: C, 64.75; H, 5.43; N, 4.44; O, 25.3. Found C, 64.31; H, 5.68; N, 4.38.

(3R,3aR,6S,6aR)-hexaydrofuro[3,2-b]furan-6-(3,5-bis(trifluoromethyl)phenyl)carbamoyl-3-ol (5c) and (3R,3aR,6S,6aR)-hexaydrofuro [3,2-b]furan-3-(3,5-bis(trifluoromethyl)phenyl)carbamoyl-6-ol (6c)

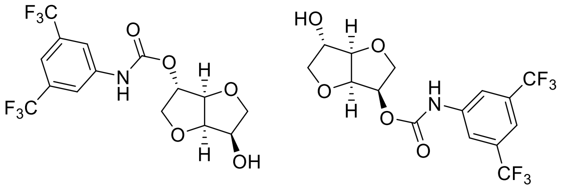

Isosorbide **2** (1.35 g, 9.2 mmol), dry THF (100 mL), 3,5-bis-(trifluoromethyl)phenylisocyanate **8c** (500 µL, 2.9 mmol), DMAP (20 mg, 0.2 mmol). TLC analysis (CH_2Cl_2: Acetone 9:1). The crude was purified by Biotage® Isolera (CH_2Cl_2:Acetone) to give chemically pure **5c** as a white foam (341 mg, 30%) and chemically pure **6c** as a white foam (416 mg, 38%).

5c: R_f 0.29 (CH_2Cl_2:Acetone 9:1). **^{1}H NMR** (500 MHz, Chloroform-*d*) δ 7.88 (s, 2H), 7.60 – 7.57 (m, 1H), 7.01 (s, 1H), 5.30 (d, *J* = 3.1 Hz, 1H), 4.66 (t, *J* = 4.9 Hz, 1H), 4.56 (dt, *J* = 4.2, 0.9 Hz, 1H), 4.35 (q, *J* = 5.7 Hz, 1H), 4.15 (dt, *J* = 10.9, 1.0 Hz, 1H), 4.05 (dd, *J* = 10.9, 3.4 Hz, 1H), 3.92 (dd, *J* = 9.6, 5.9 Hz, 1H), 3.62 (dd, *J* = 9.6, 5.8 Hz, 1H), 2.56 (bs, 1H). **^{19}F NMR** (471 MHz, Methanol-d_4) δ -64.56. **^{13}C{^{1}H} NMR** (126 MHz, Methanol-d_4) δ 154.3, 142.4, 133.3 (q, *J* = 33.2 Hz), 124.7 (q, *J* = 271.8 Hz), 119.1 (bs), 116.7 (h, *J* = 4.0 Hz), 87.0, 83.5, 80.9, 74.3, 73.9, 73.0. **m.p.** 185 - 187 °C **$[\alpha]_D^{25°C}$**= +36.5 (c= 0.543, $CHCl_3$). **Elemental Analysis:** Calcd. for $C_{15}H_{13}F_6NO_5$: C, 44.90; H, 3.27; F, 28.41; N, 3.49; O, 19.94 Found C, 44.71; H, 3.38; N, 3.23.

6c: R_f 0.17 (CH_2Cl_2:Acetone 9:1). **^{1}H NMR** (500 MHz, Chloroform-*d*) δ 7.88 (s, 2H), 7.58 – 7.55 (m, 1H), 7.30 (bs, 1H), 5.22 (q, *J* = 5.5 Hz, 1H), 4.92 (t, *J* = 4.7 Hz, 1H), 4.47 (dt, *J* = 4.3, 0.9 Hz, 1H), 4.41 – 4.38 (m, 1H), 4.03 (dd, *J* = 9.8, 6.2 Hz, 1H), 4.00 – 3.91 (m, 2H), 3.80 (dd, *J* = 9.8, 5.7 Hz, 1H), 1.80 (bs, 1H). **^{19}F NMR** (471 MHz, Methanol-d_4) δ -64.55. **^{13}C{^{1}H} NMR** (126 MHz, Methanol-d_4) δ 154.8, 142.6, 133.3 (q, *J* = 33.2 Hz), 124.8 (q, *J* = 271.8 Hz), 119.1 (bs), 116.5 (h, *J* = 4.0 Hz), 89.7, 82.5, 76.8, 76.5, 76.5, 71.7. **m.p.** 110 - 111 °C. **$[\alpha]_D^{25°C}$**= +48.0 (c= 0.553, $CHCl_3$). **Elemental Analysis:** Calcd. for $C_{15}H_{13}F_6NO_5$: C, 44.90; H, 3.27; F, 28.41; N, 3.49; O, 19.94. Found Found C, 44.68; H, 3.49; N, 3.30.

(3R,3aR,6S,6aR)-hexaydrofuro[3,2-b]furan-6-(3,5-dimethoxyphenyl)carbamoyl-3-ol (5d) and (3R,3aR,6S,6aR)-hexaydrofuro[3,2-b]furan-3-(3,5-dimethoxyphenyl) carbamoyl-6-ol (6d)

Isosorbide **2** (1.33 g, 9.1 mmol), dry THF (100 mL), 3,5-dimethoxyphenylisocyanate **8d** (550 mg, 3.1 mmol), DMAP (27 mg, 0.2 mmol). TLC analysis (CH_2Cl_2: Acetone 9:1). The crude was

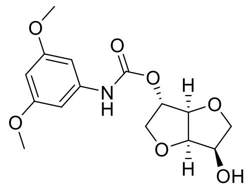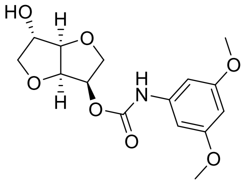

purified by Biotage® Isolera (CH_2Cl_2:Acetone) to give chemically pure **5d** as a white foam (321 mg, 32%) and chemically pure **6d** as a white foam (343 mg, 34%).

5d: R_f 0.15 (CH_2Cl_2:Acetone 9:1). **^{1}H NMR** (500 MHz, Chloroform-*d*) δ 6.66 (bs, 1H), 6.60 (bs, 2H), 6.21 (t, *J* = 2.2 Hz, 1H), 5.26 (d, *J* = 3.2 Hz, 1H), 4.65 (t, *J* = 4.9 Hz, 1H), 4.55 (dt, *J* = 4.4, 1.1 Hz, 1H), 4.33 (q, *J* = 5.8 Hz, 1H), 4.14 (d, *J* = 10.8 Hz, 1H), 4.02 (dd, *J* = 10.7, 3.5 Hz, 1H), 3.91 (dd, *J* = 9.5, 6.0 Hz, 1H), 3.77 (s, 6H), 3.59 (dd, *J* = 9.5, 5.8 Hz, 1H), 2.17 (bs, 1H). **^{13}C{^{1}H} NMR** (126 MHz, Chloroform-*d*) δ 161.2, 152.1, 139.4, 97.1, 96.0, 85.7, 82.1, 79.0, 73.6, 73.5, 72.4, 55.4. **m.p.** 104 - 107 °C **$[\alpha]_D^{25°C}$**= +47.0 (c= 0.573, $CHCl_3$). **Elemental Analysis:** Calcd. for $C_{15}H_{19}NO_7$: C, 55.38; H, 5.89; N, 4.31; O, 34.43 Found C, 55.11; H, 6.04; N, 4.16.

6d: R_f 0.09 (CH_2Cl_2:Acetone 9:1). **^{1}H NMR** (500 MHz, Chloroform-*d*) δ 6.89 (bs, 1H), 6.61 (d, *J* = 2.2 Hz, 2H), 6.20 (t, *J* = 2.2 Hz, 1H), 5.19 (td, *J* = 6.3, 5.1 Hz, 1H), 4.88 (t, *J* = 4.5 Hz, 1H), 4.46 (d, *J* = 4.3 Hz, 1H), 4.38 – 4.35 (m, 1H), 4.03 (dd, *J* = 9.5, 6.3 Hz, 1H), 3.97 – 3.94 (m, 2H), 3.77 (s, 6H), 3.76 – 3.71 (m, 1H), 1.88 (s, 1H). **^{13}C{^{1}H} NMR** (126 MHz, DMSO-*d_6*) δ 160.6, 152.7, 140.9, 96.6, 94.4, 88.2, 80.4, 75.3, 75.0, 74.2, 69.6, 55.1. **m.p.** 126-129 °C. **$[\alpha]_D^{25°C}$**= +77.3 (c= 0.333, $CHCl_3$). **Elemental Analysis:** Calcd. for $C_{15}H_{19}NO_7$: C, 55.38; H, 5.89; N, 4.31; O, 34.43. Found C, 55.01; H, 6.03; N, 4.10.

(3R,3aR,6S,6aR)-hexaydrofuro[3,2-b]furan-6-(p-toluensulfonyl)carbamoyl-3-ol (5e) and (3R,3aR,6S,6aR)-hexaydrofuro[3,2-b]furan-3-(p-toluensulfonyl)carbamoyl-6-ol (6e)

Isosorbide **2** (2.87 g, 19.6 mmol), dry THF (100 mL), p-toluensulfonylisocyanate **8e** (920 μL, 6.0 mmol). TLC analysis ($CHCl_3$:MeOH 97:3, 1% HCOOH). The crude was purified by Flash

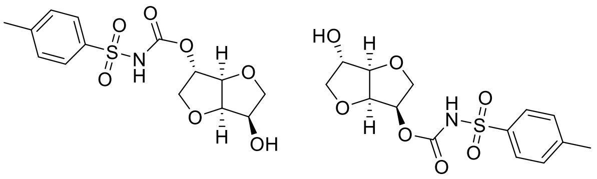

chromatography on silica gel ($CHCl_3$:MeOH 97:3, 1% HCOOH) to give chemically pure **5e** as a white foam (362 mg, 18%) and chemically pure **6e** as a white solid (320 mg, 16%).

5e: R$_f$ 0.14 ($CHCl_3$:MeOH 97:3, 1% HCOOH). **^{1}H NMR** (400 MHz, Chloroform-*d*) δ 7.99 (bs, 1H), 7.94 – 7.88 (m, 2H), 7.39 – 7.33 (m, 2H), 5.16 – 5.14 (m, 1H), 4.57 (t, *J* = 4.9 Hz, 1H), 4.42 (dt, *J* = 4.5, 1.0 Hz, 1H), 4.31 (q, *J* = 5.8 Hz, 1H), 4.03 – 3.90 (m, 2H), 3.87 (dd, *J* = 9.6, 6.0 Hz, 1H), 3.55 (dd, *J* = 9.6, 5.9 Hz, 1H), 2.46 (s, 3H), 1.85 (bs, 1H). **^{13}C{^{1}H} NMR** (126 MHz, Methanol-*d$_4$*) δ 152.0, 146.2, 137.8, 130.6, 129.1, 86.6, 83.4, 81.6, 73.9, 73.7, 73.0, 21.6. **m.p.** 121 - 122 °C. **[α]$_D^{25°C}$**= +41.1 (c= 0.565, $CHCl_3$). **Elemental Analysis:** Calcd. for $C_{14}H_{17}NO_7S$: C, 48.97; H, 4.99; N, 4.08; O, 32.62; S, 9.34. Found C, 48.72; H, 5.13; N, 4.15.

6e: R$_f$ 0.09 ($CHCl_3$:MeOH 97:3, 1% HCOOH). **^{1}H NMR** (400 MHz, Acetone-*d$_6$*) δ 7.92 – 7.87 (m, 2H), 7.47 – 7.41 (m, 2H), 5.05 – 4.98 (m, 1H), 4.70 (t, *J* = 5.2 Hz, 1H), 4.27 (d, *J* = 4.8 Hz, 1H), 4.15 – 4.12 (m, 1H), 3.76 (dd, *J* = 10.1, 5.4 Hz, 1H), 3.69 – 3.63 (m, 3H), 2.89 (bs, 2H), 2.44 (s, 3H). **^{13}C{^{1}H} NMR** (126 MHz, Methanol-*d$_4$*) δ 152.5, 146.0, 137.9, 130.6, 129.1, 89.7, 82.1, 77.4, 76.5, 76.2, 71.5, 21.5. **m.p.** 152-155 °C. **[α]$_D^{25°C}$**= +77.5 (c= 0.520, Acetone). **Elemental Analysis:** Calcd. for $C_{14}H_{17}NO_7S$: C, 48.97; H, 4.99; N, 4.08; O, 32.62; S, 9.34 Found C, 48.67; H, 5.08; N, 4.11.

*General procedure for the synthesis of compounds **4** and **7***

Under an Ar atmosphere, phenyl isocyanate **8** (1.2 equivs.) and 4-(dimethylamino)pyridine (DMAP) (0.12 equivs.) were added to a solution of isohexide **1** or **2** (1 equiv.) in dry THF (4 mL/equiv. of **1** or **2**). The reaction was monitored by TLC analysis. After 18 h the solvent was removed under reduced pressure, the crude was dissolved in CH_2Cl_2 (20 mL) and washed with water (3x10 mL). The organic phase was dried over anhydrous Na_2SO_4 and the

solvent was removed under pressure to give the crude. The crude was processed as described in the following sections.

(3R,3aR,6R,6aR)-hexaydrofuro[3,2-b]furan-3,6-di-(3,5-dimethylphenyl)carbamate (4a)

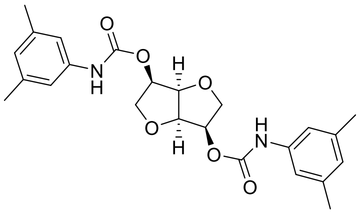

Isomannide **1** (434 mg, 3.0 mmol), dry THF (10 mL), 3,5-dimethylphenylisocyanate **8a** (1.0 mL, 7.2 mmol), DMAP (41 mg, 0.3 mmol). TLC analysis (CH_2Cl_2: Acetone 9:1). The crude was recrystallized from CH_2Cl_2:Hexane to give the chemically pure product **4a** as a white solid (773 mg, 60%).

R_f 0.84 (CH_2Cl_2:Acetone 9:1). **^{1}H NMR** (500 MHz, Chloroform-*d*) δ 6.99 (s, 4H), 6.79 (bs, 2H), 6.72 (s, 2H), 5.24 – 5.17 (m, 2H), 4.79 – 4.75 (m, 2H), 4.15 (dd, *J* = 9.2, 6.4 Hz, 2H), 3.89 (t, *J* = 8.0 Hz, 2H), 2.28 (s, 12H). **^{13}C{^{1}H} NMR** (126 MHz, Chloroform-*d*) δ 152.7, 138.9, 138.9, 137.5, 125.5, 116.5, 81.1, 74.3, 70.7, 21.4. **m.p.** 201 - 203 °C **$[\alpha]_D^{25°C}$**= +154.1 (c= 0.495, $CHCl_3$). **Elemental Analysis:** Calcd. for $C_{24}H_{28}N_2O_6$: C, 65.44; H, 6.41; N, 6.36; O, 21.79. Found C, 65.13; H, 6.60; N, 6.16.

(3R,3aR,6S,6aR)-hexaydrofuro[3,2-b]furan-3,6-di-(3,5-dimethylphenyl)carbamate (7a)

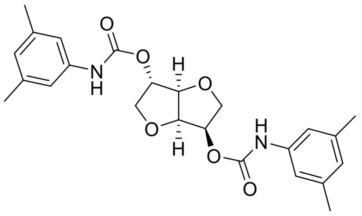

Isosorbide **2** (419 mg, 2.9 mmol), dry THF (10 mL), 3,5-dimethylphenylisocyanate **8a** (1.0 mL, 7.2 mmol), DMAP (35 mg, 0.3 mmol). TLC analysis (CH_2Cl_2: Acetone 9:1). The crude was recrystallized from CH_2Cl_2:Hexane to give the chemically pure product **4a** as a white solid (734 mg, 58%).

R_f 0.80 (CH_2Cl_2:Acetone 9:1). **^{1}H NMR** (500 MHz, Chloroform-*d*) δ 7.00 (s, 4H), 6.73 (s, 3H), 6.64 (s, 1H), 5.25 (d, *J* = 3.2 Hz, 1H), 5.21 (td, *J* = 6.2, 5.1 Hz, 1H), 4.88 (t, *J* = 4.8 Hz, 1H), 4.60

(d, J = 4.4 Hz, 1H), 4.12 – 4.08 (m, 2H), 4.05 (dd, J = 9.6, 6.2 Hz, 1H), 3.82 (dd, J = 9.6, 6.1 Hz, 1H), 2.28 (s, 12H). **^{13}C{^{1}H} NMR** (126 MHz, Chloroform-*d*) δ 152.7, 152.5, 138.9, 137.4, 137.4, 125.6, 125.5, 116.6, 85.9, 81.3, 78.7, 74.5, 73.7, 70.3, 21.4. **m.p.** 169 - 170 °C. **[α]$_D$$^{25°C}$**= +54.1 (c= 0.533, $CHCl_3$). **Elemental Analysis:** Calcd. for $C_{24}H_{28}N_2O_6$: C, 65.44; H, 6.41; N, 6.36; O, 21.79. Found C, 65.19; H, 6.52; N, 6.21.

(3R,3aR,6R,6aR)-hexaydrofuro[3,2-b]furan-3,6-di-(1-naphthyl)carbamate (4b)

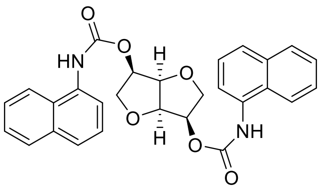

Isomannide **1** (440 mg, 3.0 mmol), dry THF (30 mL), 1-naphthylisocyanate **8b** (1.1 mL, 7.7 mmol), DMAP (52 mg, 0.4 mmol). TLC analysis (CH_2Cl_2:Acetone 9:1). The mixture was filtered to give chemically pure **4b** as a white solid (882 mg, 60%) and the crude, treated as described in the general section, was recrystallized from CH_2Cl_2:Hexane to give the chemically pure product **4b** as a white solid (349 mg, 24%). The total yield was 84%.

R$_f$ 0.48 (CH_2Cl_2:Acetone 9:1). **^{1}H NMR** (500 MHz, DMSO-d_6) δ 9.74 (s, 2H), 8.14 – 8.08 (m, 2H), 7.96 – 7.91 (m, 2H), 7.78 (d, J = 8.3 Hz, 2H), 7.60 (d, J = 7.4 Hz, 2H), 7.58 – 7.48 (m, 6H), 5.15 – 5.08 (m, 2H), 4.76 – 4.71 (m, 2H), 4.07 (t, J = 7.8 Hz, 2H), 3.90 (bs, 2H). **^{13}C{^{1}H} NMR** (126 MHz, DMSO-d_6) δ 154.3, 133.8, 133.6, 128.2, 128.1, 126.01 125.9, 125.6, 125.3, 122.9, 121.6, 80.5, 74.1, 69.9. **m.p.** 256- 259 °C. **[α]$_D$$^{25°C}$**= +119.5 (c= 0.553, DMSO). **Elemental Analysis:** Calcd. for $C_{28}H_{24}N_2O_6$: C, 69.41; H, 4.99; N, 5.78; O, 19.81. Found C, 69.15; H, 5.16; N, 5.59.

(3R,3aR,6S,6aR)-hexaydrofuro[3,2-b]furan-3,6-di-(1-naphthyl)carbamate (7b)

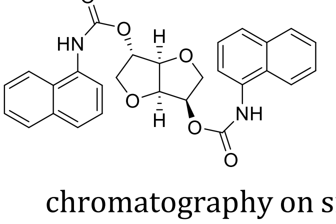

Isosorbide **2** (455 mg, 3.1 mmol), dry THF (30 mL), 1-naphthylisocyanate **8b** (1.1 mL, 7.7 mmol), DMAP (44 mg, 0.4 mmol). TLC analysis (CH_2Cl_2: Acetone 9:1). The crude was purified by Flash chromatography on silica gel (CH_2Cl_2:Acetone 97:3, 1% HCOOH) to give the chemically pure product **7b** as a white solid (1.02 g, 67%).

R_f 0.27 (CH_2Cl_2:Acetone 97:3, 1% HCOOH). **^{1}H NMR** (500 MHz, DMSO-d_6) δ 9.79 (d, J = 29.4 Hz, 2H), 8.16 – 8.11 (m, 1H), 8.11 – 8.07 (m, 1H), 7.95 – 7.91 (m, 2H), 7.77 (d, J = 8.1 Hz, 2H), 7.62 (t, J = 7.4 Hz, 2H), 7.58 – 7.48 (m, 6H), 5.23 (q, J = 4.9 Hz, 1H), 5.20 (bs, 1H), 4.93 (bs, 1H), 4.61 (d, J = 5.0 Hz, 1H), 4.20 – 4.06 (m, 2H), 3.91 (d, J = 4.6 Hz, 2H). **^{13}C{^{1}H} NMR** (126 MHz, DMSO-d_6) δ 154.3, 154.1, 133.8, 133.7, 133.5, 128.1, 128.1, 128.0, 126.1, 125.9, 125.9, 125.6, 125.3, 125.2, 122.9, 122.8, 121.5, 85.8, 81.2, 78.3, 74.2, 72.9, 70.5. **m.p.** 208-210 °C. **$[\alpha]_D^{25°C}$**= +21.1 (c= 0.570, DMSO). **Elemental Analysis:** Calcd. for $C_{28}H_{24}N_2O_6$: C, 69.41; H, 4.99; N, 5.78; O, 19.81. Found C, 69.07; H, 5.29; N, 5.50.

(3R,3aR,6R,6aR)-hexaydrofuro[3,2-b]furan-3,6-di-(3,5-bis(trifluoromethyl)phenyl) carbamate (4c)

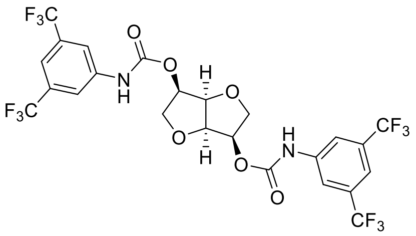

Isomannide **1** (400 mg, 2.7 mmol), dry THF (20 mL), 3,5-bis(trifluoromethyl)phenylisocyanate **8c** (1.1 mL, 6.6 mmol), DMAP (50 mg, 0.4 mmol). TLC analysis (CH_2Cl_2: Acetone 9:1). The crude was recrystallized from CH_2Cl_2:Hexane to give the chemically pure product **4c** as a white solid (991 mg, 55%).

R_f 0.39 (CH_2Cl_2:Acetone 9:1). **^{1}H NMR** (500 MHz, Chloroform-d) δ 7.88 (s, 4H), 7.58 – 7.57 (m, 2H), 7.20 (bs, 2H), 5.27 – 5.20 (m, 2H), 4.86 – 4.82 (m, 2H), 4.17 (dd, J = 9.4, 6.5 Hz, 2H), 3.90 (dd, J = 7.0, 2.6 Hz, 2H). **^{19}F NMR** (471 MHz, Methanol-$d4$) δ -64.56. **^{13}C{^{1}H} NMR** (126 MHz, Methanol-d_4) δ 154.65, 142.46, 133.32 (q, J = 33.2 Hz), 124.74 (q, J = 271.8 Hz), 119.13 (bs), 116.63 (h, J = 3.7 Hz), 82.25, 76.04, 71.46. **m.p.** 156-160 °C. **$[\alpha]_D^{25°C}$**= +119.5 (c= 0.585, $CHCl_3$). **Elemental Analysis:** Calcd. for $C_{24}H_{16}F_{12}N_2O_6$: C, 43.92; H, 2.46; F, 34.73; N, 4.27; O, 14.62. Found C, 43.61; H, 2.69; N, 4.16.

(3R,3aR,6S,6aR)-hexaydrofuro[3,2-b]furan-3,6-di-(3,5-bis(trifluoromethyl)phenyl) carbamate (7c)

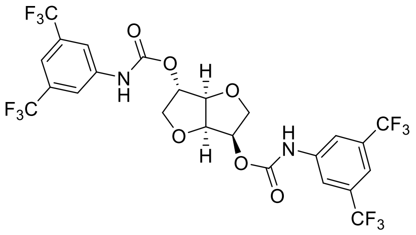

Isosorbide **2** (398 mg, 2.7 mmol), dry THF (20 mL), 3,5-bis(trifluoromethyl)phenylisocyanate **8c** (1.1 mL, 6.6 mmol), DMAP (44 mg, 0.4 mmol). TLC analysis (CH_2Cl_2: Acetone 9:1). The crude was recrystallized from CH_2Cl_2:Hexane to give the chemically pure product **4c** as a white solid (983 mg, 55%).

R_f 0.52 (CH_2Cl_2:Acetone 9:1). **^{1}H NMR** (500 MHz, Chloroform-*d*) δ 7.89 – 7.86 (m, 4H), 7.58 – 7.56 (m, 2H), 7.21 (s, 1H), 7.16 (s, 1H), 5.31 (d, *J* = 3.0 Hz, 1H), 5.26 (q, *J* = 5.5 Hz, 1H), 4.96 (t, *J* = 5.0 Hz, 1H), 4.63 (d, *J* = 4.6 Hz, 1H), 4.13 (d, *J* = 10.8 Hz, 1H), 4.09 – 4.02 (m, 2H), 3.91 (dd, *J* = 10.0, 5.3 Hz, 1H). **^{19}F NMR** (471 MHz, Chloroform-*d*) δ -63.03. **^{13}C{^{1}H} NMR** (126 MHz, Chloroform-*d*) δ 152.3, 151.9, 139.1, 139.0,132.7 (qd, *J* = 33.5, 7.1 Hz), 123.1 (q, *J* = 272.8 Hz), 118.4 (bs), 117.5 – 117.1(m), 86.01, 81.24, 79.31, 75.25, 73.53, 70.60. **m.p.** 91-95 °C. **$[\alpha]_D^{25°C}$**= +39.5 (c= 0.565, $CHCl_3$). **Elemental Analysis:** Calcd. for $C_{24}H_{16}F_{12}N_2O_6$: C, 43.92; H, 2.46; F, 34.73; N, 4.27; O, 14.62. Found C, 43.52; H, 2.53; N, 4.20.

(3R,3aR,6R,6aR)-hexaydrofuro[3,2-b]furan-3,6-di-(3,5-dimethoxyphenyl)carbamate (4d)

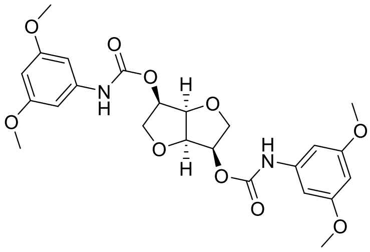

Isomannide **1** (449 mg, 3.1 mmol), dry THF (20 mL), 3,5-dimethoxyphenylisocyanate **8d** (1.32 g, 7.4 mmol), DMAP (49 mg, 0.4 mmol). The mixture was filetered to give chemically pure **4d** as a white solid (872 mg, 56%). The crude was treated as described in the general section and it was recrystallized from Acetone:Hexane to give the chemically pure product **4d** as a white solid (298 mg, 19%). The total yield was 75%.

R$_f$ 0.43 (CH_2Cl_2:Acetone 9:1). **^{1}H NMR** (500 MHz, Chloroform-*d*) δ 6.83 (bs, 2H), 6.60 (d, *J* = 2.2 Hz, 4H), 6.20 (t, *J* = 2.2 Hz, 2H), 5.19 (tdd, *J* = 6.7, 3.8, 1.6 Hz, 2H), 4.79 – 4.75 (m, 2H), 4.18 – 4.12 (m, 2H), 3.92 – 3.85 (m, 2H), 3.77 (s, 12H). **^{13}C{^{1}H} NMR** (126 MHz, DMSO-d_6) δ 160.6, 152.7, 140.7, 96.7, 94.5, 80.3, 73.9, 69.8, 55.1. **m.p.** 202-204 °C. **$[\alpha]_D^{25°C}$**= +145.4 (c= 0.630, $CHCl_3$). **Elemental Analysis:** Calcd. for $C_{24}H_{28}N_2O_{10}$: C, 57.14; H, 5.59; N, 5.55; O, 31.71. Found C, 56.93; H, 5.91; N, 5.63.

(3R,3aR,6S,6aR)-hexaydrofuro[3,2-b]furan-3,6-di-(3,5-dimethoxyphenyl)carbamate (7d)

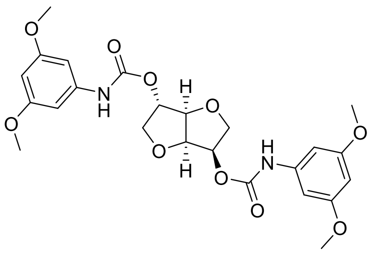

Isosorbide **2** (442 mg, 3.0 mmol), dry THF (20 mL), 3,5-dimethoxyphenylisocyanate **8d** (1.32 g, 7.3 mmol), DMAP (57 mg, 0.5 mmol). TLC analysis (CH_2Cl_2:Acetone 9:1). The crude was purified by Biotage® Isolera ($CHCl_3$:Acetone) to give the chemically pure product **3d** as a white solid (827 mg, 54%).

R$_f$ 0.33 ($CHCl_3$:Acetone 9:1). **^{1}H NMR** (500 MHz, Chloroform-*d*) δ 6.89 – 6.84 (m, 2H), 6.61 (bs, 2H), 6.59 (d, *J* = 2.2 Hz, 2H), 6.20 (q, *J* = 2.3 Hz, 2H), 5.26 (d, *J* = 3.3 Hz, 1H), 5.24 – 5.19 (m, 1H), 4.87 (t, *J* = 4.8 Hz, 1H), 4.60 (d, *J* = 4.4 Hz, 1H), 4.12 – 4.03 (m, 3H), 3.83 – 3.78 (m, 1H), 3.76 (s, 12H). **^{13}C{^{1}H} NMR** (126 MHz, DMSO-d_6) δ 160.6, 152.6, 152.4, 140.8, 140.6, 96.8, 96.6, 94.5, 94.4, 85.7, 81.0, 77.9, 73.8, 72.7, 70.4, 55.1. **m.p.** 165 - 167 °C. **$[\alpha]_D^{25°C}$**= +49.8 (c= 0.458, $CHCl_3$). **Elemental Analysis:** Calcd. for $C_{24}H_{28}N_2O_{10}$: C, 57.14; H, 5.59; N, 5.55; O, 31.71. Found Found C, 57.01; H, 5.73; N, 5.60.

(3R,3aR,6R,6aR)-hexaydrofuro[3,2-b]furan-3,6-di-(p-toluensulfonyl)carbamate (4e)

Isomannide **1** (456 mg, 3.1 mmol), dry THF (20 mL), p-toluensulfonylisocyanate **8e** (1.1 mL, 7.2 mmol), DMAP (47 mg, 0.4 mmol). TLC analysis ($CHCl_3$:Acetone 95:5, 1% HCOOH). The

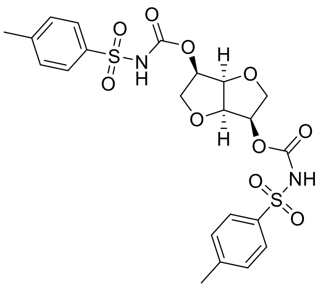

crude was purified by Flash chromatography on silica gel ($CHCl_3$:Acetone 95:5, 1% HCOOH) to give the chemically pure product **4e** as a white foam (1.03 g, 61%).

R$_f$ 0.15 ($CHCl_3$:Acetone 95:5, 1% HCOOH). **^{1}H NMR** (500 MHz, Chloroform-*d*) δ 8.58 (s, 2H), 7.96 – 7.89 (m, 4H), 7.37 – 7.31 (m, 4H), 5.00 – 4.94 (m, 2H), 4.62 – 4.59 (m, 2H), 3.89 (dd, *J* = 9.9, 5.9 Hz, 2H), 3.72 (dd, *J* = 10.0, 5.8 Hz, 2H), 2.43 (s, 6H). **^{13}C{^{1}H} NMR** (126 MHz, Methanol-d_4) δ 153.0, 145.8, 138.2, 130.5, 129.0, 81.7, 76.4, 71.2, 21.5. **m.p.** 105-110 °C. **[α]$_D^{25°C}$**= +85.2 (c= 0.518, $CHCl_3$). **Elemental Analysis:** Calcd. for $C_{22}H_{24}N_2O_{10}S_2$: C, 48.88; H, 4.48; N, 5.18; O, 29.60; S, 11.86. Found C, 48.45; H, 4.79; N, 5.27.

(3R,3aR,6S,6aR)-hexaydrofuro[3,2-b]furan-3,6-di-(p-toluensulfonyl)carbamoyl-3-ol (7e)

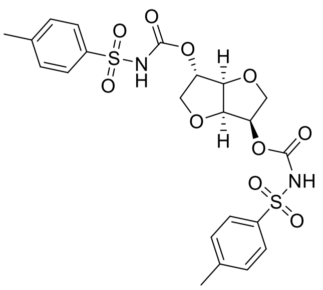

Isosorbide **2** (465 mg, 3.2 mmol), dry THF (20 mL), p-toluensulfonylisocyanate **8e** (1.1 mL, 7.2 mmol), DMAP (47 mg, 0.4 mmol). TLC analysis ($CHCl_3$:Acetone 95:5, 1% HCOOH). The crude was purified by Flash chromatography on silica gel ($CHCl_3$:Acetone 95:5, 1% HCOOH) to give the chemically pure product **4e** as a white foam (1.38 g, 80%).

R$_f$ 0.17 ($CHCl_3$:Acetone 95:5, 1% HCOOH). **^{1}H NMR** (500 MHz, Chloroform-*d*) δ 8.32 (s, 1H), 8.28 (s, 1H), 7.92 – 7.87 (m, 4H), 7.36 – 7.31 (m, 4H), 5.06 (d, *J* = 3.2 Hz, 1H), 5.05 – 5.00 (m, 1H), 4.71 (t, *J* = 5.1 Hz, 1H), 4.35 (d, *J* = 4.6 Hz, 1H), 3.89 – 3.78 (m, 2H), 3.76 – 3.68 (m, 2H), 2.44 (d, *J* = 5.0 Hz, 6H). **^{13}C{^{1}H} NMR** (126 MHz, Methanol-d_4) δ 152.3, 152.0, 146.2, 146.1, 137.8, 137.7, 130.6, 130.6, 129.1, 129.1, 86.9, 82.4, 80.6, 77.1, 73.5, 71.9, 21.6. **m.p.** 99-103 °C. **[α]$_D^{25°C}$**= +46.8 (c= 0.500, $CHCl_3$). **Elemental Analysis:** Calcd. for $C_{22}H_{24}N_2O_{10}S_2$: C, 48.88; H, 4.48; N, 5.18; O, 29.60; S, 11.86. Found C, 48.50; H, 4.64; N, 5.09.

*Synthesis of phenylglycine methylesters (compounds **9** and **10**)*

N-3,5-dinitrobenzoylphenylglycine (9’)

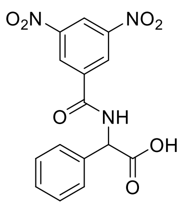

Under an Ar atmosphere, to a dispersion of α-phenylglycine in dry THF, *rac*-propylene oxide and 3,5-dinitrobezoyl chloride were added. The mixture was stirred at room temperature for 1h. While the reaction proceeded, the disperded solids started to solubilize. After 1h the solvent was removed under reduced pressure to give the crude product as a beige solid, which was purified by crystallization from boiling acetonitrile to give the pure product as a white solid.

rac-N-3,5-dinitrobenzoylphenylglycine, **9’:** *rac*-α-phenylglycine (1.52 g, 10.0 mmol), 30mL of dry THF, *rac*-propylene oxide (2.1 mL, 30.0 mmol) and 3,5-dinitrobezoyl chloride (2.30 g, 10.0 mmol). White solid (2.34 g, 68%).

(R)-(-)-N-3,5-dinitrobenzoylphenylglycine, *(R)*-**9’:** *R-(-)*-α-phenylglycine (2.91 g, 19.3 mmol), 60 mL of dry THF, *rac*-propylene oxide (4.2 mL, 59.9 mmol) and 3,5-dinitrobezoyl chloride (4.65 g, 20.1 mmol). White solid (4.54 g, 68%).

(S)-(+)-N-3,5-dinitrobenzoylphenylglycine, (S)-**9’:** (*S)-(+)*-α-phenylglycine (3.00 g, 10.0 mmol), 60 mL of dry THF, *rac*-propylene oxide (4.2 mL, 59.9 mmol) and 3,5-dinitrobezoyl chloride (4.68 g, 20.3 mmol). White solid (4.91 g, 72%).

^{1}H NMR (401 MHz, DMSO-*d*$_6$) δ 13.10 (bs, 1H), 9.88 (d, *J* = 7.0 Hz, 1H), 9.13 (d, *J* = 2.1 Hz, 2H), 8.97 (t, *J* = 2.1 Hz, 1H), 7.56 – 7.46 (m, 2H), 7.46 – 7.32 (m, 3H), 5.64 (d, *J* = 7.0 Hz, 1H).

N-3,5-dinitrobenzoylphenylglycine methyl ester (9)

To a dispersion of 3,5-dintrobenzoylphenylglycine **9’** in dry THF, 1,8-Diazabicyclo[5.4.0]undec-7-ene (DBU) was slowly added and the dispersion instantaneously changed its color into purple. The mixture was stirred for 10 minutes and

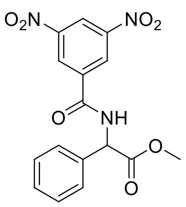

then methyl iodide was slowly added. The reaction was stirred at room temperature, it was monitored by TLC analysis (Hexane:Acetone 7:3) and it was stopped after 21 h. The solvent was removed under pressure and the crude yellow solid was dissolved in CH_2Cl_2; the organic phase was washed with 10% $NaHCO_{3(aq)}$, saturated $Na_2S_2O_{3(aq)}$ and water until neutrality. The organic phase was dried over anhydrous Na_2SO_4 and the solvent was removed under reduced pressure to give the crude product as a white-pink solid. The crude was purified by recristallyzation from CH_2Cl_2/Hexane to give the pure product as a white solid.

rac-N-3,5-dinitrobenzoylphenylglycine methyl ester **9:**

rac-N-3,5-dintrobenzoylphenylglycine **9** (994 mg, 2.8 mmol), 6 mL of dry THF, 1,8-Diazabicyclo[5.4.0]undec-7-ene (DBU) and methyl iodide (1.2 mL, 19.3 mmol). White solid (729 mg, 73%).

(S)-(+)-N-3,5-dinitrobenzoylphenylglycine methyl ester, *(S)*-**9:**

(S)-(+)-N-3,5-dintrobenzoylphenylglycine *(S)*-**9'** (694 mg, 2.0 mmol), 5 mL of anhydrous THF, 1,8-Diazabicyclo[5.4.0]undec-7-ene (DBU, 300 µL, 2.0 mmol) and methyl iodide (800 µL, 12.9 mmol). White solid (499 mg, 69%). $[\alpha]_D^{26°C}$= +107.5 (c= 0.802, THF) (lit. found $[\alpha]_D^{20°C}$= +101.0 (c= 1, THF)).[X]

(R)-(-)-N-3,5-dinitrobenzoylphenylglycine methyl ester, (R)-**9:**

(*R*)-(-)-N-3,5-dintrobenzoylphenylglycine *(R)*-**9'** (688 mg, 2.0 mmol), 5 mL of anhydrous THF, 1,8-Diazabicyclo[5.4.0]undec-7-ene (DBU, 300 µL, 2.0 mmol) and methyl iodide (800 µL, 12.9 mmol). White solid (458 mg, 64%). $[\alpha]_D^{26°C}$= -107.0 (c= 0.800, THF) (lit. found $[\alpha]_D^{20°C}$= -101.0 (c= 1, THF)).[X]

^{1}**H NMR** (500 MHz, Chloroform-*d*) δ 9.18 (t, *J* = 2.1 Hz, 1H), 8.97 (d, *J* = 2.1 Hz, 2H), 7.47 – 7.36 (m, 6H), 5.78 (d, *J* = 6.8 Hz, 1H), 3.81 (s, 3H).

N-3,5-dimethoxybenzoylphenylglycine (10') [2]

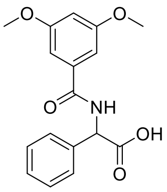

Under an Ar atmosphere, to a dispersion of α-phenylglycine (462 mg, 3.1 mmol) in dry THF (15 mL), *rac*-propylene oxide (630 µL, 9.0 mmol) and 3,5-dimethoxybezoyl chloride (606 mg, 3.0 mmol) were added. The mixture was stirred overnight at room temperature. After 16 h, the solid was filtered off and the solvent was removed under reduced pressure. The crude product was purified by recrystallization from CH_2Cl_2/Hexane to give the pure product as a white solid (809 mg, 84%).

m.p. 154-155°C. ^{1}**H NMR** (500 MHz, Chloroform-*d*) δ 7.51 – 7.31 (m, 5H), 7.05 (d, J = 6.8 Hz, 1H), 6.91 (d, J = 2.3 Hz, 2H), 6.58 (t, J = 2.3 Hz, 1H), 5.75 (d, J = 6.7 Hz, 1H), 3.79 (s, 6H). ^{13}**C{^{1}H} NMR** (126 MHz, Chloroform-*d*) δ 174.1, 167.0, 161.0, 135.8, 135.6, 129.3, 129.1, 127.6, 105.2, 104.2, 57.0, 55.8.

N-3,5-dimethoxybenzoylphenylglycine methyl ester (10)[3]

To a solution of 3,5-dimethoxybenzoylphenylglycine **10'** (1.58 g, 5.0 mmol) in dry THF (10mL), 750 µL (5.0 mmol) of 1,8-Diazabicyclo[5.4.0]undec-7-ene (DBU) were slowly added. The mixture was stirred for 15 minutes and then 1.8 mL (28.9 mmol) of methyl iodide were slowly added. The reaction was stirred at room temperature, it was monitored by TLC analysis (Hexane:Acetone 7:3) and it was stopped after 16 h. The solvent was removed under pressure and the crude was dissolved in $CHCl_3$ (25 mL); the organic phase was washed with 10% $NaHCO_{3(aq)}$ (25 mL), saturated $Na_2S_2O_{3(aq)}$ (25 mL) and water (25 mL) until neutrality. The organic phase was dried over anhydrous Na_2SO_4 and the solvent was removed under reduced pressure to give the crude product. The crude was purified by

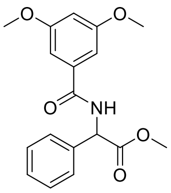

recristallyzation from CH_2Cl_2/Hexane to give the pure product as a white solid (989 mg, 60 %).

R$_f$: 0.23 (Hexane:Acetone 7:3) **m.p.** 103-105°C **^{1}H NMR** (500 MHz, Chloroform-d) δ 7.44 – 7.32 (m, 5H), 7.09 (d, J = 7.1 Hz, 1H), 6.93 (d, J = 2.3 Hz, 2H), 6.58 (t, J = 2.3 Hz, 1H), 5.74 (d, J = 7.0 Hz, 1H), 3.81 (s, 7H), 3.77 (s, 3H). **^{13}C{^{1}H} NMR** (101 MHz, Chloroform-d) δ 171.6, 166.5, 161.1, 136.6, 135.9, 129.2, 128.8, 127.5, 105.2, 104.1, 57.0, 55.8, 53.1.

*^{1}H, ^{13}C and ^{19}F NMR spectra of compounds **3-7** and of compounds **9-10***

(3R,3aR,6R,6aR)-hexaydrofuro[3,2-b]furan-6-(3,5-dimethylphenyl)carbamoyl-3-ol (3a)

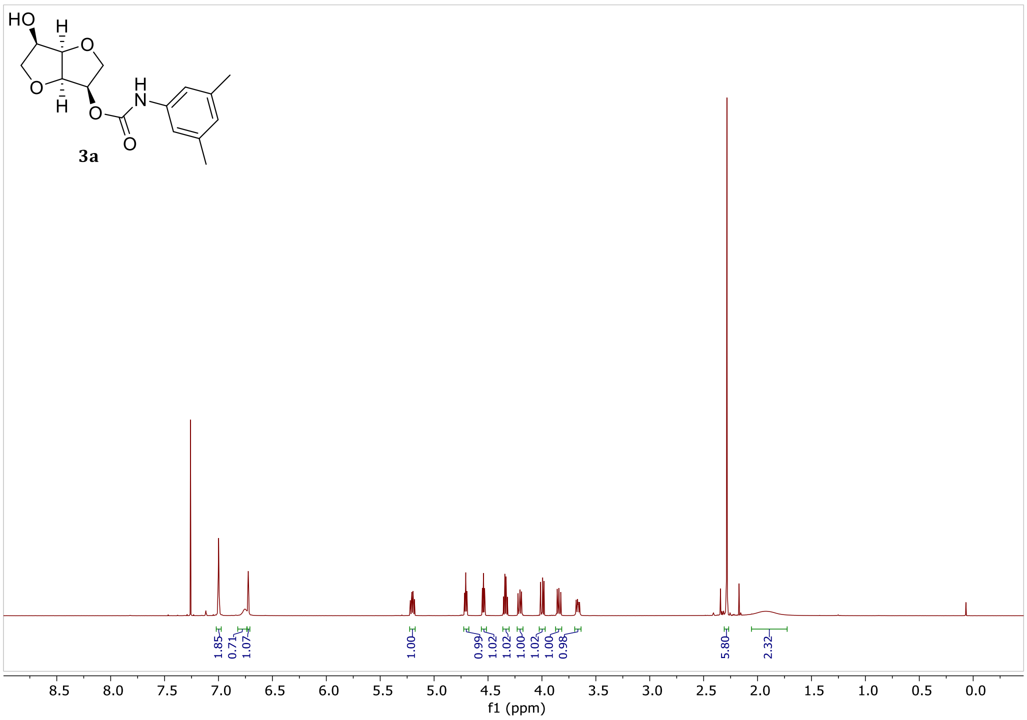

Figure S1. ^{1}H NMR (500 MHz, Chloroform-*d*) spectrum of compound **3a**

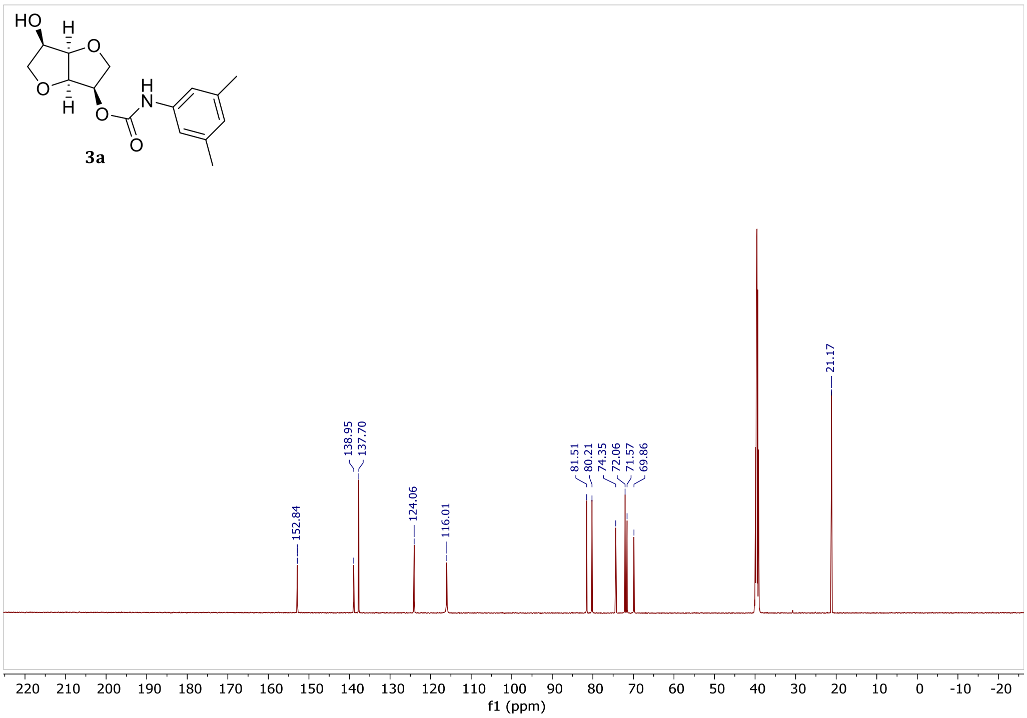

Figure S2. $^{13}C\{^{1}H\}$ NMR (126 MHz, DMSO-d_6) spectrum of compound **3a**

(3R,3aR,6S,6aR)-hexaydrofuro[3,2-b]furan-6-(3,5-dimethylphenyl)carbamoyl-3-ol (5a)

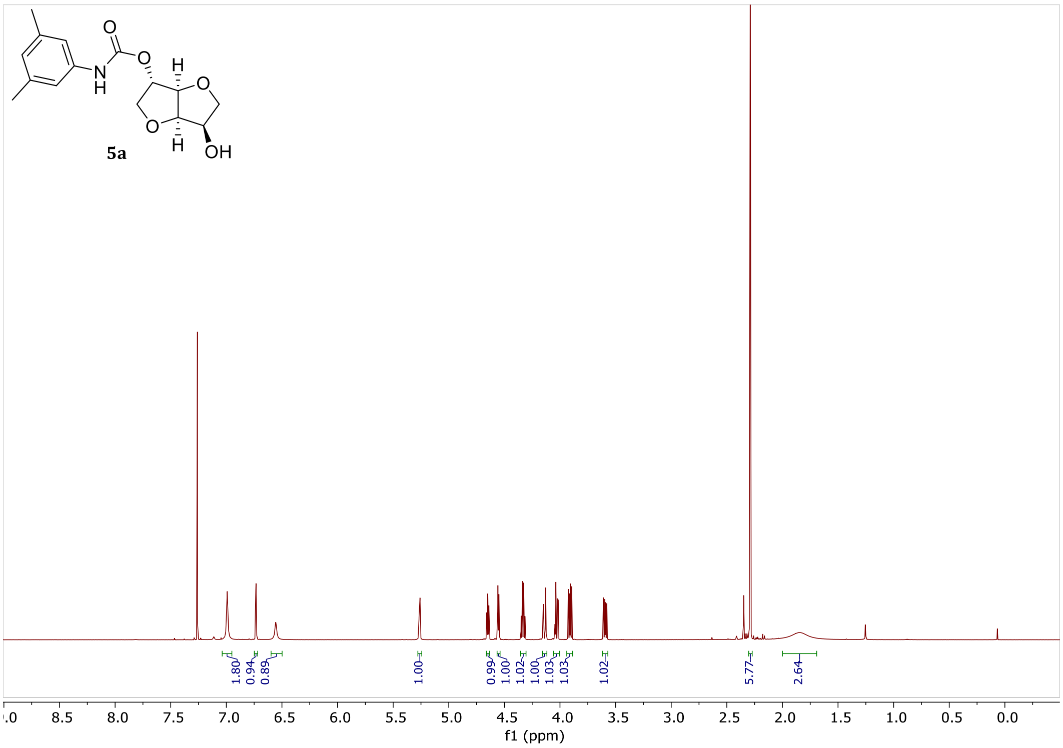

Figure S3. ^{1}H NMR (500 MHz, Chloroform-*d*) spectrum of compound **5a**

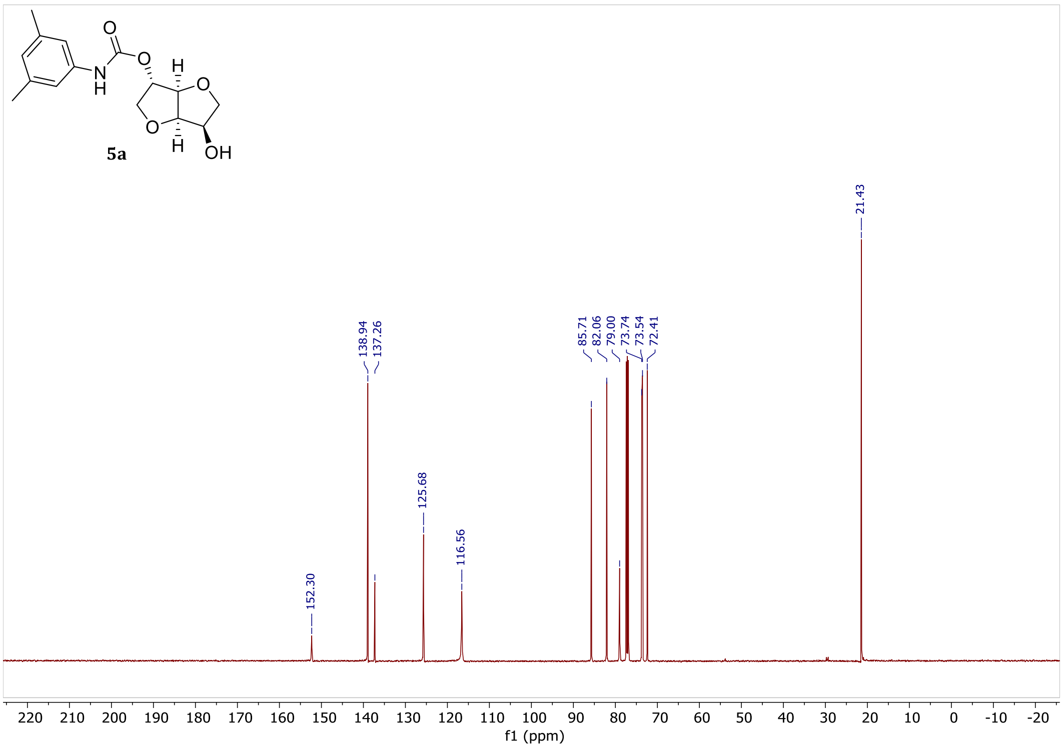

Figure S4. $^{13}C\{^{1}H\}$ NMR (126 MHz, Chloroform-*d*) spectrum of compound **5a**

(3R,3aR,6S,6aR)-hexaydrofuro[3,2-b]furan-3-(3,5-dimethylphenyl)carbamoyl-6-ol (6a)

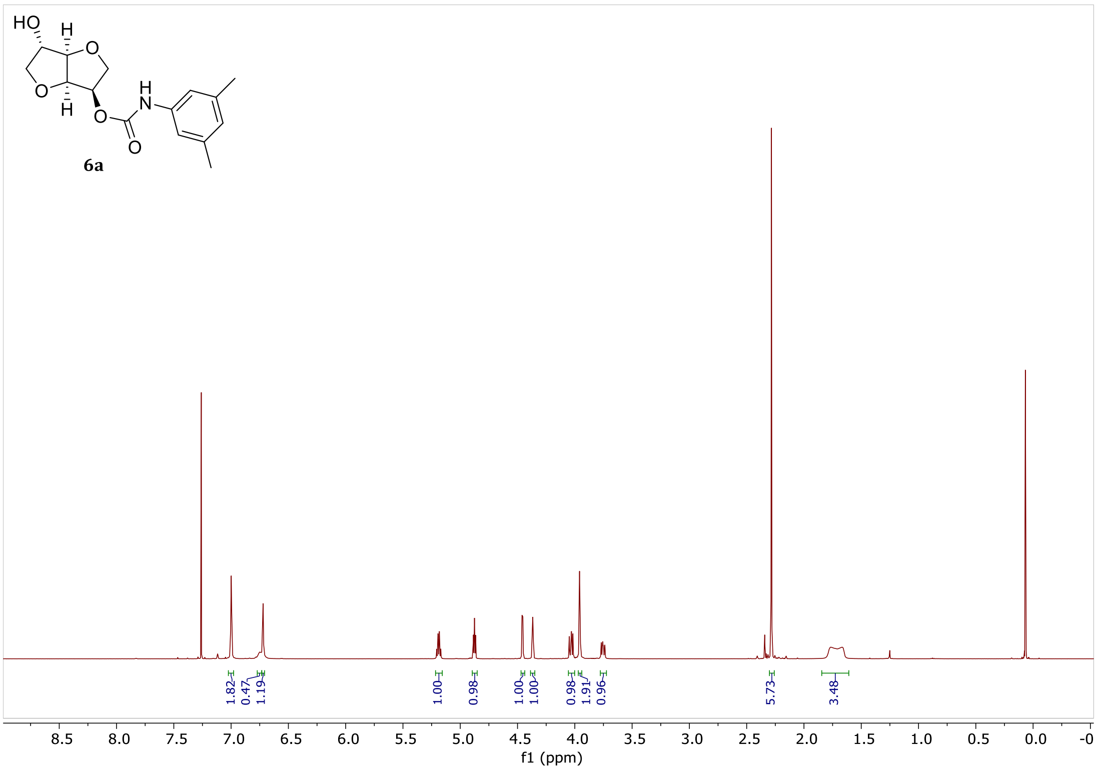

Figure S 5. ^{1}H NMR (500 MHz, Chloroform-*d*) spectrum of compound **6a**

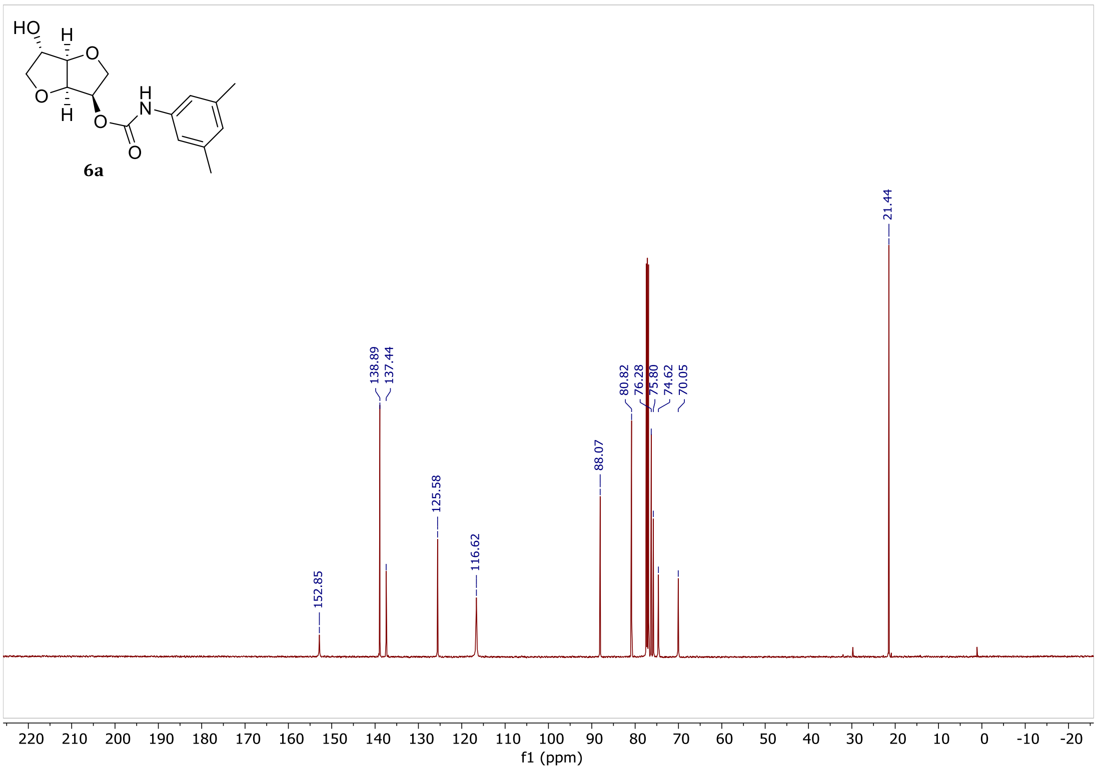

Figure S 6. ^{13}C{^{1}H} NMR (126 MHz, Chloroform-*d*) spectrum of compound **6a**

(3R,3aR,6R,6aR)-hexaydrofuro[3,2-b]furan-6-(1-naphthyl)carbamoyl-3-ol (3b)

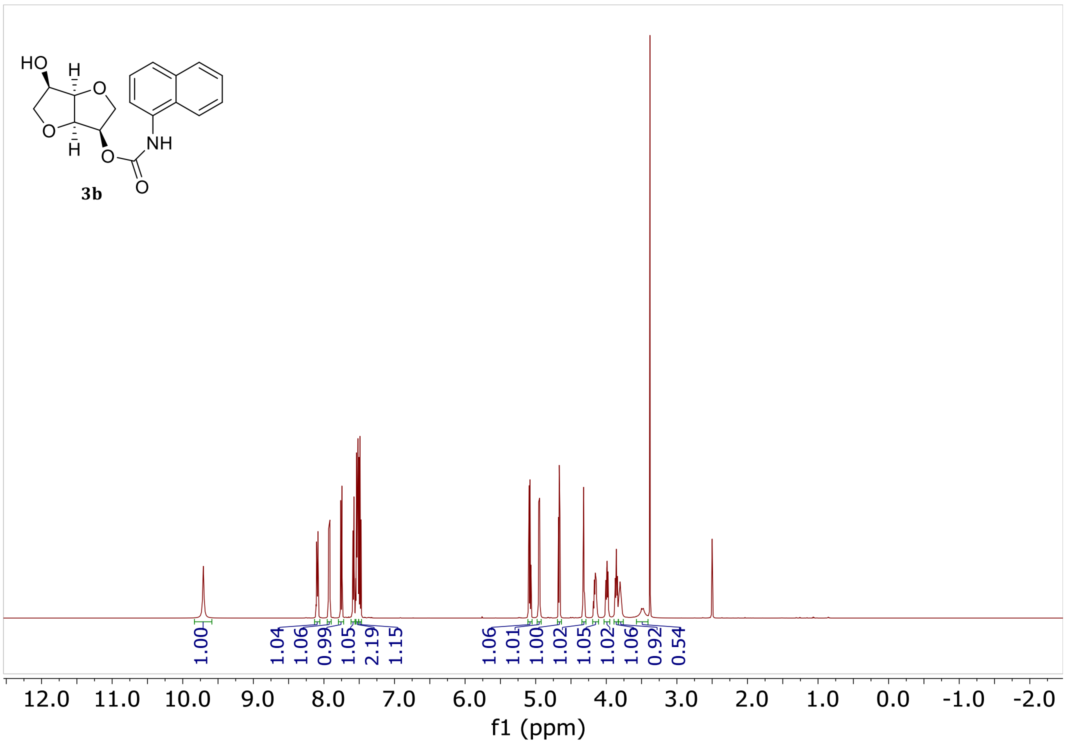

Figure S7. ^{1}H NMR (500 MHz, DMSO-d_6) spectrum of compound **3b**

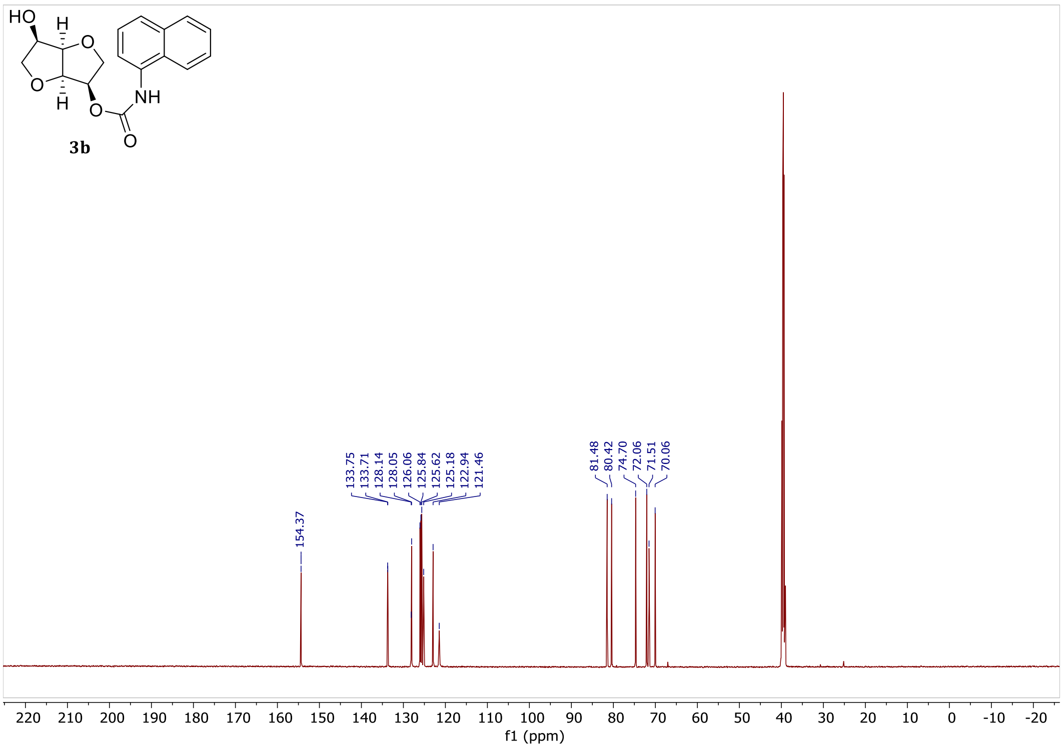

Figure S8. $^{13}C\{^{1}H\}$ NMR (126 MHz, DMSO-d_6) spectrum of compound **3b**

(3R,3aR,6S,6aR)-hexaydrofuro[3,2-b]furan-6-(1-naphthyl)carbamoyl-3-ol (5b)

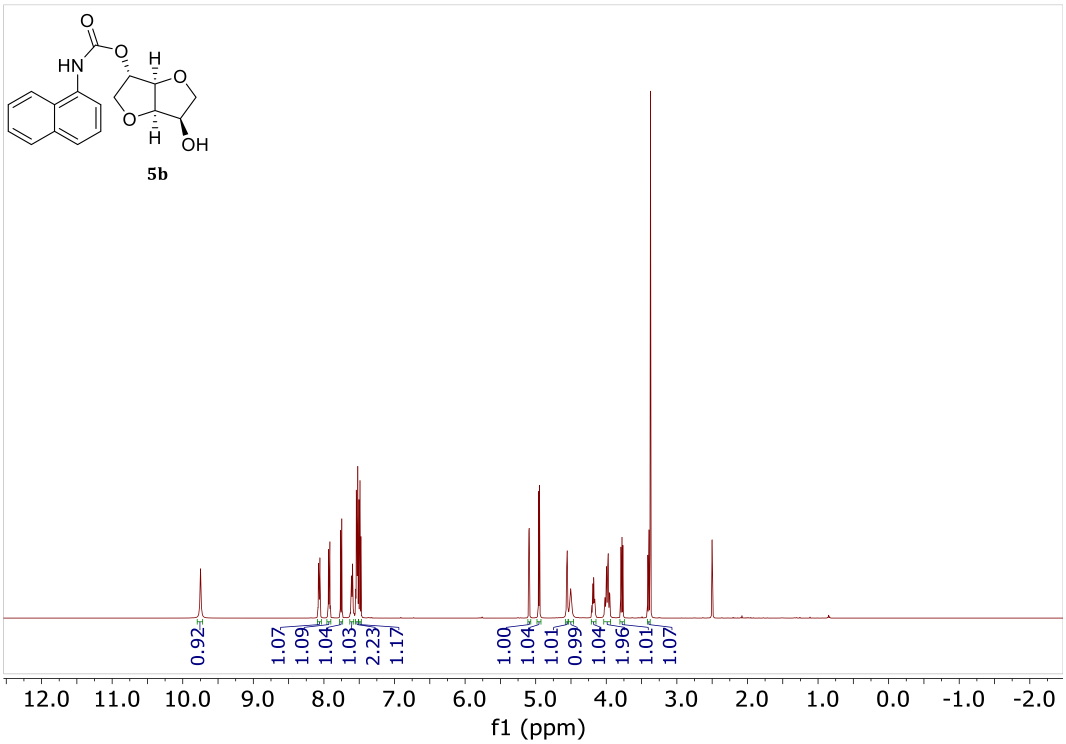

Figure S9. ^{1}H NMR (500 MHz, DMSO-d_6) spectrum of compound **5b**

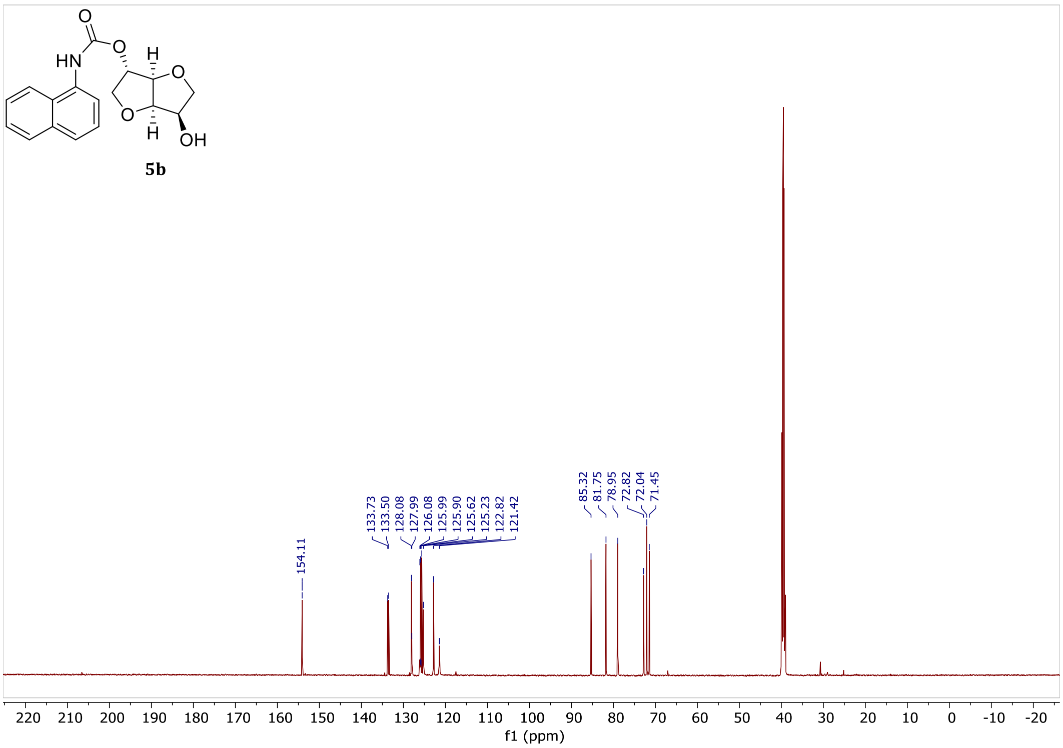

Figure S10. ^{13}C{^{1}H} NMR (126 MHz, DMSO-d_6) spectrum of compound **5b**

(3R,3aR,6S,6aR)-hexaydrofuro[3,2-b]furan-3-(1-naphthyl)carbamoyl-6-ol 6b

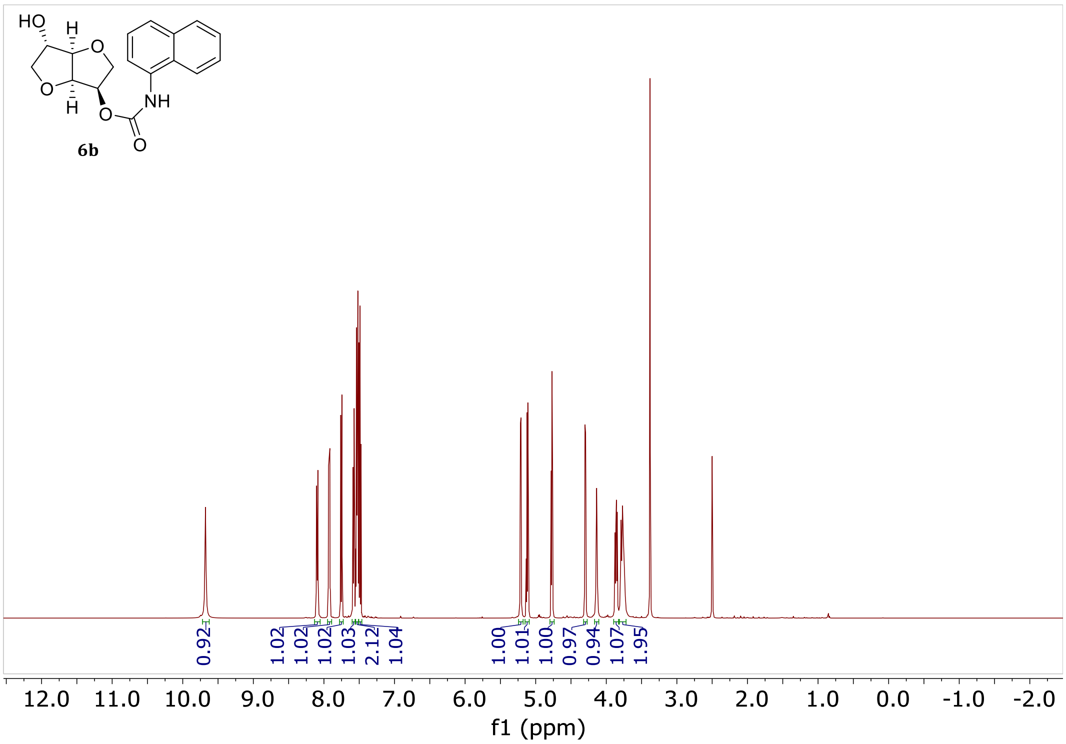

Figure S11. ^{1}H NMR (500 MHz, DMSO-d_6) spectrum of compound **6b**

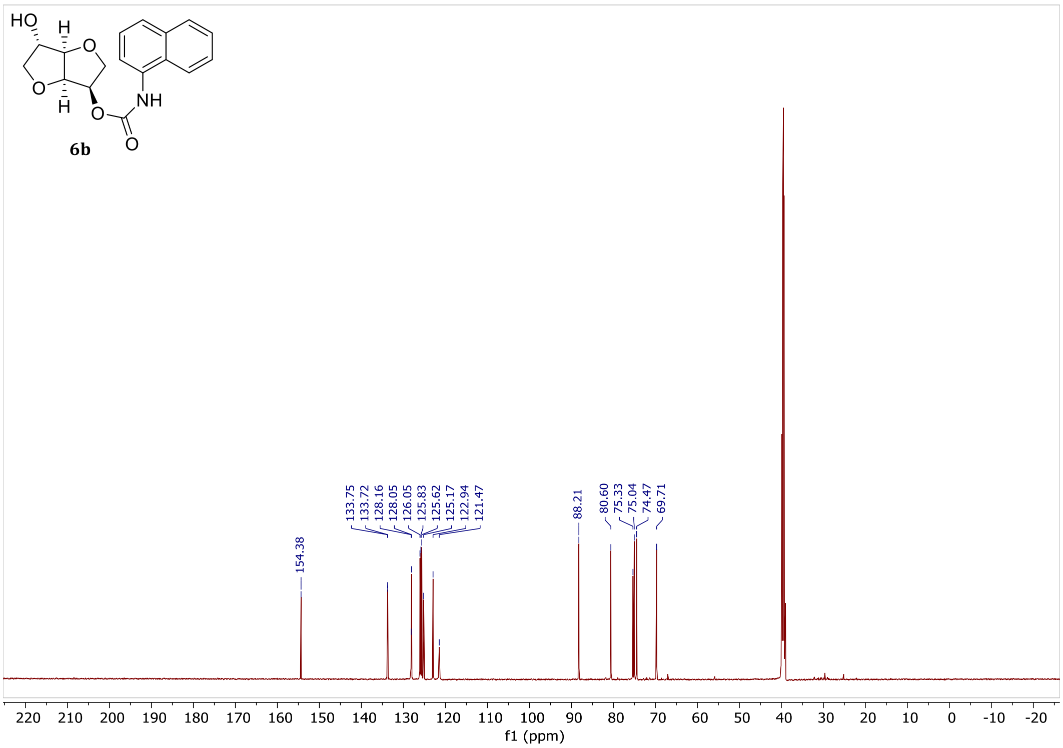

Figure S12. $^{13}C\{^{1}H\}$ NMR (126 MHz, DMSO-d_6) spectrum of compound **6b**

(3R,3aR,6R,6aR)-hexaydrofuro[3,2-b]furan-6-(3,5-bis(trifluoromethyl)phenyl) carbamoyl-3-ol (3c)

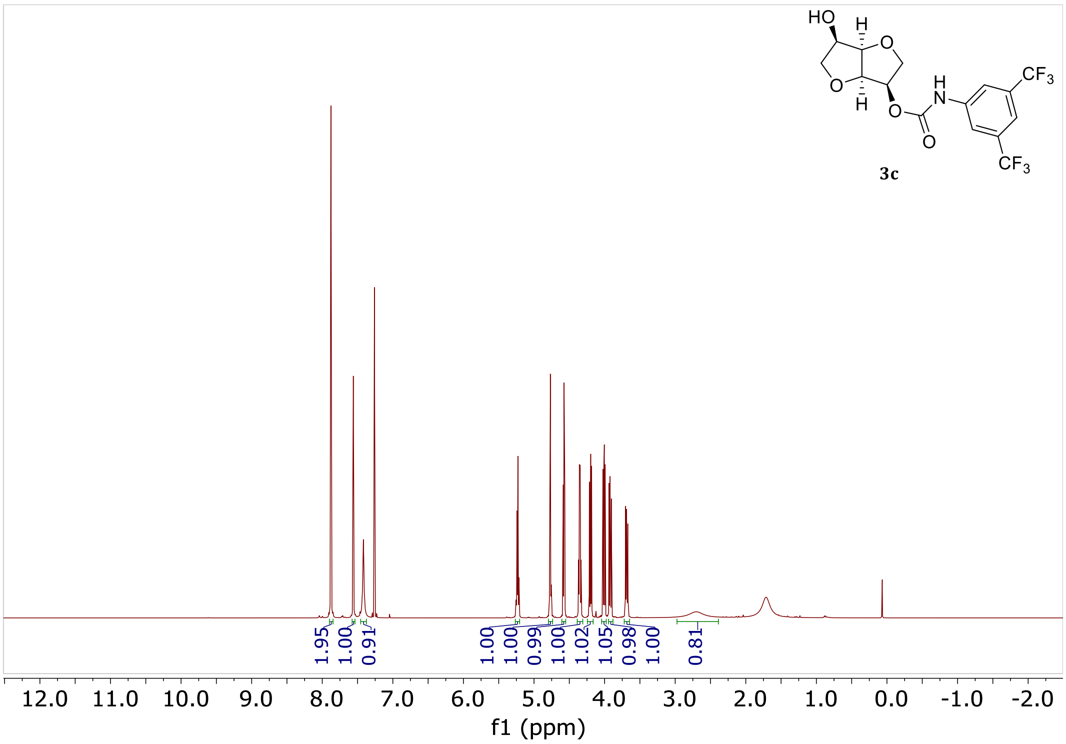

Figure S13. ^{1}H NMR (500 MHz, Chloroform-*d*) spectrum of compound **3c**

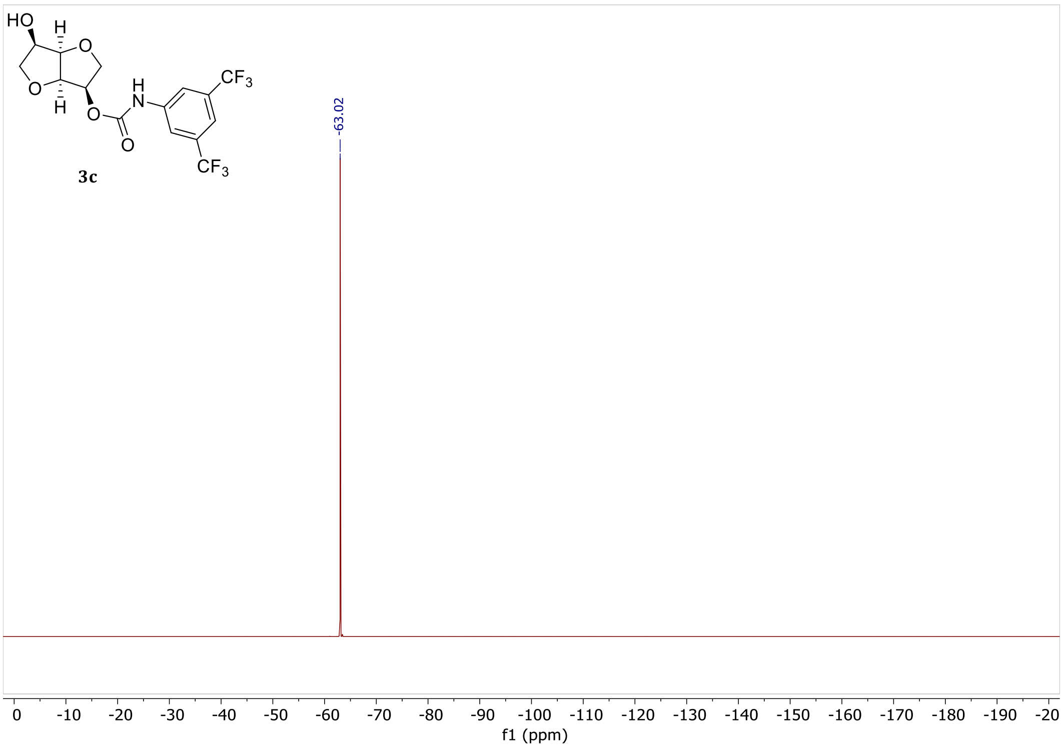

Figure S14. ^{19}F NMR (471 MHz, Chloroform-*d*) spectrum of compound **3c**

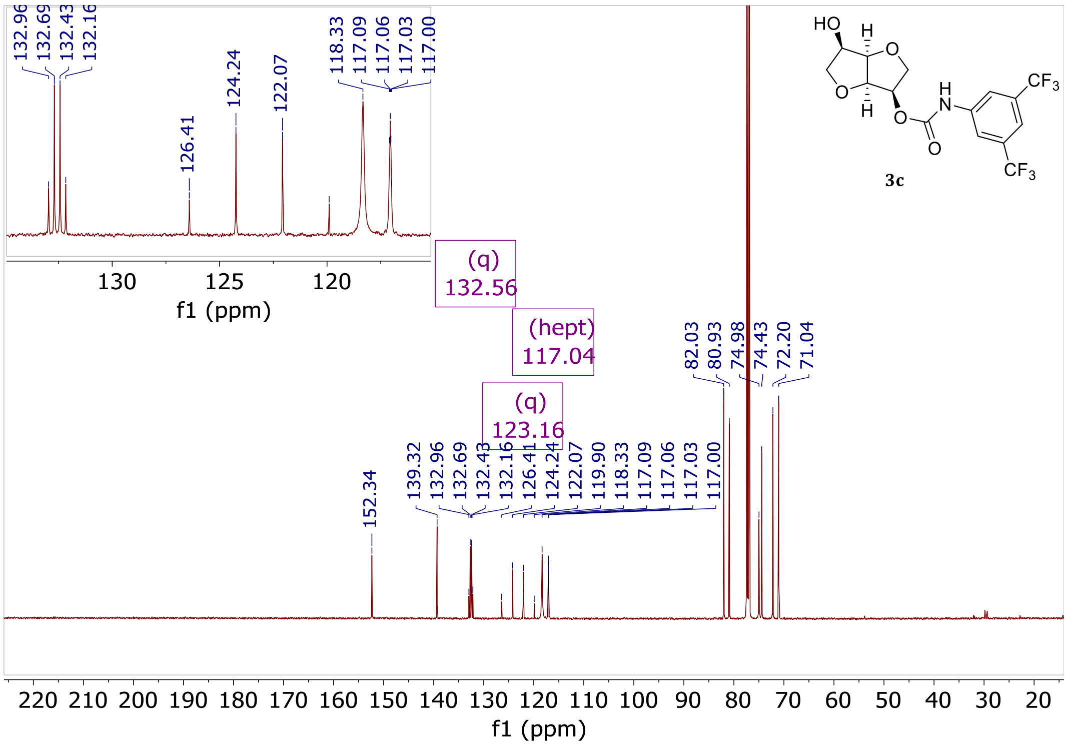

Figure S15. $^{13}C\{^{1}H\}$ NMR (126 MHz, Chloroform-*d*) spectrum of compound **3c**

(3R,3aR,6S,6aR)-hexaydrofuro[3,2-b]furan-6-(3,5-bis(trifluoromethyl)phenyl) carbamoyl-3-ol (5c)

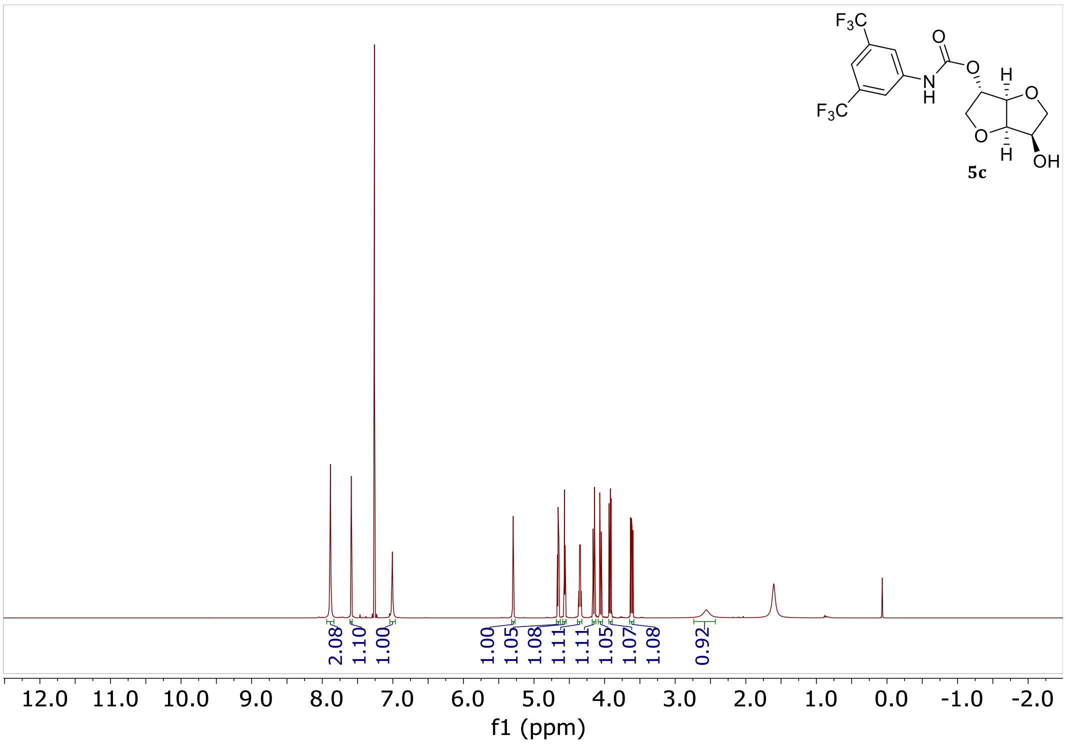

Figure S16. ^{1}H NMR (500 MHz, Chloroform-*d*) spectrum of compound **5c**

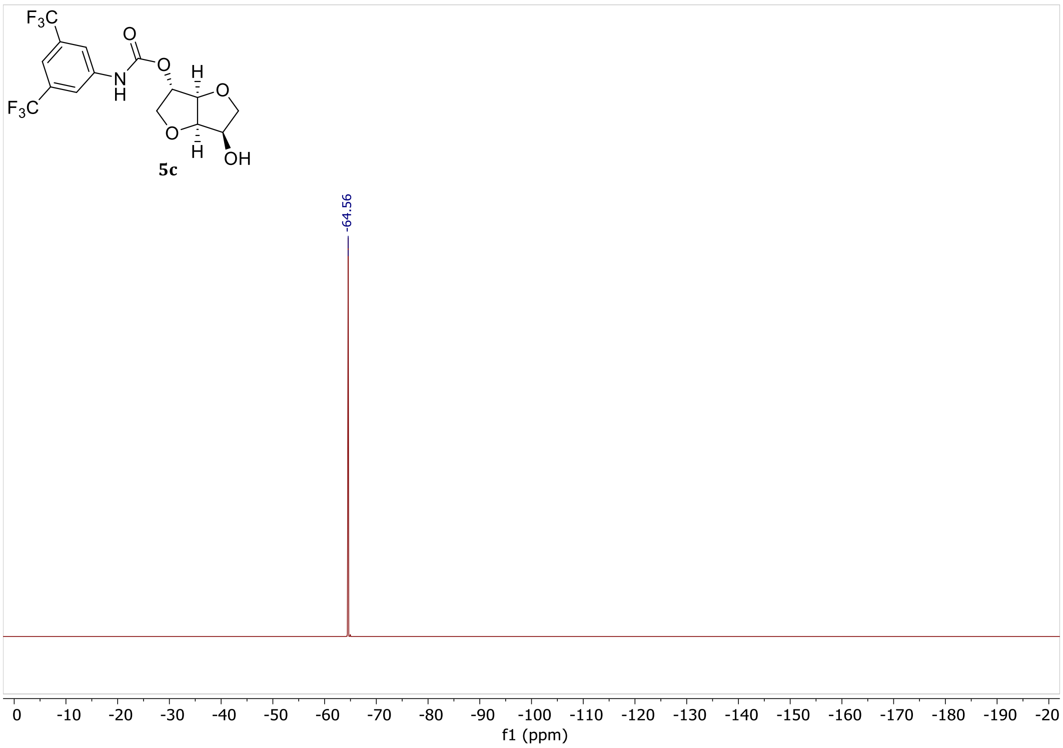

Figure S17. ^{19}F NMR (471 MHz, Methanol-*d*$_4$) spectrum of compound **5c**

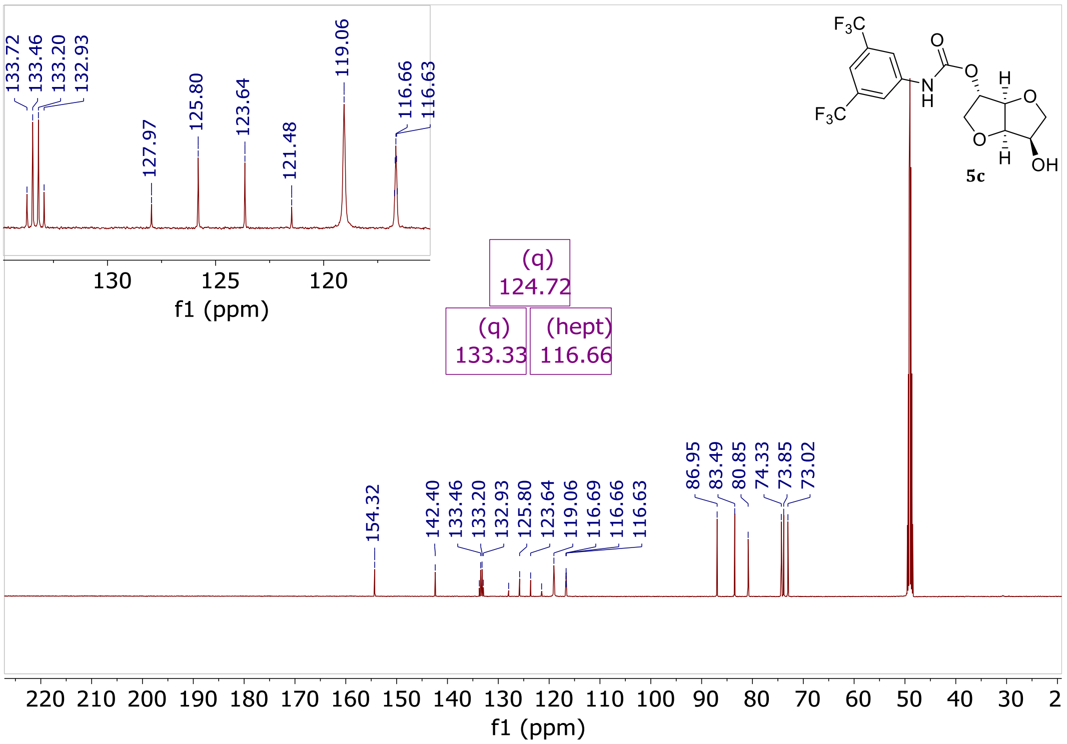

Figure S18. $^{13}C\{^1H\}$ NMR (126 MHz, Methanol-d_4) spectrum of compound **5c**

(3R,3aR,6S,6aR)-hexaydrofuro[3,2-b]furan-3-(3,5-bis(trifluoromethyl)phenyl) carbamoyl-6-ol (6c)

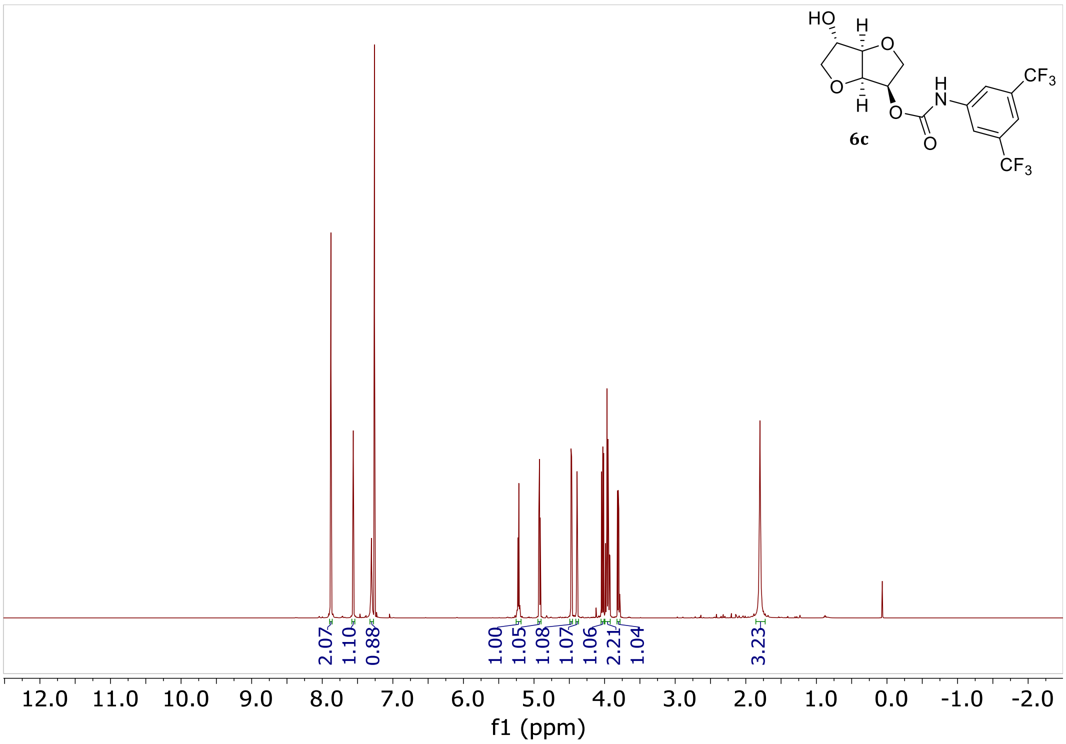

Figure S19. ^{1}H NMR (500 MHz, Chloroform-*d*) spectrum of compound **6c**

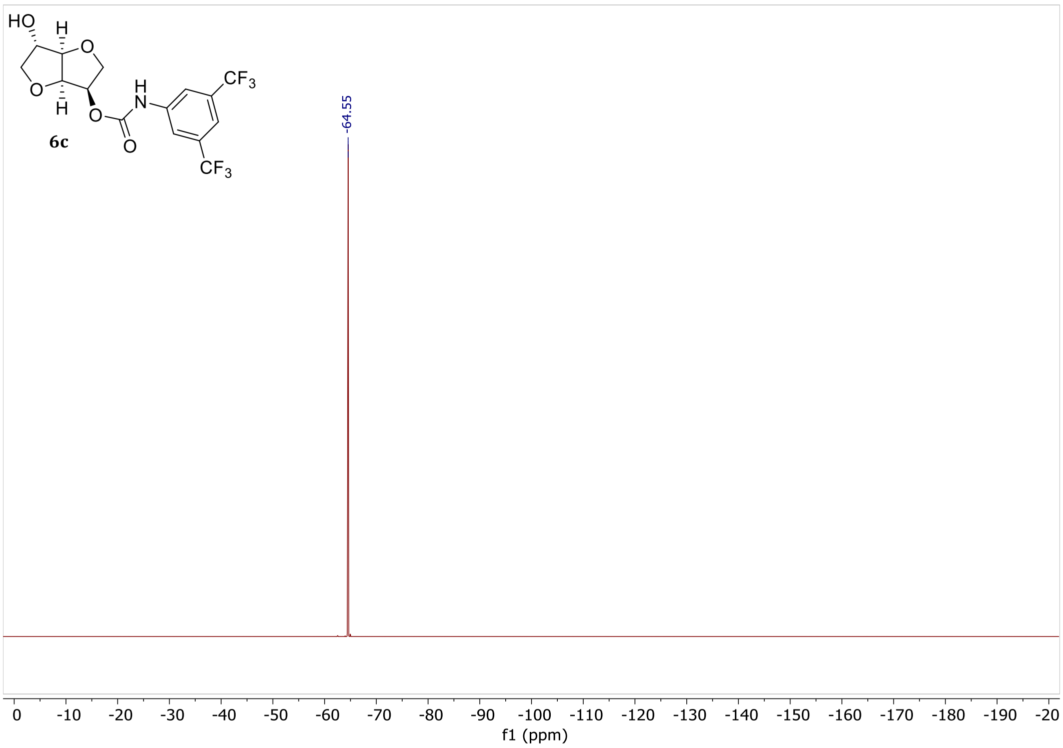

Figure S20. ^{19}F NMR (471 MHz, Methanol-d_4) spectrum of compound **6c**

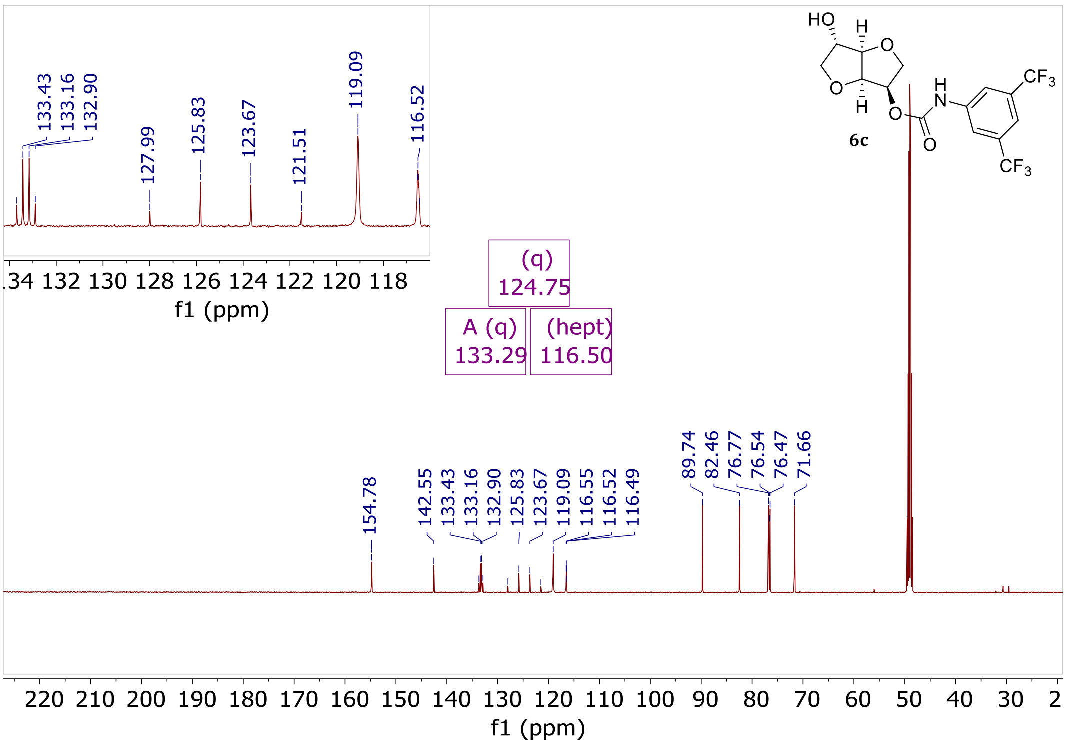

Figure S21. $^{13}C\{^{1}H\}$ NMR (126 MHz, Methanol-d_4) spectrum of compound **6c**

(3R,3aR,6R,6aR)-hexaydrofuro[3,2-b]furan-6-(3,5-dimethoxyphenyl)carbamoyl-3-ol (3d)

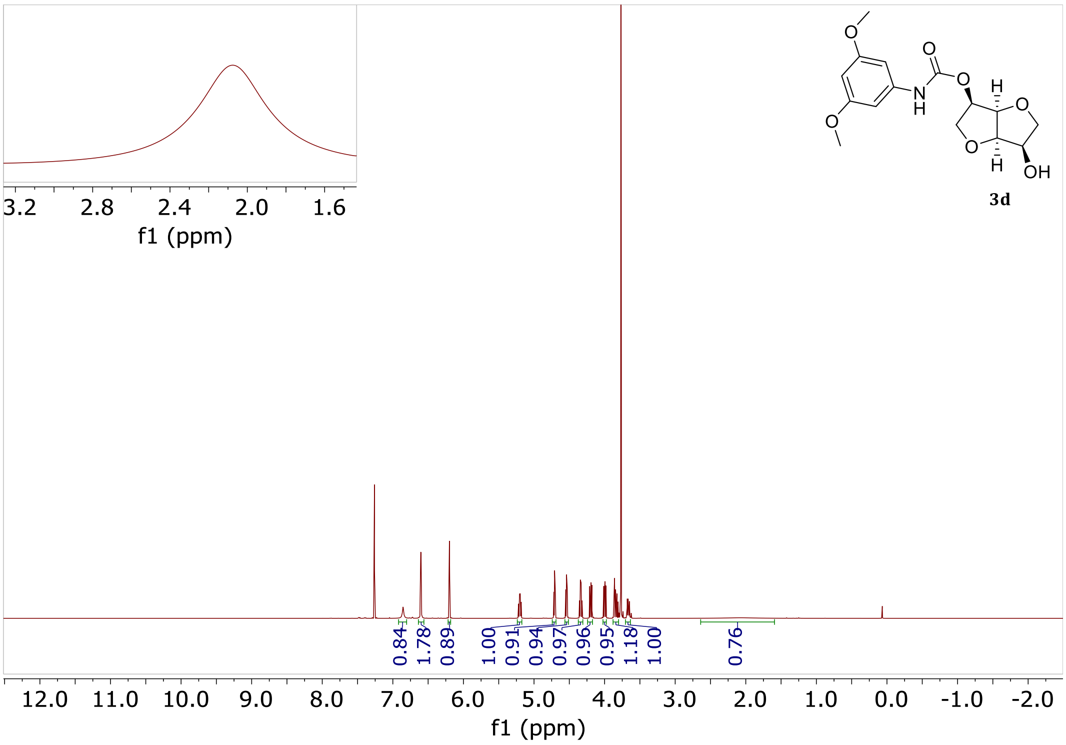

Figure S22. ^{1}H NMR (500 MHz, Chloroform-*d*) spectrum of compound **3d**

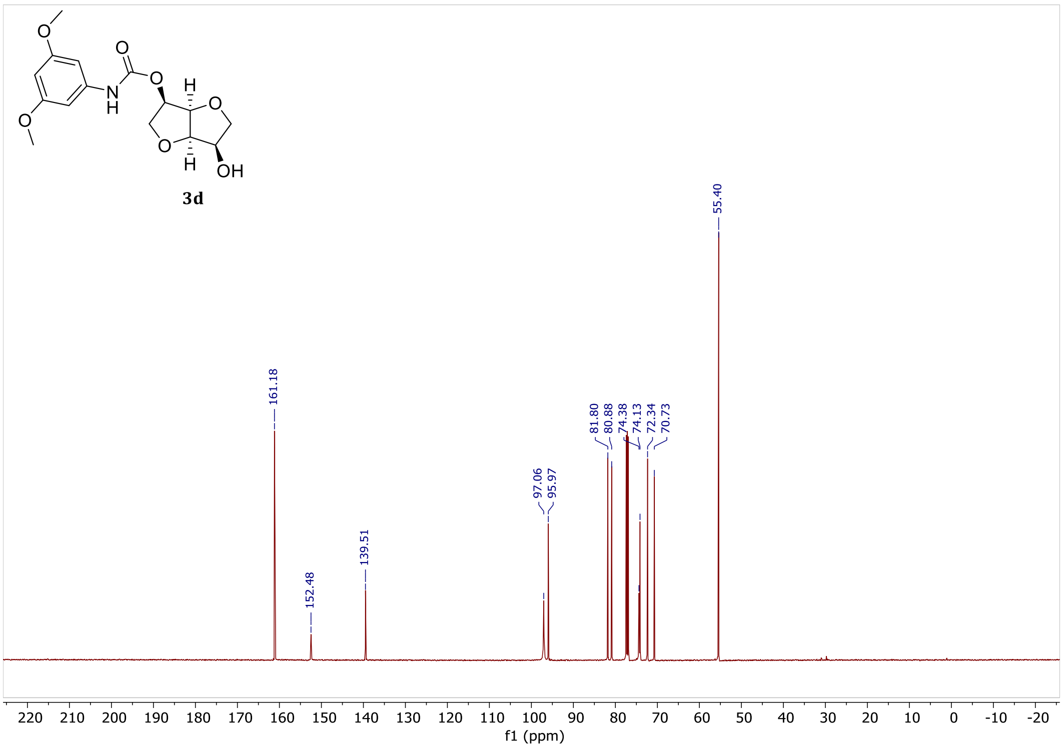

Figure S23. $^{13}C\{^{1}H\}$ NMR (126 MHz, Chloroform-*d*) spectrum of compound **3d**

(3R,3aR,6S,6aR)-hexaydrofuro[3,2-b]furan-6-(3,5-dimethoxyphenyl)carbamoyl-3-ol (5d)

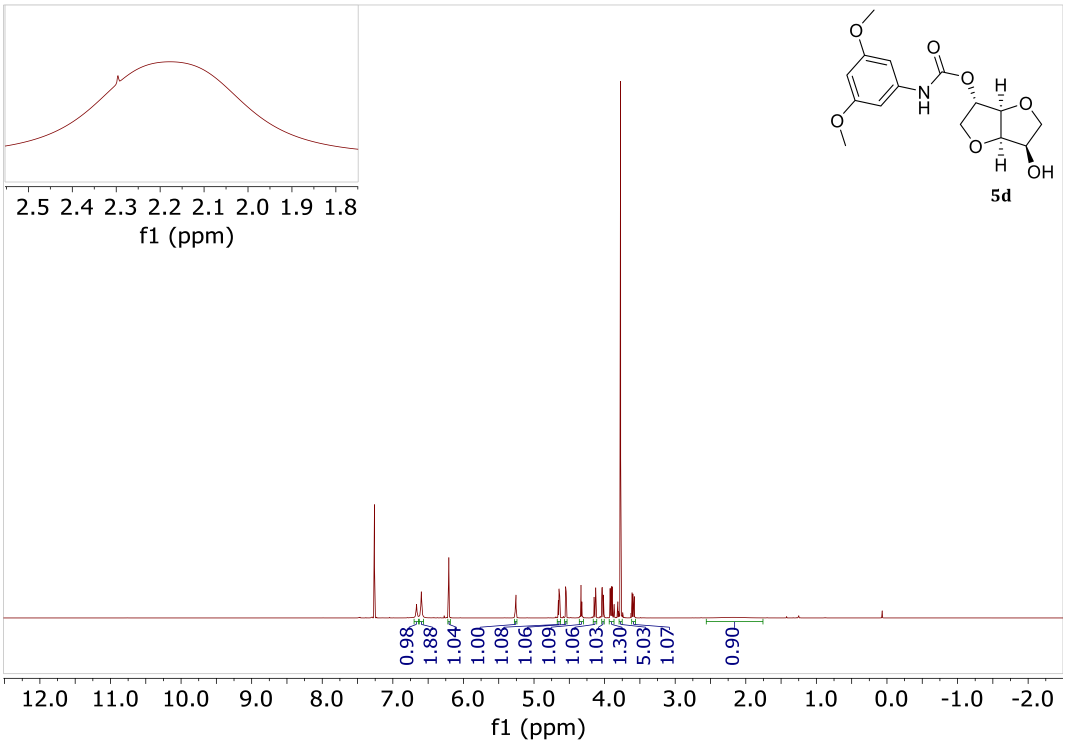

Figure S24. ^{1}H NMR (500 MHz, Chloroform-*d*) spectrum of compound **5d**

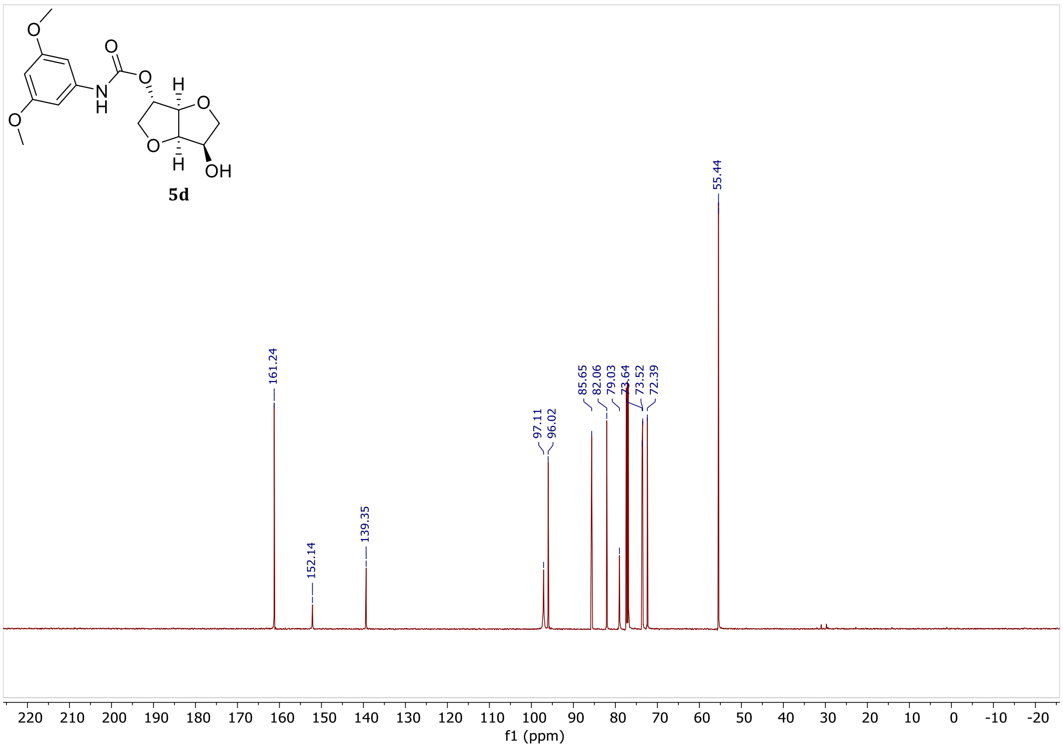

Figure S25. ^{13}C{^{1}H} NMR (126 MHz, Chloroform-*d*) spectrum of compound **5d**

(3R,3aR,6S,6aR)-hexaydrofuro[3,2-b]furan-3-(3,5-dimethoxyphenyl)carbamoyl-6-ol (6d)

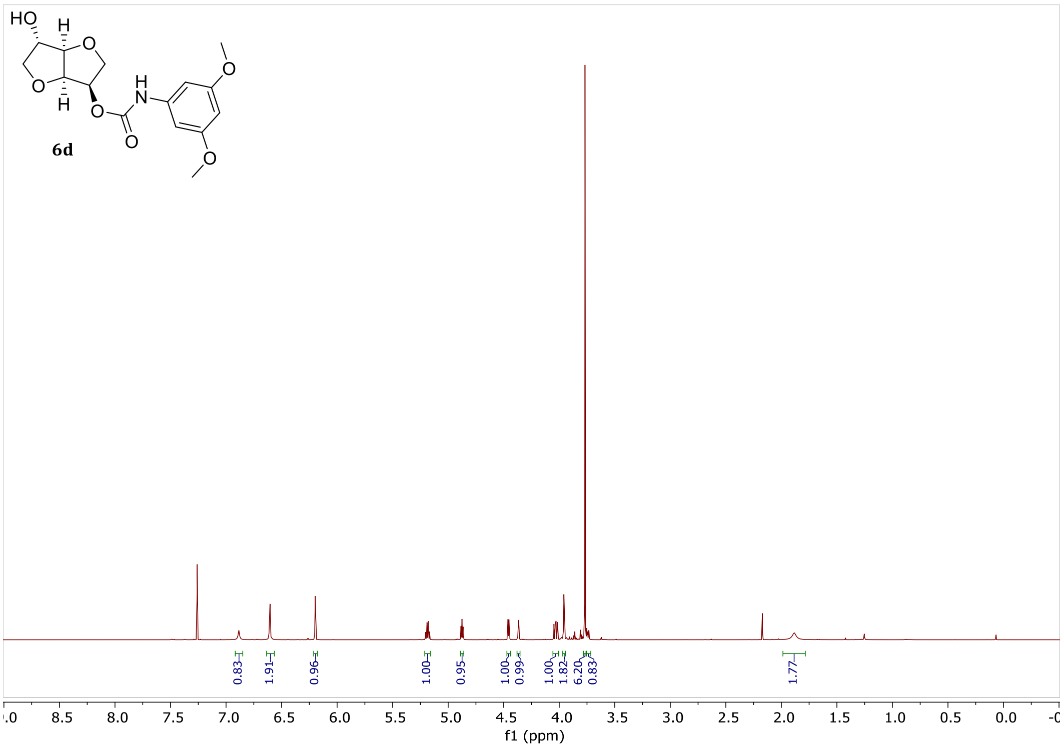

Figure S26. ^{1}H NMR (500 MHz, Chloroform-*d*) spectrum of compound **6d**

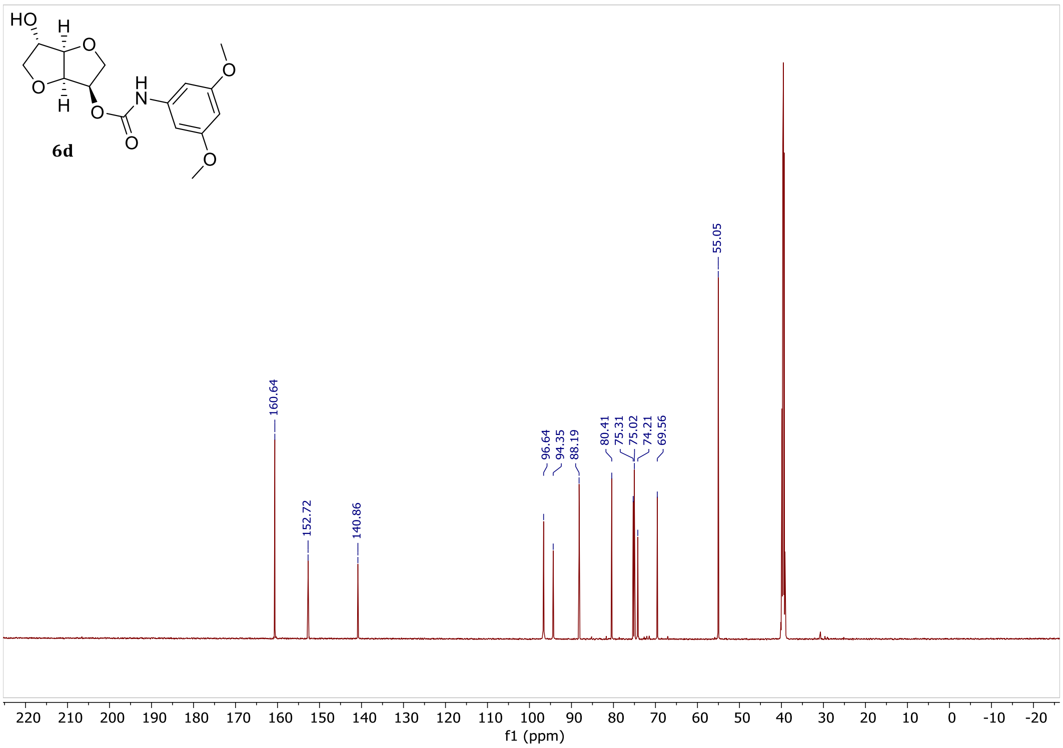

Figure S27. ^{13}C{^{1}H} NMR (126 MHz, Chloroform-*d*) spectrum of compound **6d**

(3R,3aR,6R,6aR)-hexaydrofuro[3,2-b]furan-6-(p-toluensulfonyl)carbamoyl-3-ol (3e)

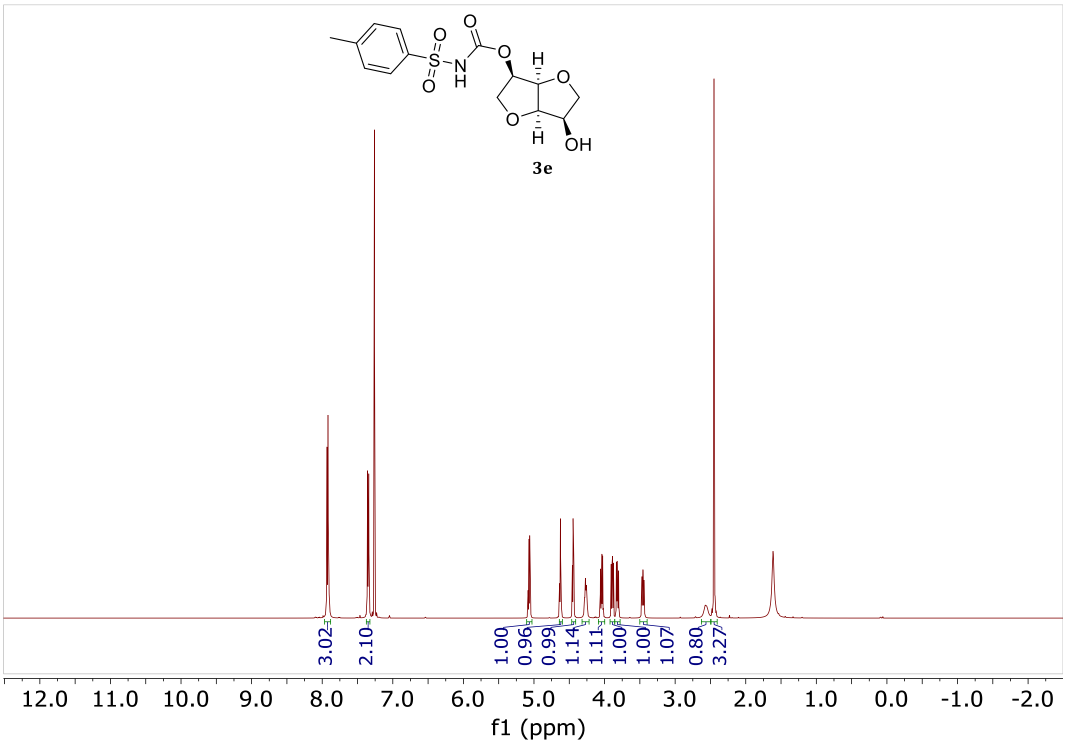

Figure S28. ^{1}H NMR (500 MHz, Chloroform-*d*) spectrum of compound **3e**

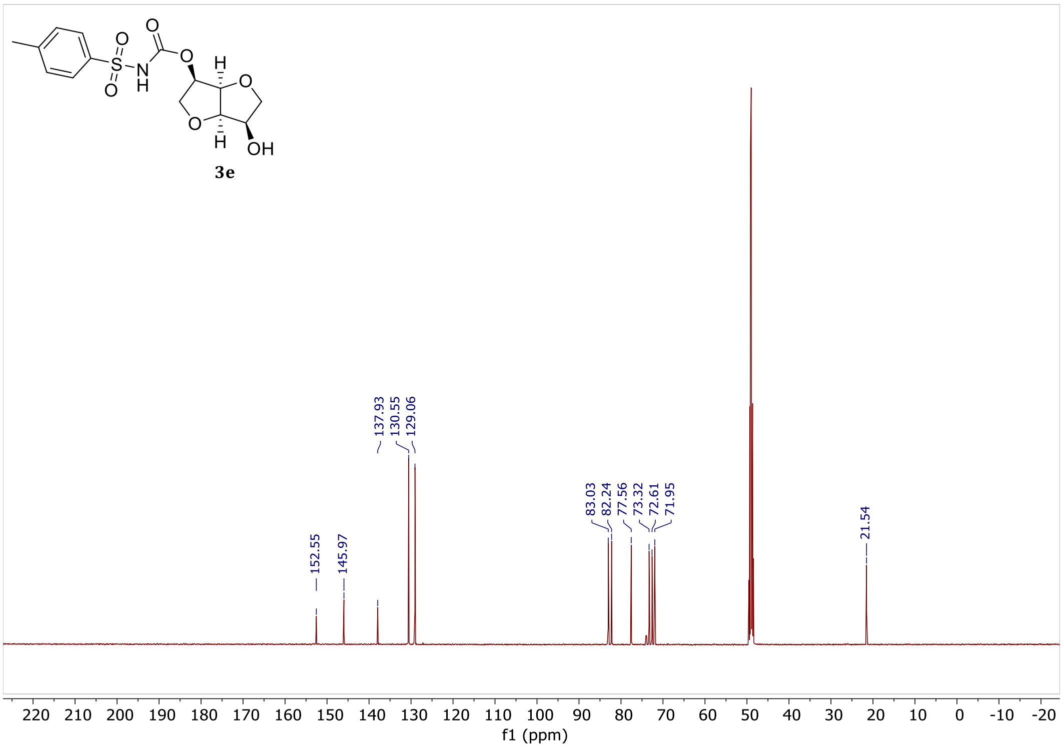

Figure S29. $^{13}C\{^{1}H\}$ NMR (126MHz, Methanol-d_4) spectrum of compound **3e**

(3R,3aR,6S,6aR)-hexaydrofuro[3,2-b]furan-6-(p-toluensulfonyl)carbamoyl-3-ol (5e)

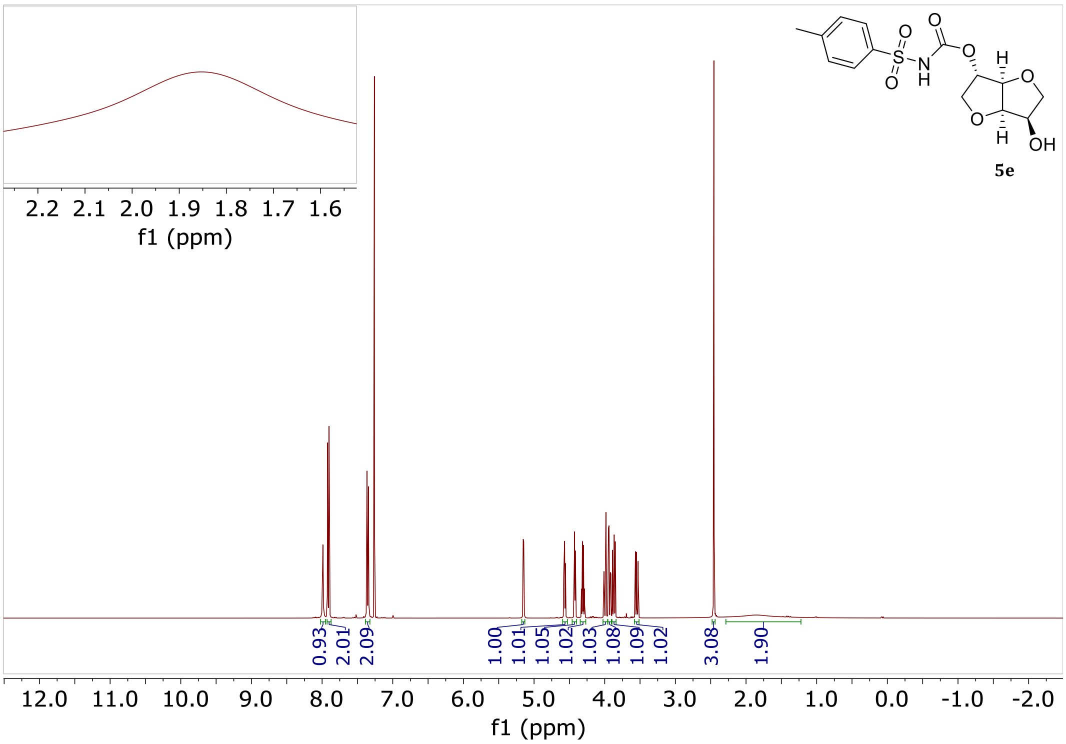

Figure S30. ^{1}H NMR (500 MHz, Chloroform-*d*) spectrum of compound **5e**

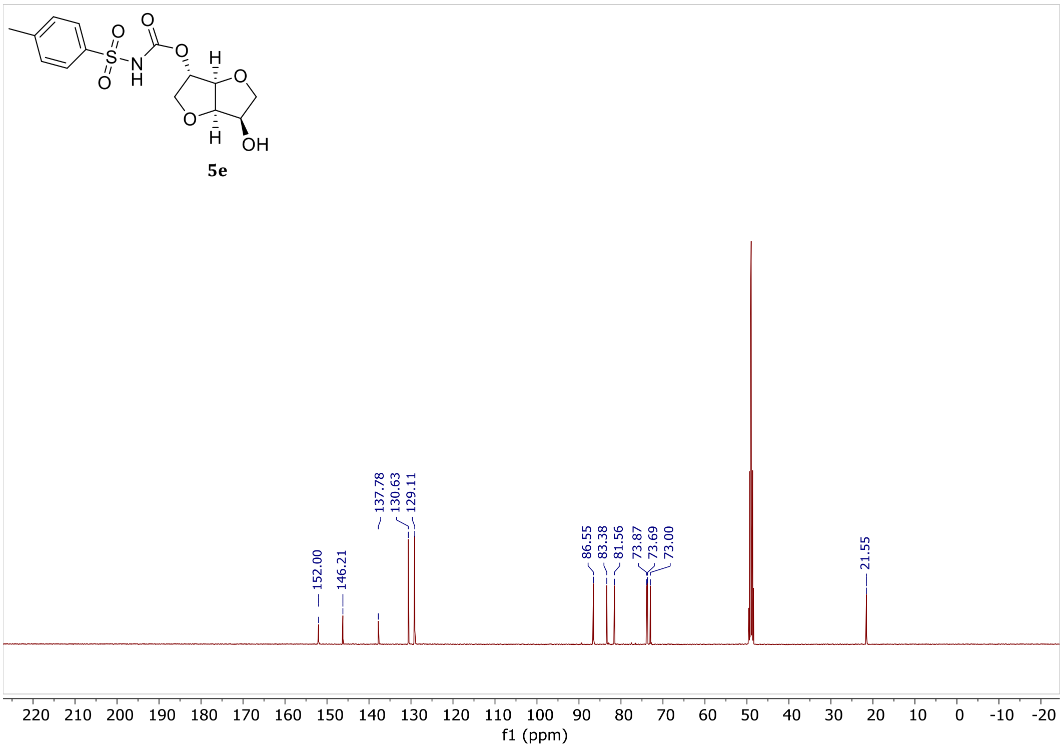

Figure S31. $^{13}C\{^{1}H\}$ NMR (126 MHz, Methanol-d_4) spectrum of compound **5e**

(3R,3aR,6S,6aR)-hexaydrofuro[3,2-b]furan-3-(p-toluensulfonyl)carbamoyl-6-ol (6e)

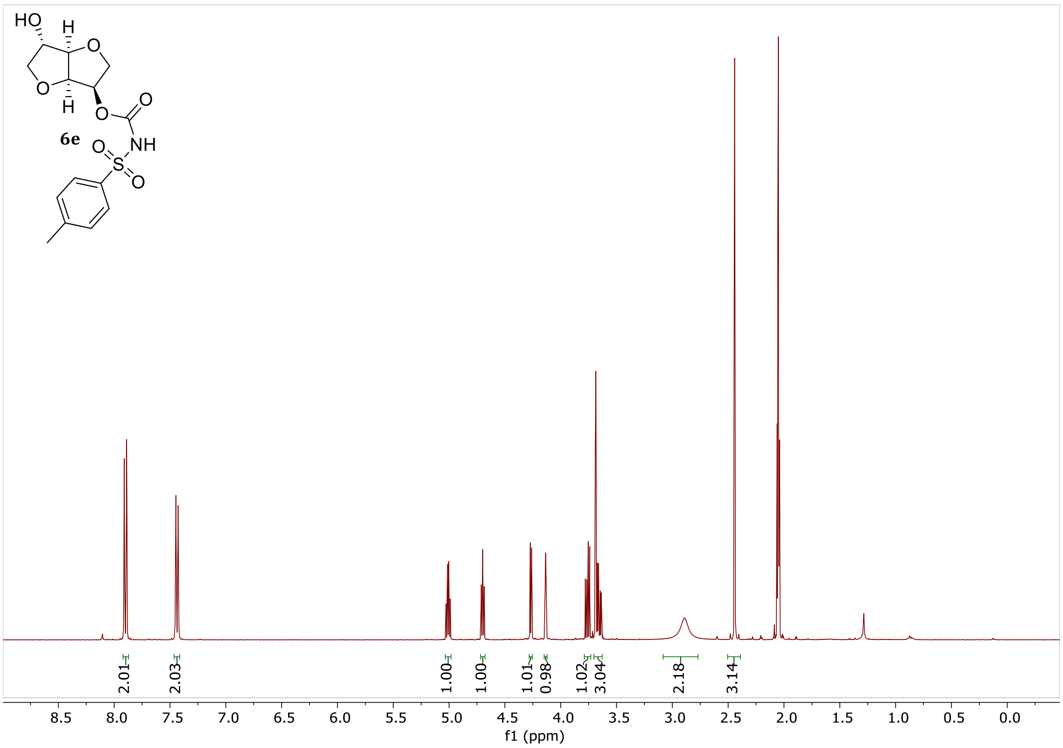

Figure S32. ^{1}H NMR (500 MHz, Acetone-d_6) spectrum of compound **6e**

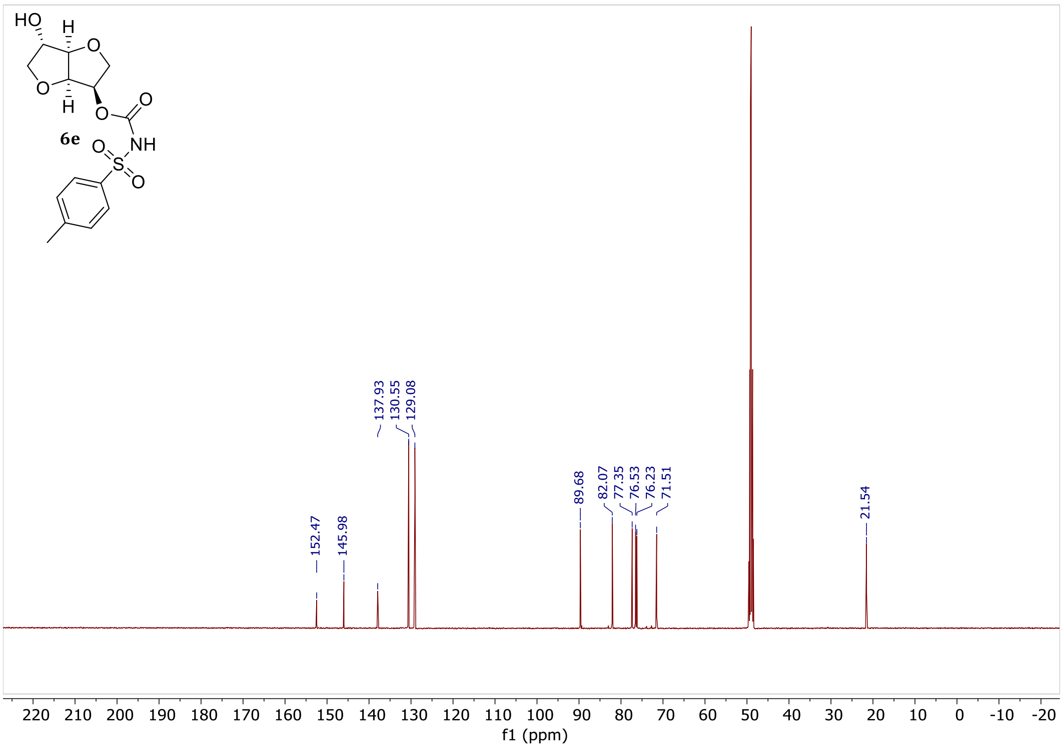

Figure S33. ^{13}C{^{1}H} NMR (126 MHz, Methanol-d_4) spectrum of compound **6e**

(3R,3aR,6R,6aR)-hexaydrofuro[3,2-b]furan-3,6-di-(3,5-dimethylphenyl)carbamate (4a)

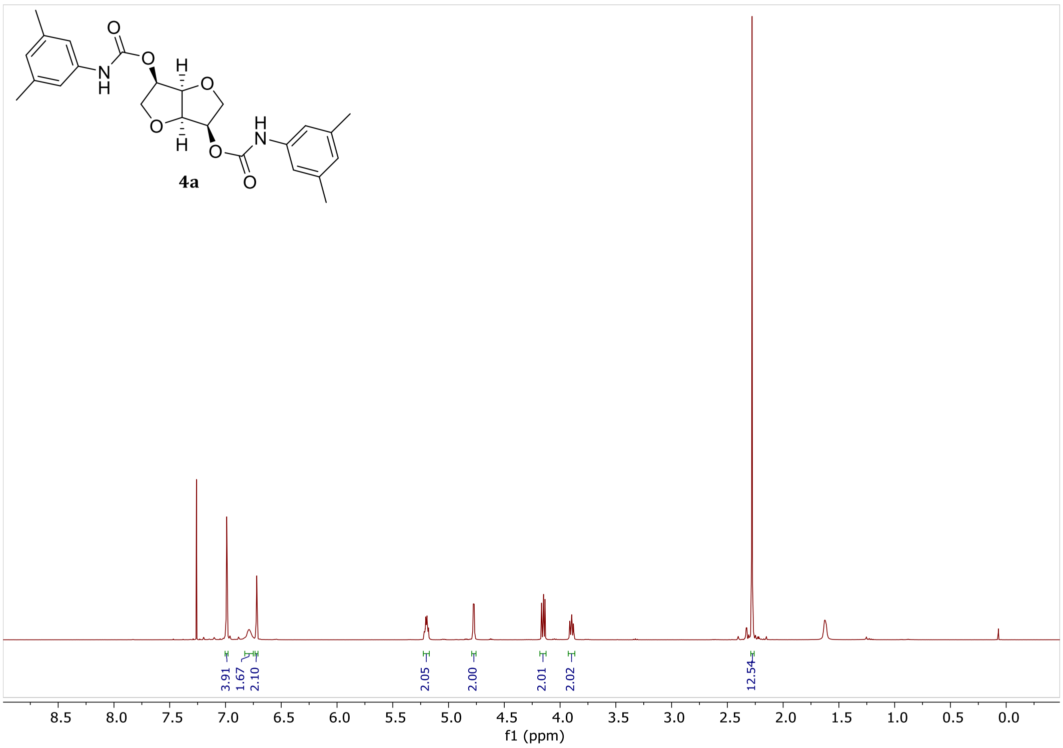

Figure S34. ^{1}H NMR (500 MHz, Chloroform-*d*) spectrum of compound **4a**

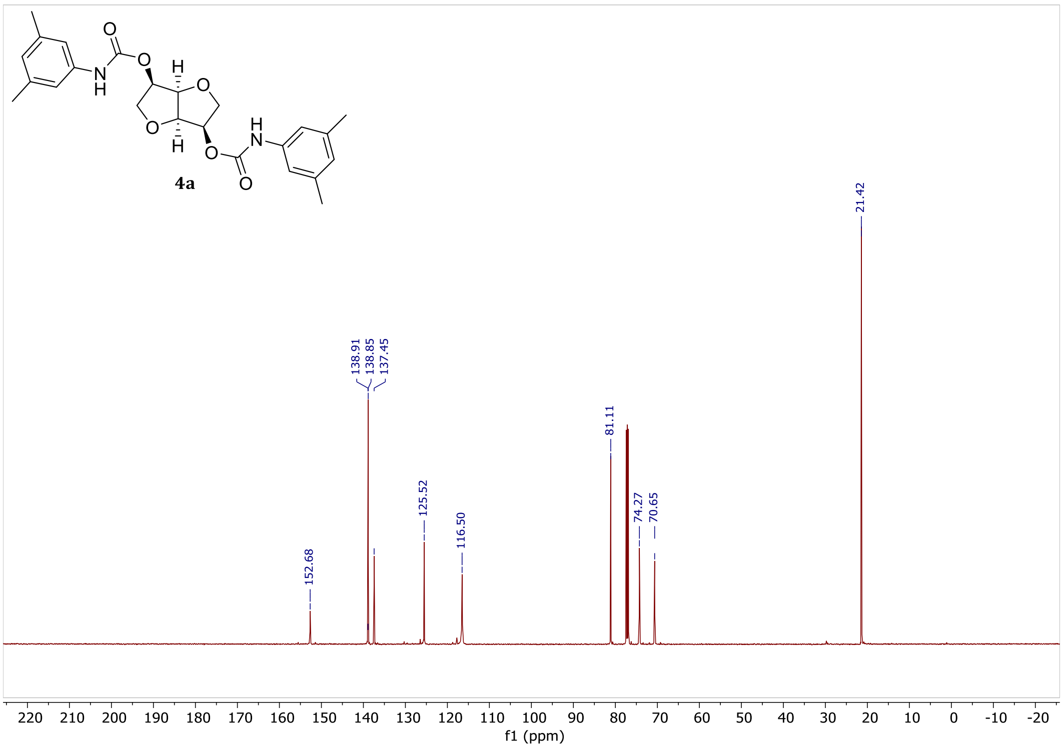

Figure S35. $^{13}C\{^{1}H\}$ NMR (126 MHz, Chloroform-*d*) spectrum of compound **4a**

(3R,3aR,6S,6aR)-hexaydrofuro[3,2-b]furan-3,6-di-(3,5-dimethylphenyl)carbamate (7a)

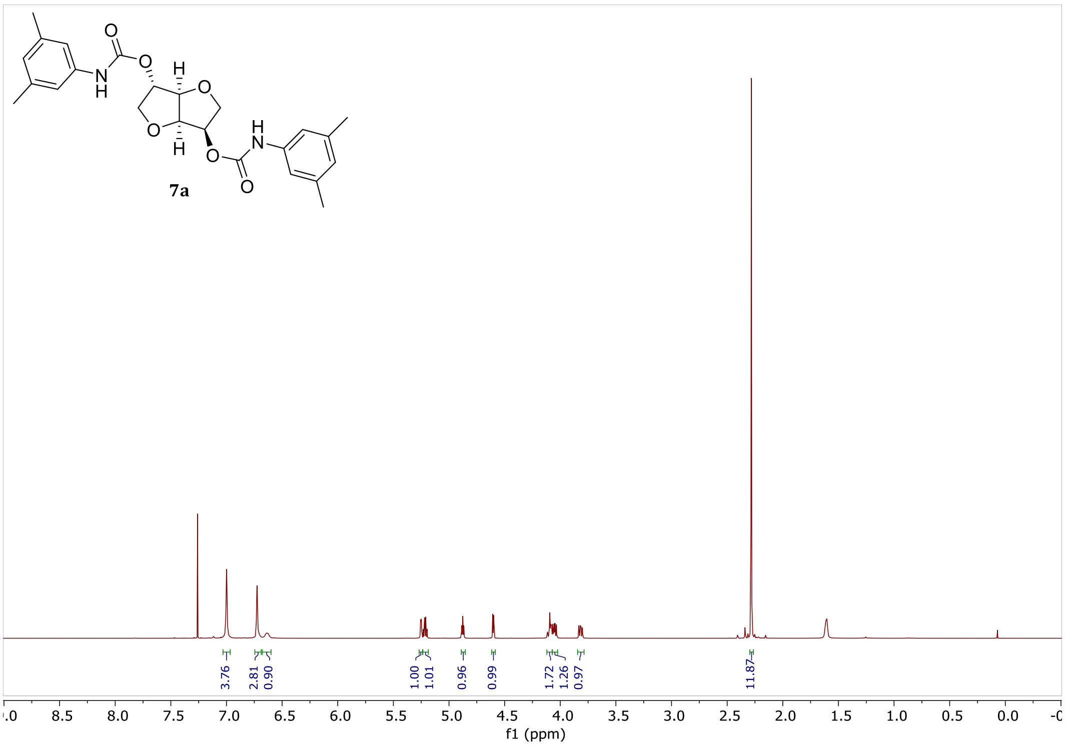

Figure S36. ^{1}H NMR (500 MHz, Chloroform-*d*) spectrum of compound **7a**

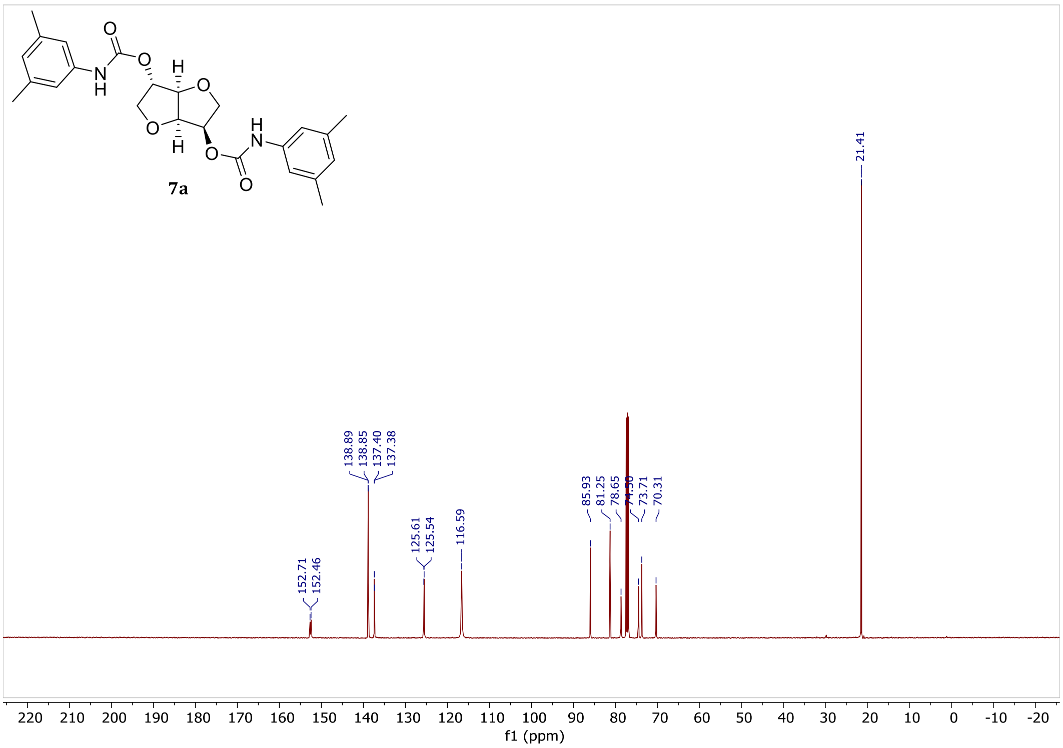

Figure S37. ^{13}C{^{1}H} NMR (126 MHz, Chloroform-*d*) spectrum of compound **7a**

(3R,3aR,6R,6aR)-hexaydrofuro[3,2-b]furan-3,6-di-(1-naphthyl)carbamate (4b)

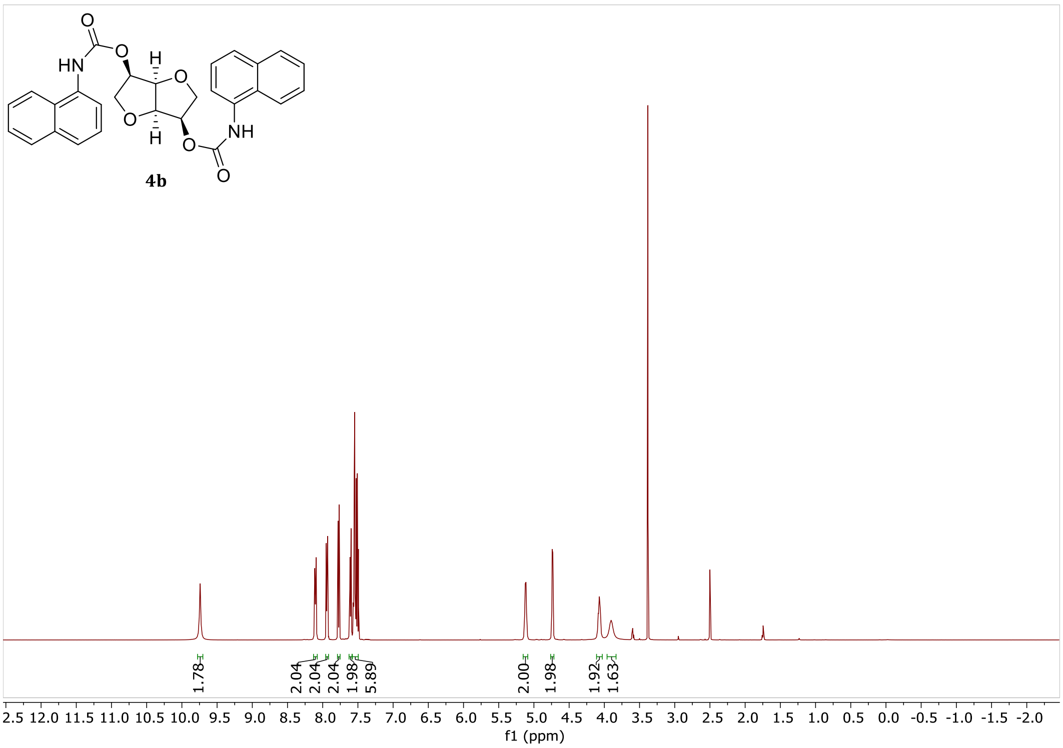

Figure S38. ^{1}H NMR (500 MHz, DMSO-d_6) spectrum of compound **4b**

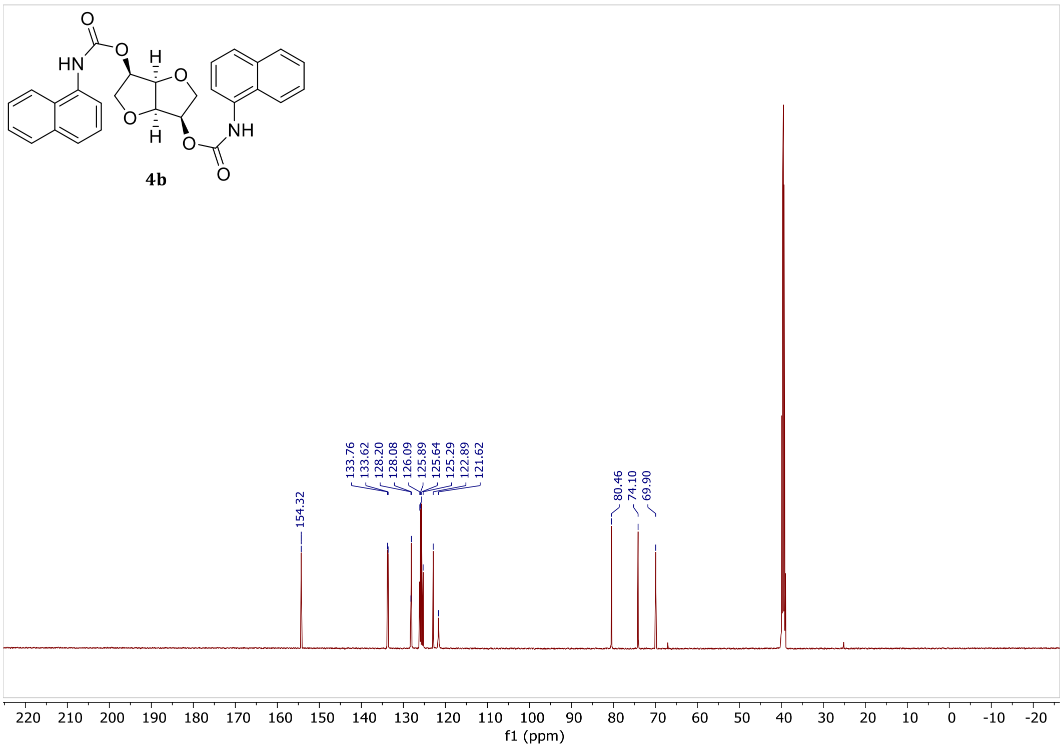

Figure S39. $^{13}C\{^{1}H\}$ NMR (126 MHz, DMSO-d_6) spectrum of compound **4b**

(3R,3aR,6S,6aR)-hexaydrofuro[3,2-b]furan-3,6-di-(1-naphthyl)carbamate (7b)

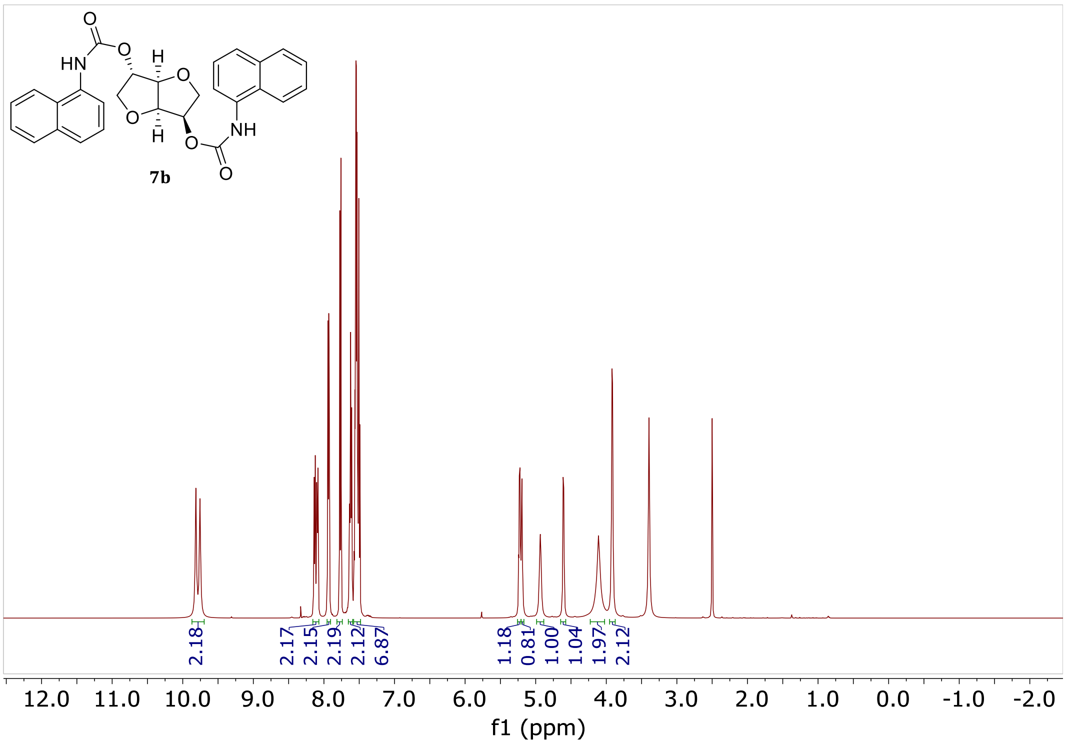

Figure S40. ^{1}H NMR (500 MHz, DMSO-d_6) spectrum of compound **7b**

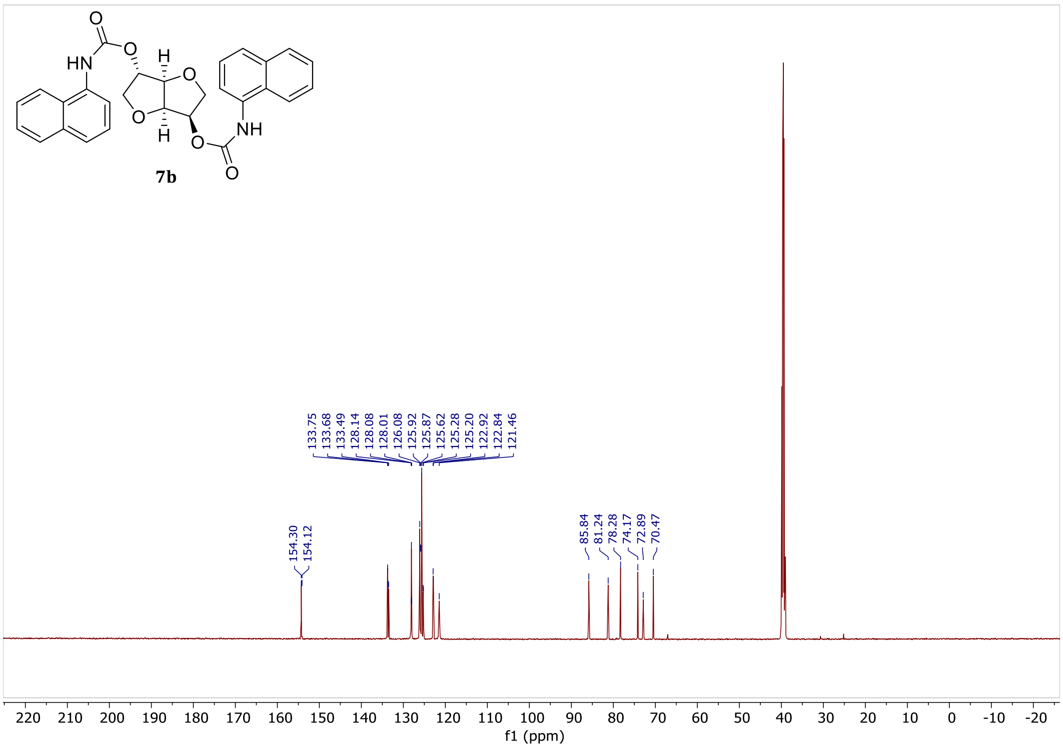

Figure S41. $^{13}C\{^{1}H\}$ NMR (126 MHz, DMSO-d_6) spectrum of compound **7b**

(3R,3aR,6R,6aR)-hexaydrofuro[3,2-b]furan-3,6-di-(3,5-bis(trifluoromethyl)phenyl) carbamate (4c)

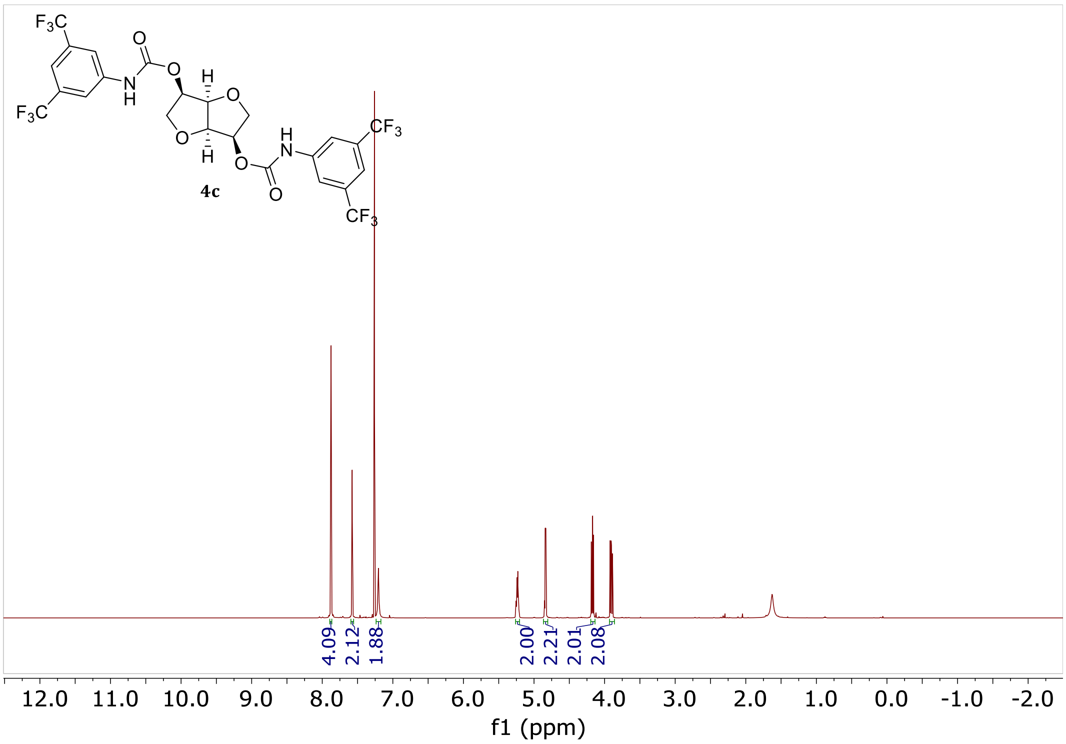

Figure S42. ^{1}H NMR (500 MHz, Chloroform-*d*) spectrum of compound **4c**

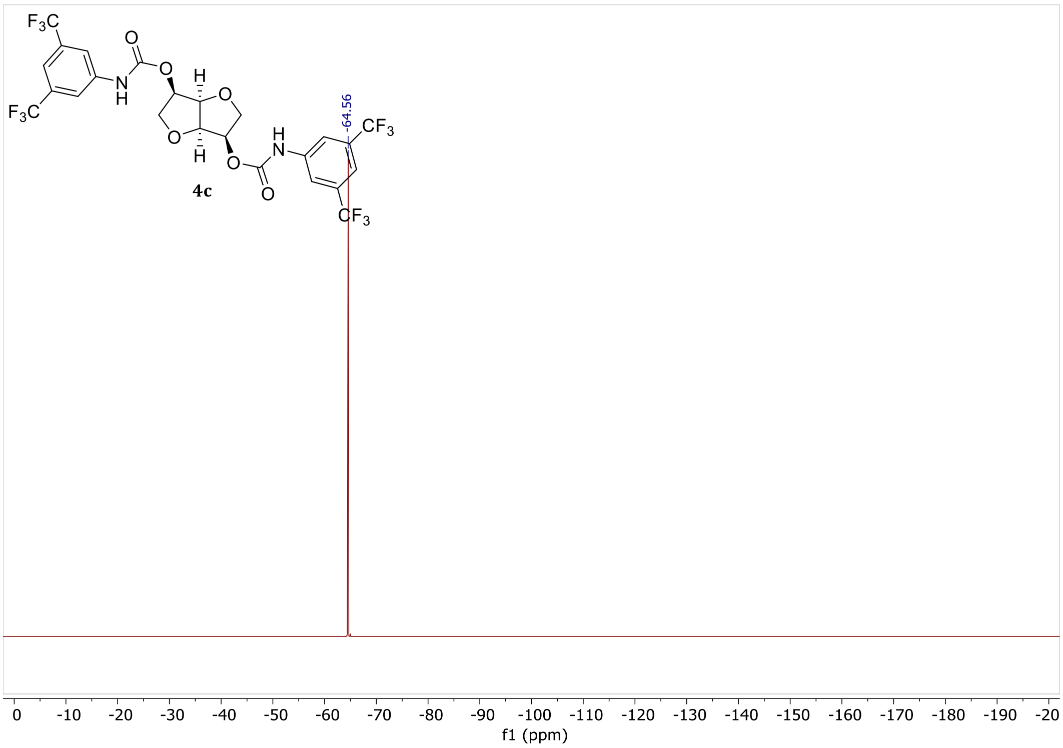

Figure S43. ^{19}F NMR (471 MHz, Methanol-*d*$_4$) spectrum of compound **4c**

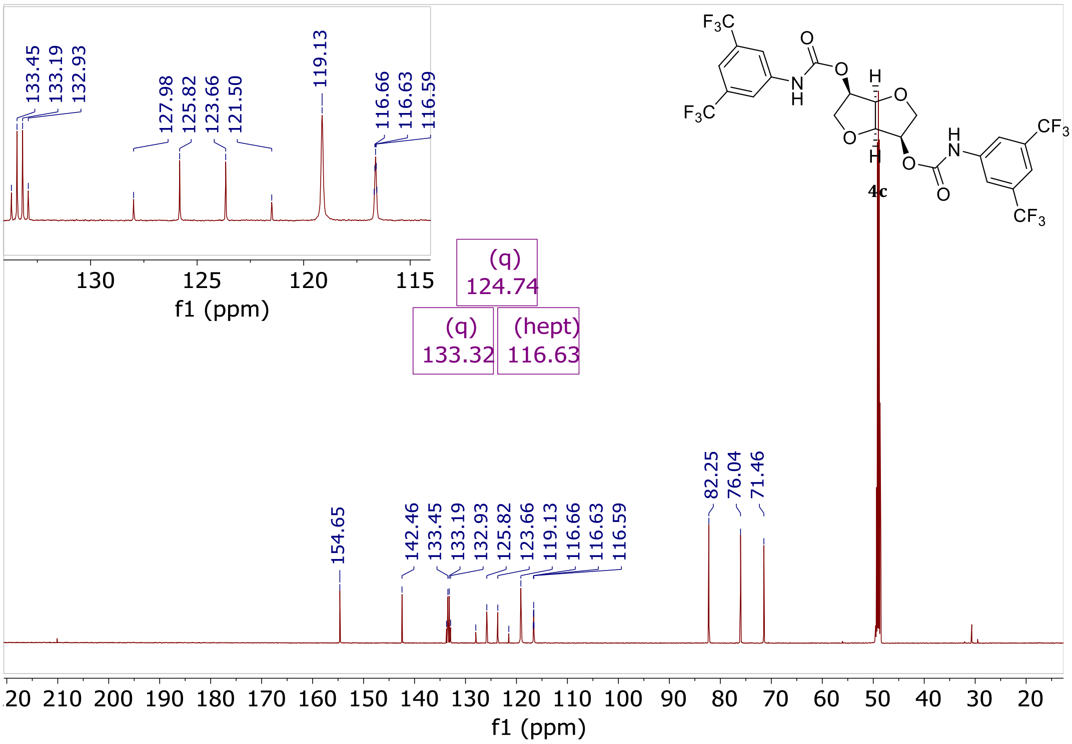

Figure S44. ${}^{13}C\{{}^{1}H\}$ NMR (126 MHz, Methanol-d_4) spectrum of compound **4c**.

(3R,3aR,6S,6aR)-hexaydrofuro[3,2-b]furan-3,6-di-(3,5-bis(trifluoromethyl)phenyl) carbamate (7c)

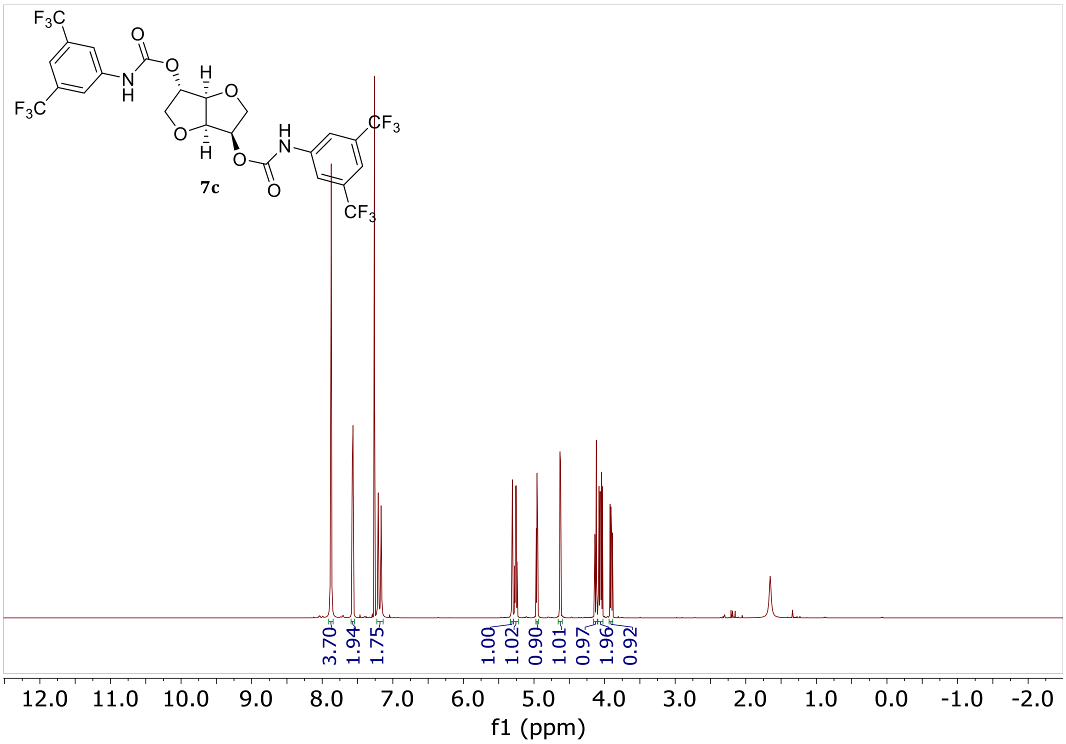

Figure S45. ^{1}H NMR (500 MHz, Chloroform-*d*) spectrum of compound **7c**

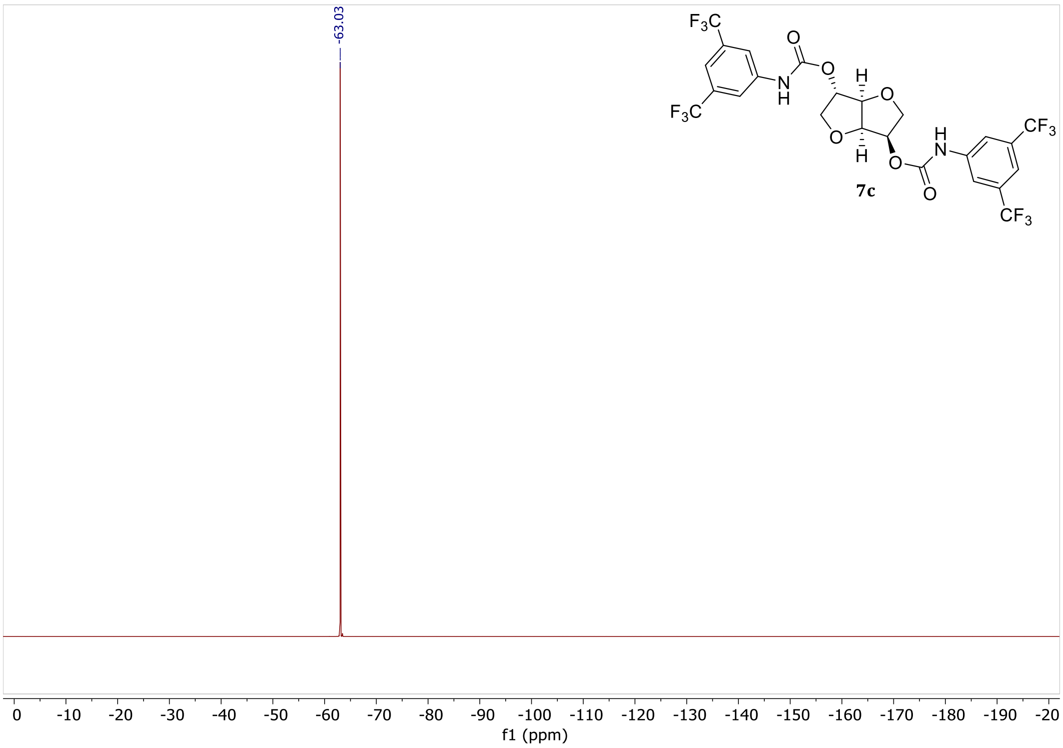

Figure S46. ^{19}F NMR (471 MHz, Chloroform-*d*) spectrum of compound **7c**

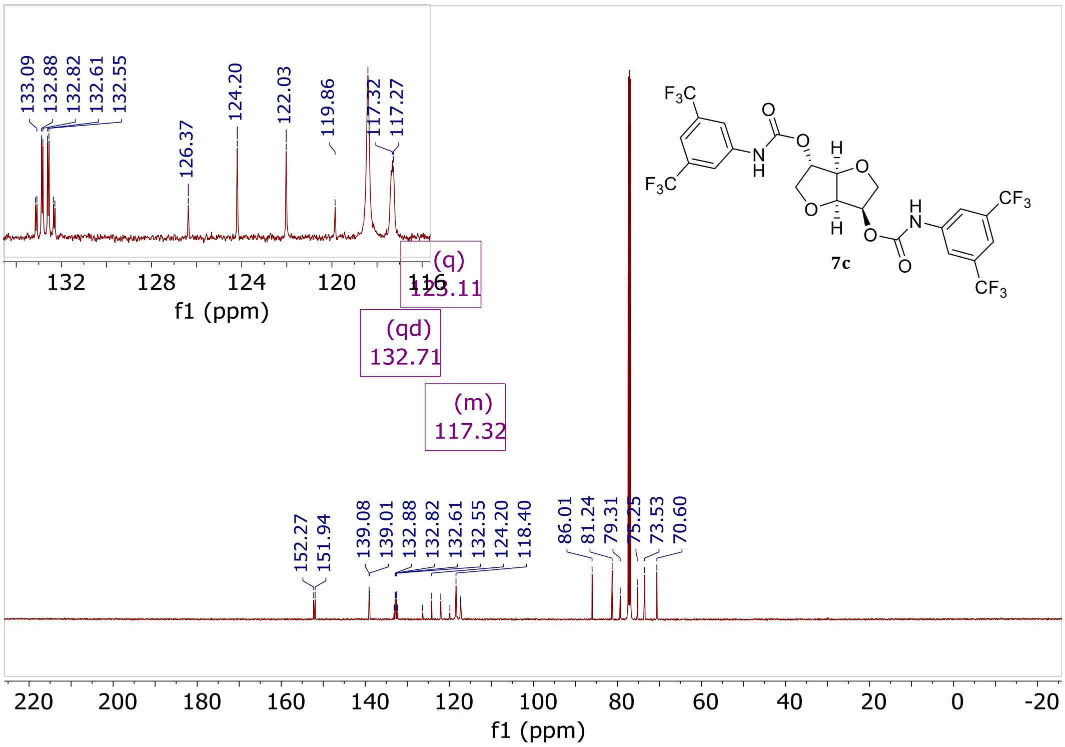

Figure S47. $^{13}C\{^{1}H\}$ NMR (126 MHz, Chloroform-*d*) spectrum of compound **7c**

(3R,3aR,6R,6aR)-hexaydrofuro[3,2-b]furan-3,6-di-(3,5-dimethoxyphenyl)carbamate (4d)

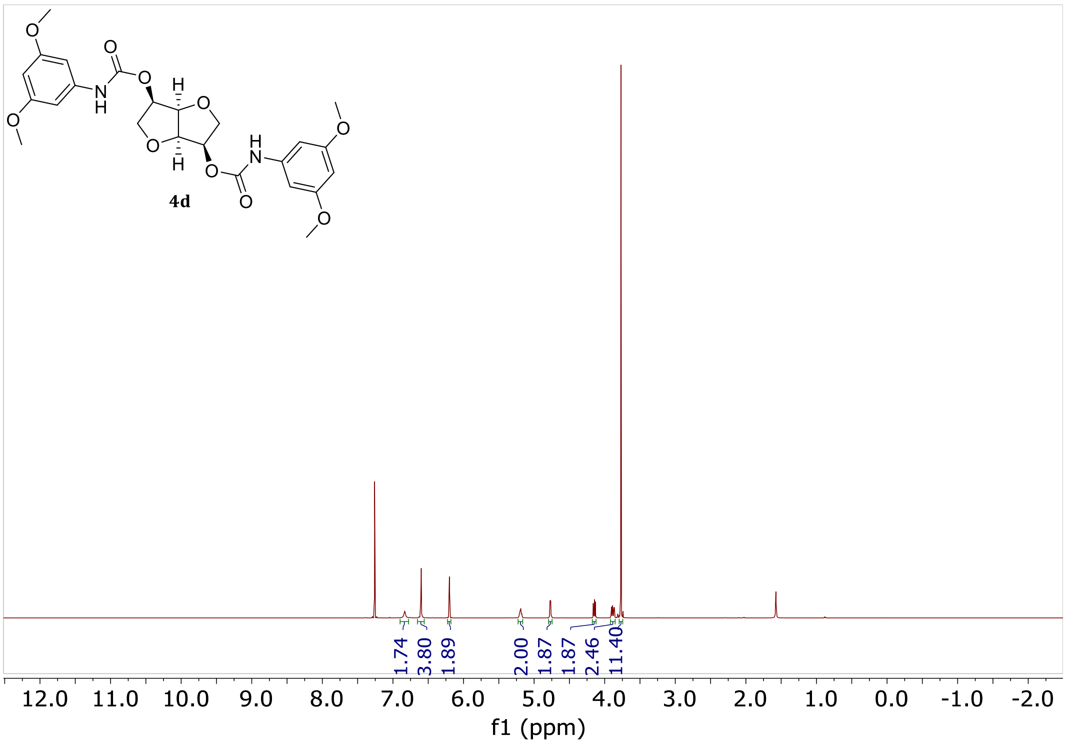

Figure S48. ^{1}H NMR (500 MHz, Chloroform-*d*) spectrum of compound **4d**

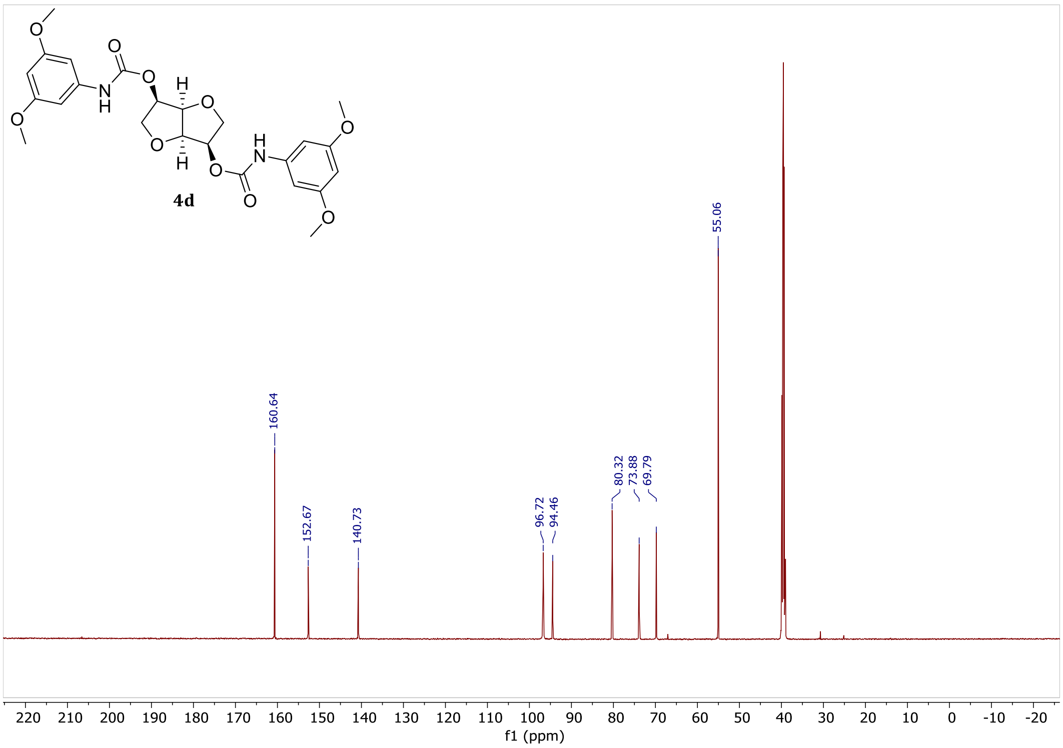

Figure S49. $^{13}C\{^{1}H\}$ NMR (126 MHz, DMSO-d_6) spectrum of compound **4d**

(3R,3aR,6S,6aR)-hexaydrofuro[3,2-b]furan-3,6-di-(3,5-dimethoxyphenyl)carbamate (7d)

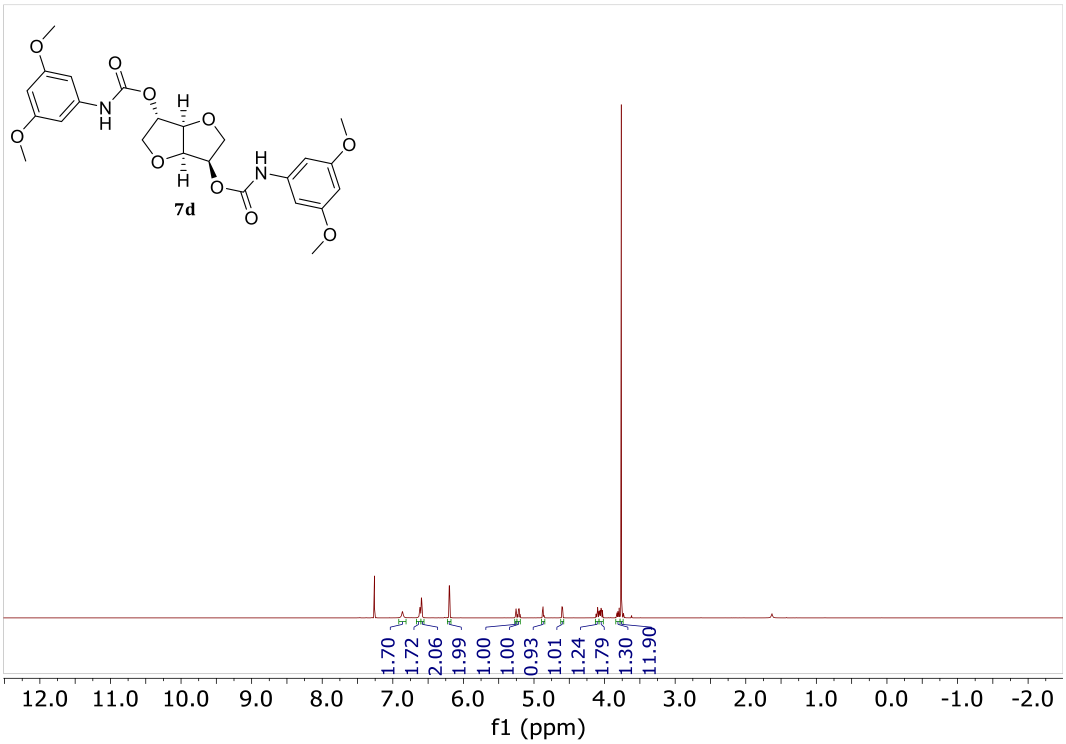

Figure S50. ^{1}H NMR (500 MHz, Chloroform-*d*) spectrum of compound **7d**

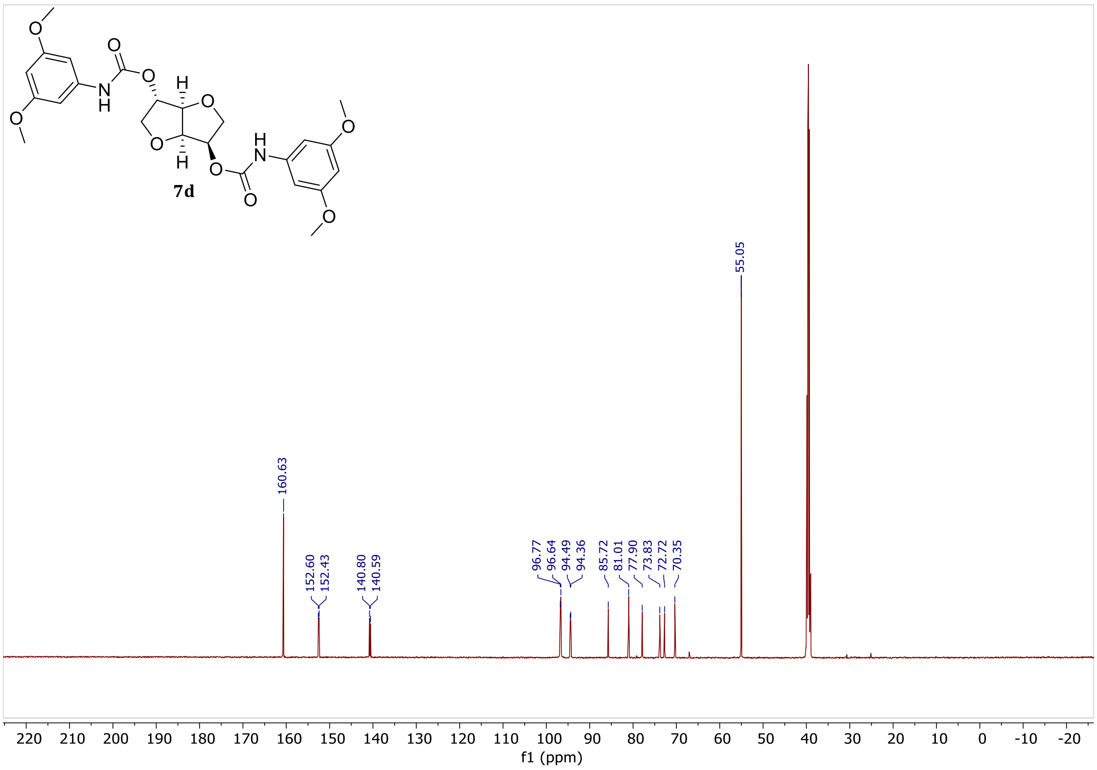

Figure S51. $^{13}C\{^{1}H\}$ NMR (126 MHz, DMSO-d_6) spectrum of compound **7d**

(3R,3aR,6R,6aR)-hexaydrofuro[3,2-b]furan-3,6-di-(p-toluensulfonyl)carbamate (4e)

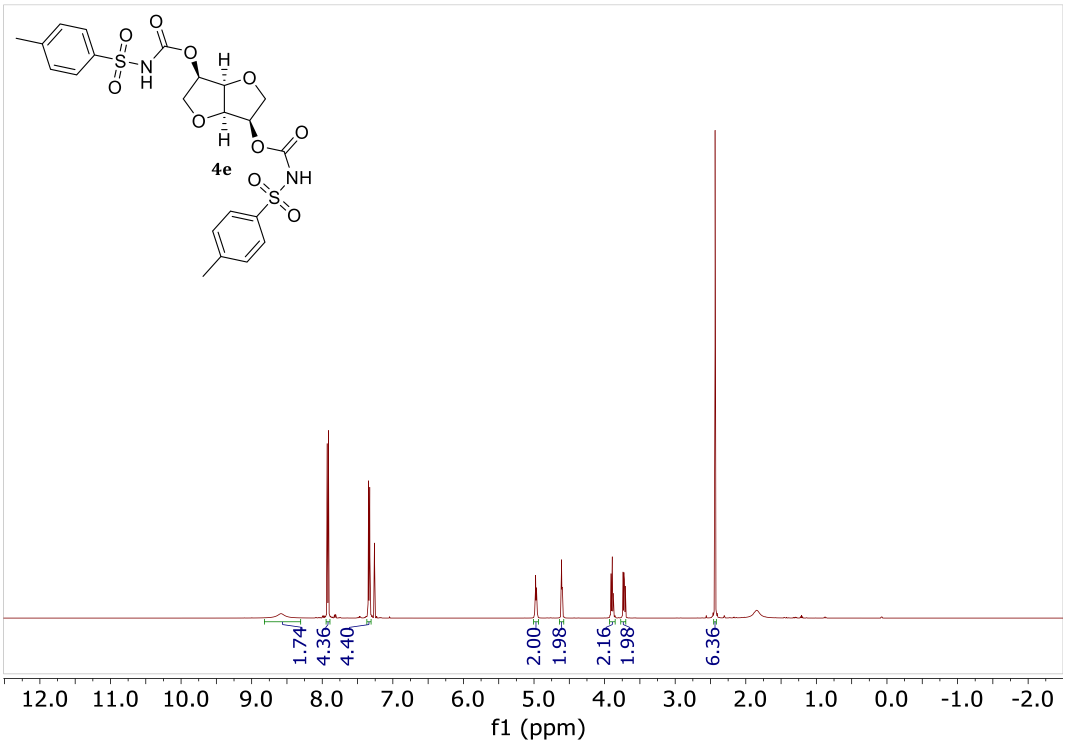

Figure S52. ^{1}H NMR (500 MHz, Chloroform-*d*) spectrum of compound **4e**

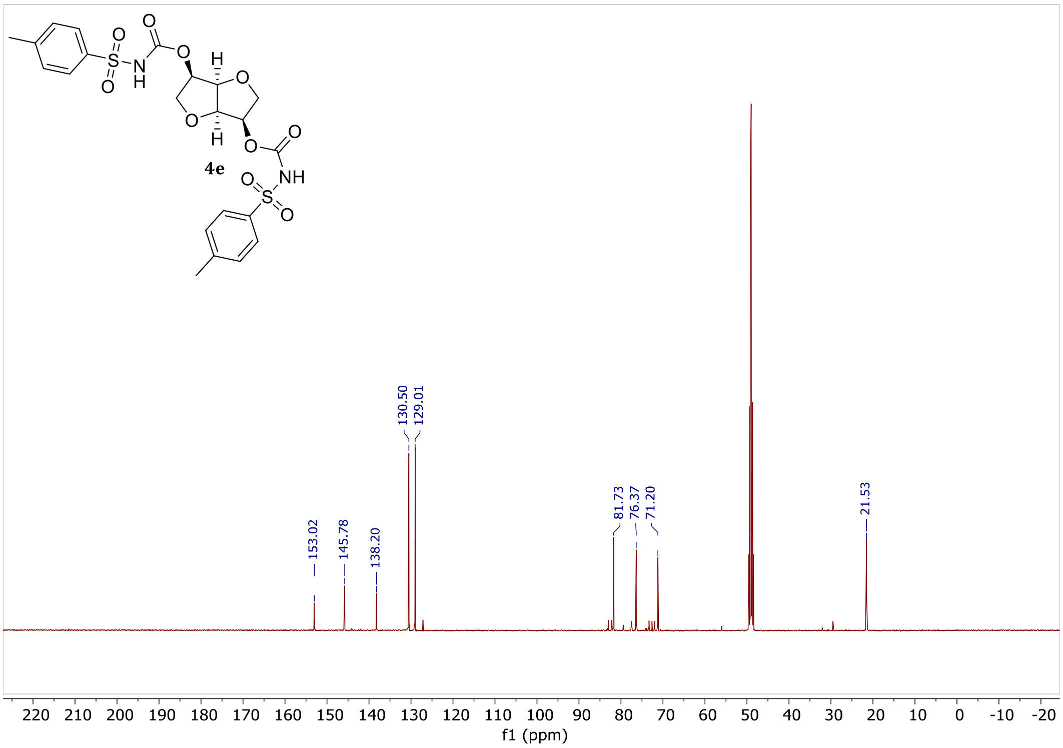

Figure S53. $^{13}C\{^{1}H\}$ NMR (126 MHz, Methanol-d_4) spectrum of compound **4e**

(3R,3aR,6S,6aR)-hexaydrofuro[3,2-b]furan-3,6-di-(p-toluensulfonyl)carbamoyl-3-ol (7e)

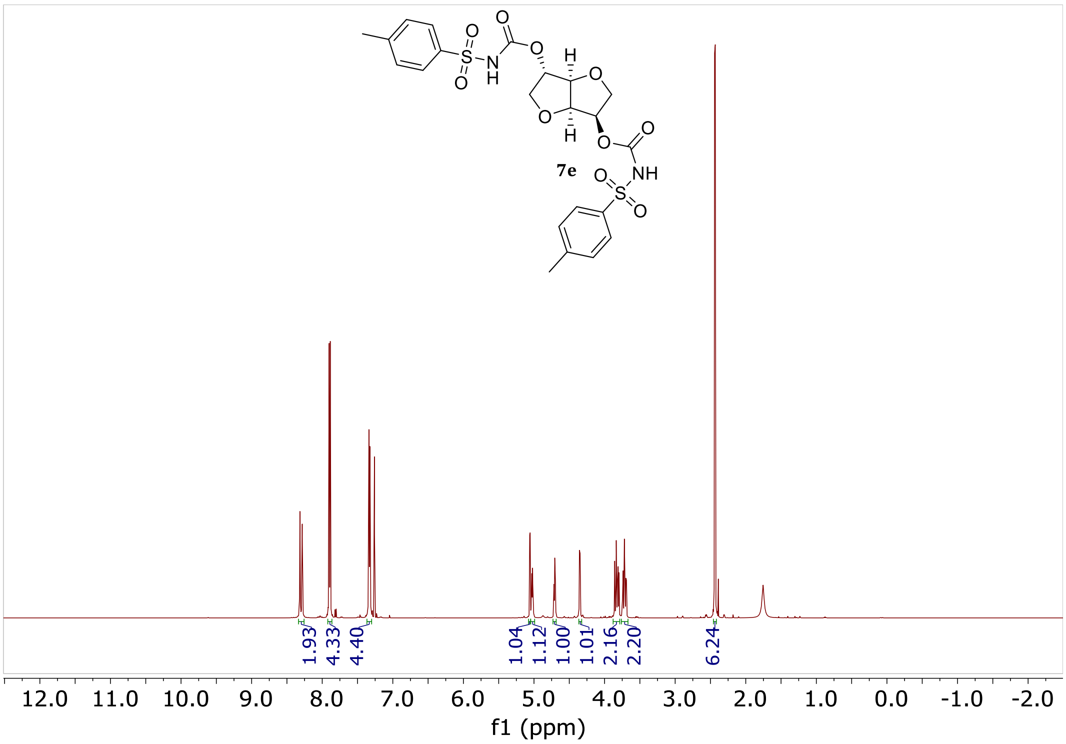

Figure S54. ^{1}H NMR (500 MHz, Chloroform-*d*) spectrum of compound **7e**

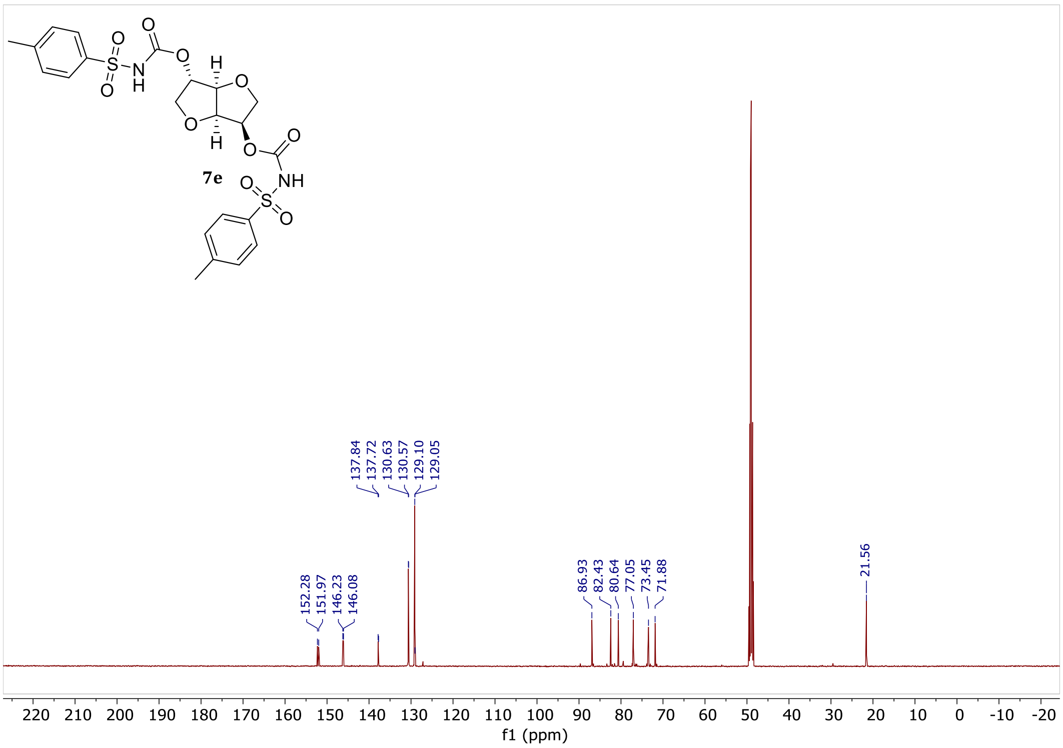

Figure S55. $^{13}C\{^{1}H\}$ NMR (126 MHz, Methanol-d_4) spectrum of compound **7e**

N-3,5-dinitrobenzoylphenylglycine (9’)

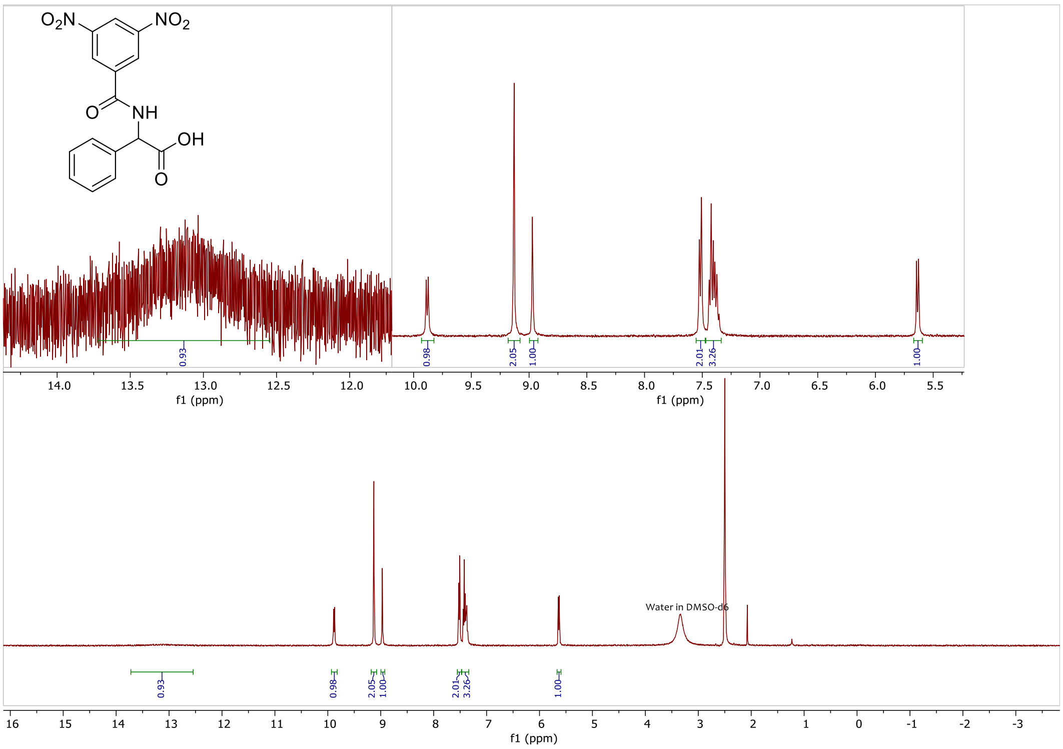

Figure S56. ^{1}H NMR (500 MHz, DMSO-*d*$_6$) spectrum of compounds **9’**

N-3,5-dinitrobenzoylphenylglycine methyl ester (9)

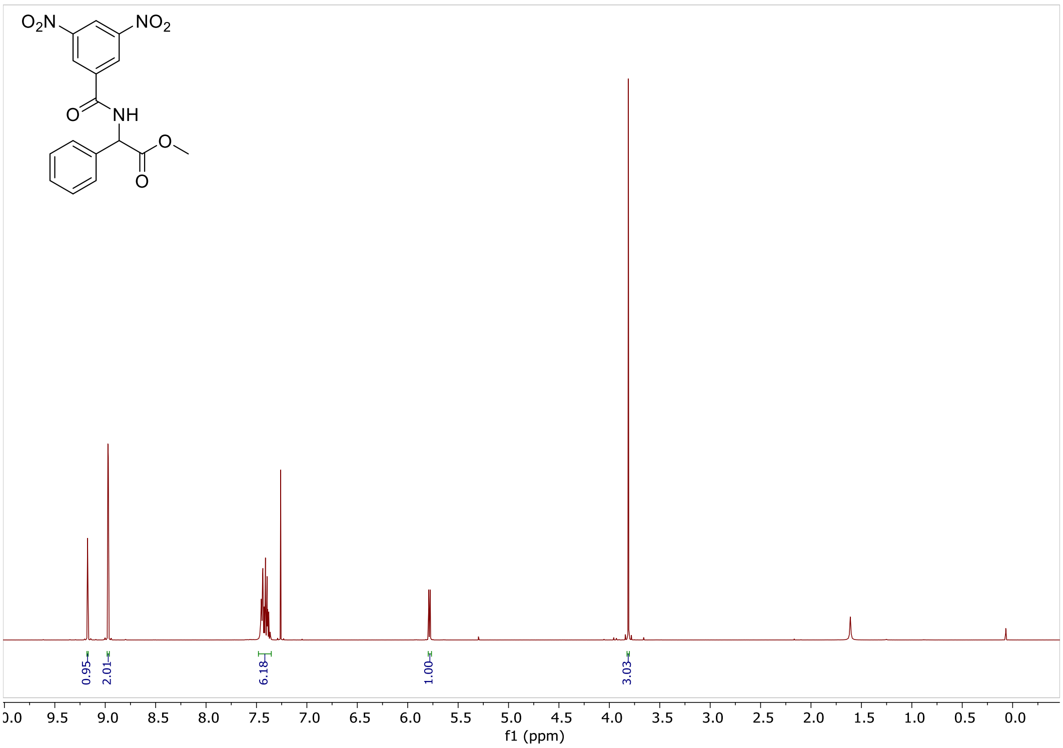

Figure S57. ^{1}H NMR (500 MHz, Chloroform-*d*) spectrum of compound **9**

N-3,5-dimethoxybenzoylphenylglycine (10’)

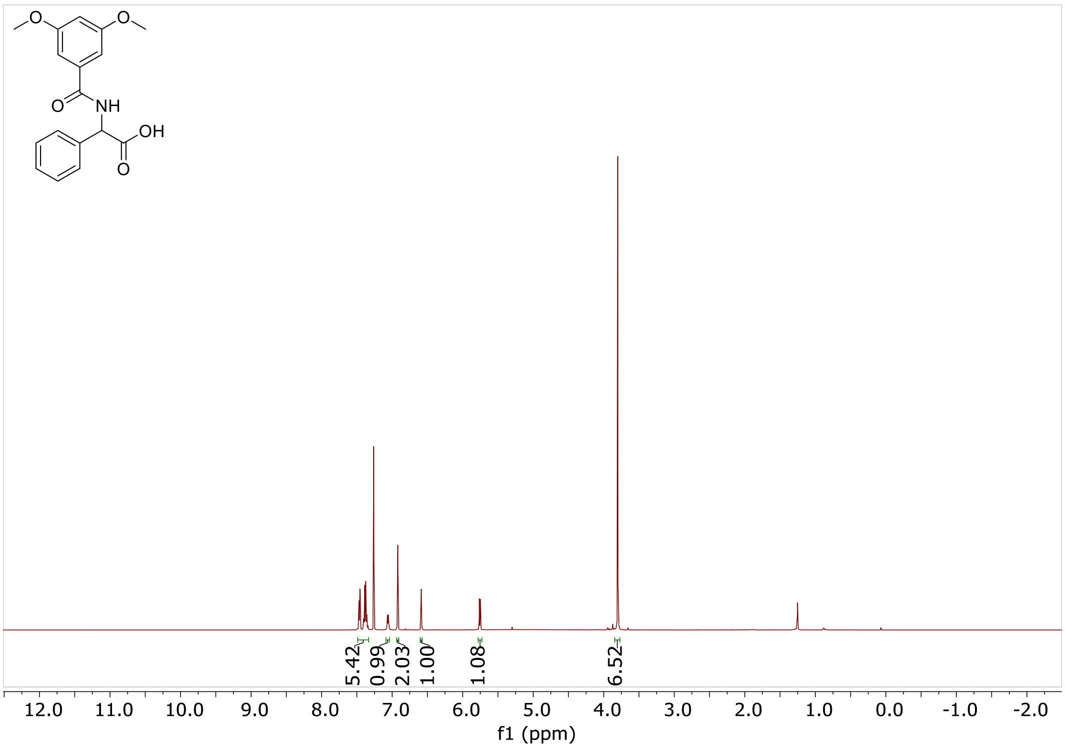

Figure S58. ^{1}H NMR (500 MHz, Chloroform-*d*) spectrum of compound **10’**

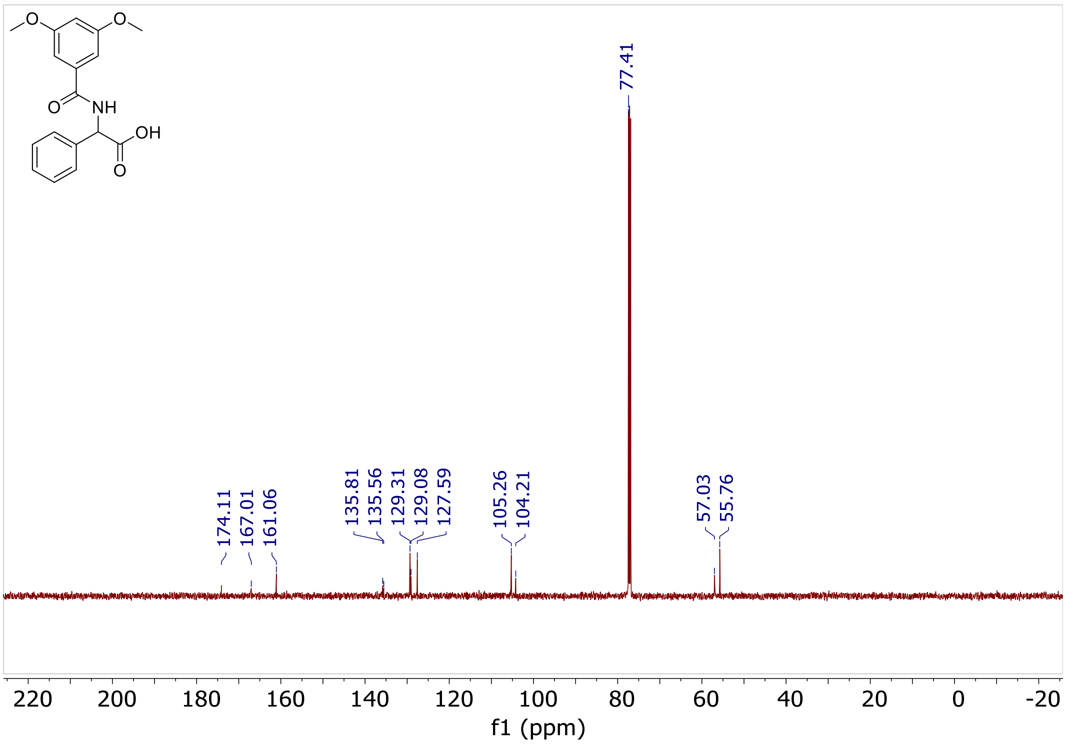

Figure S59. $^{13}C\{^{1}H\}$ NMR (126 MHz, Chloroform-*d*) spectrum of compound **10’**

N-3,5-dimethoxybenzoylphenylglycine methyl ester (10)

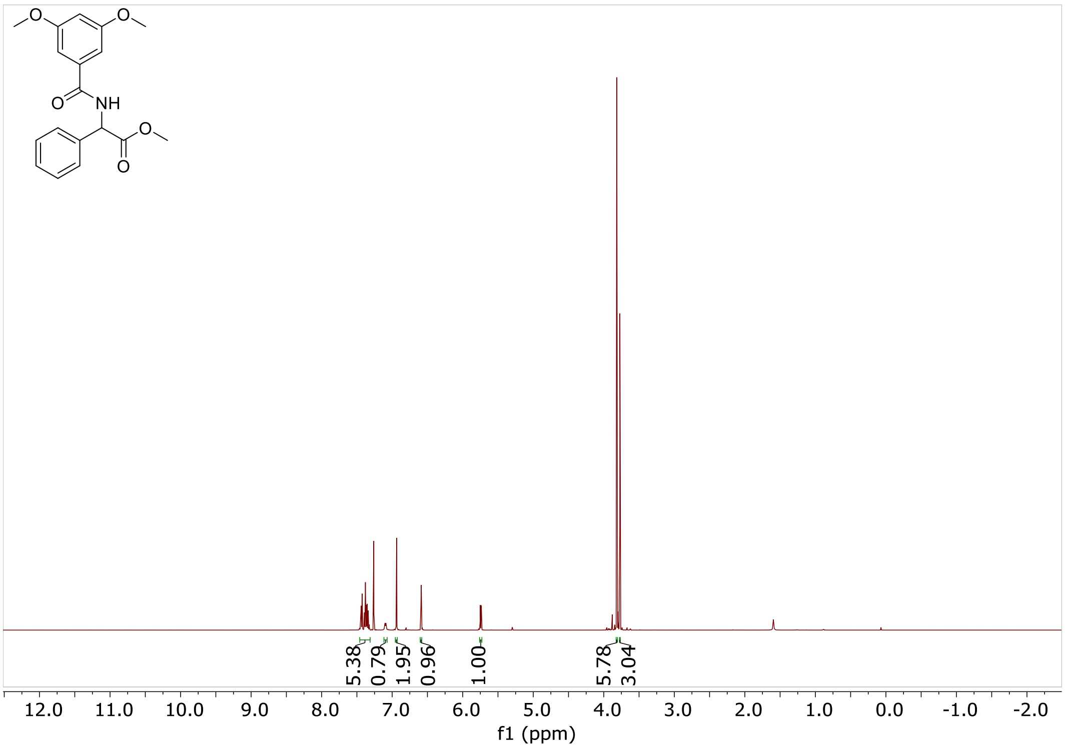

Figure S60. ^{1}H NMR (500 MHz, Chloroform-*d*) spectrum of compound **10**

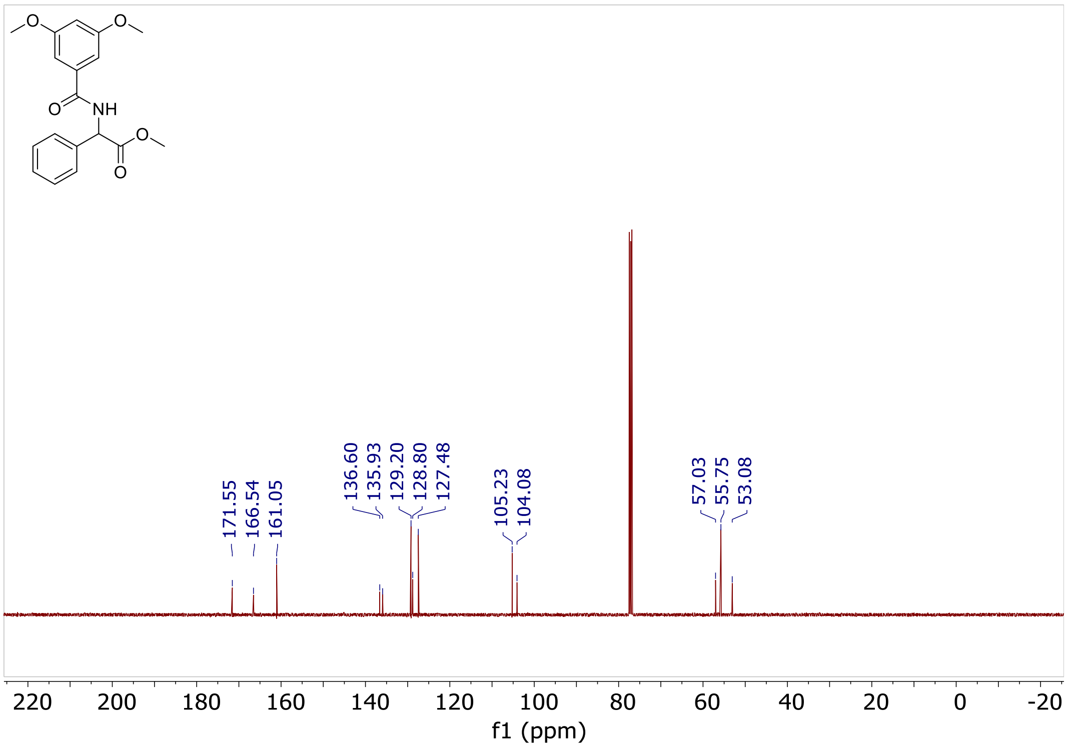

Figure S61. $^{13}C\{^{1}H\}$ NMR (126 MHz, Chloroform-*d*) spectrum of compound **10**

*Enantiodiscrimination tests on compound **9** employing CSAs **3-7***

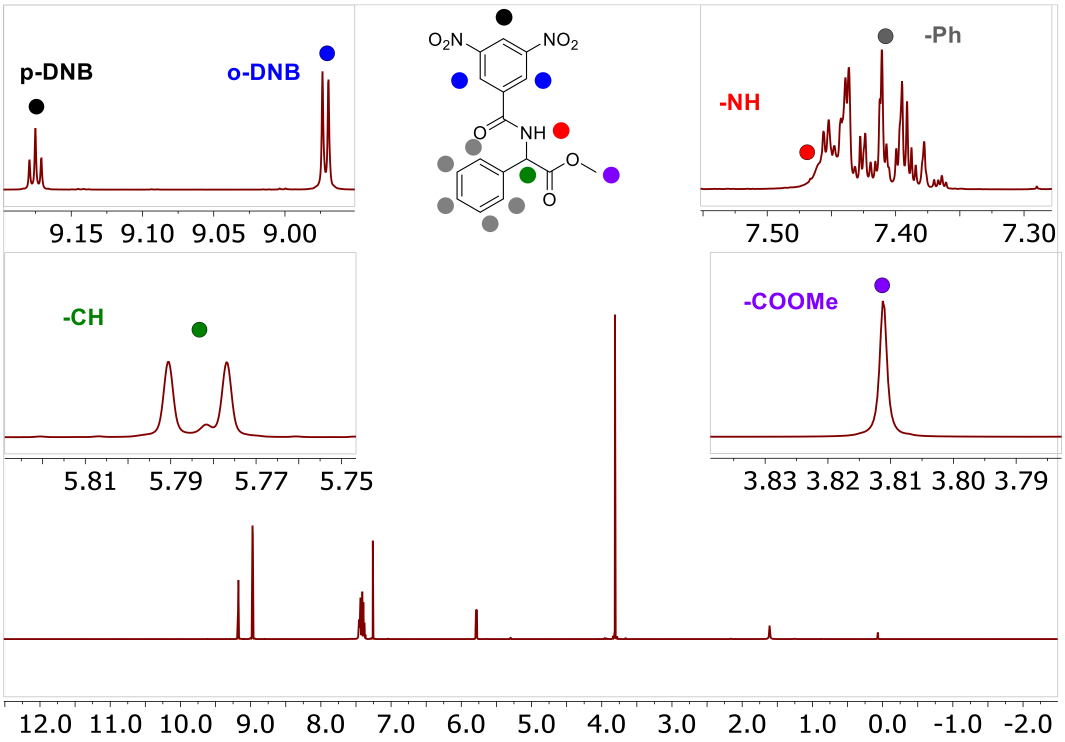

Figure S62. ^{1}H NMR (500 MHz, Chloroform-*d*, 21 °C) spectrum of *rac*-**9** (30 mM).

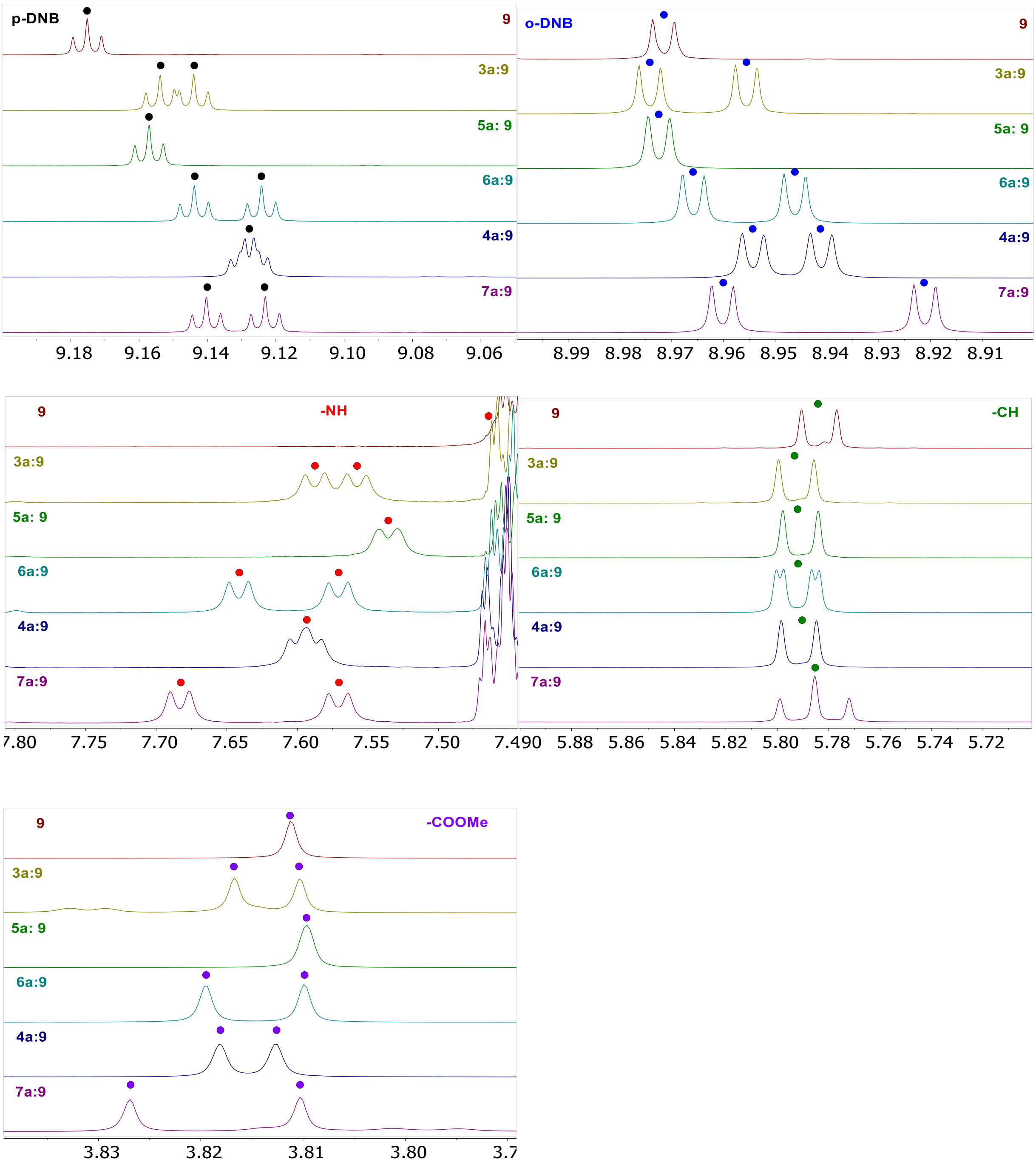

Figure S63. ^{1}H NMR (500 MHz, Chloroform-*d*, 21 °C) spectra of rac-**9** (30 mM) in the presence of 1 equivalent of CSA **3a**-**7a**. Only selected spectral regions containing the signals of **9** are reported.

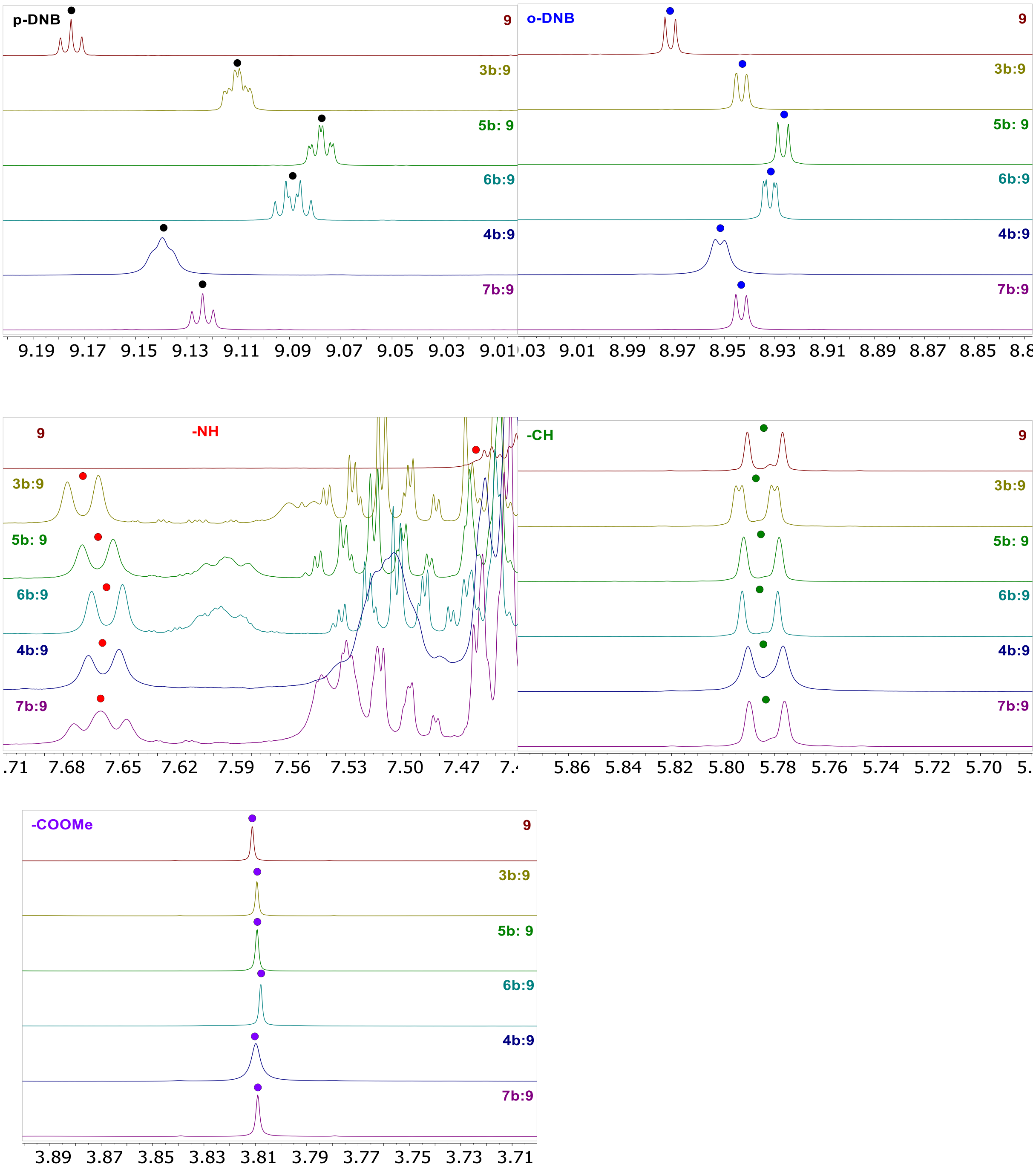

Figure S64. ^{1}H NMR (500 MHz, Chloroform-*d*, 21 °C) spectra of *rac*-**9** (30 mM) in the presence of 1 equivalent of CSA **3b**-**7b**. Only selected spectral regions containing the signals of **9** are reported.

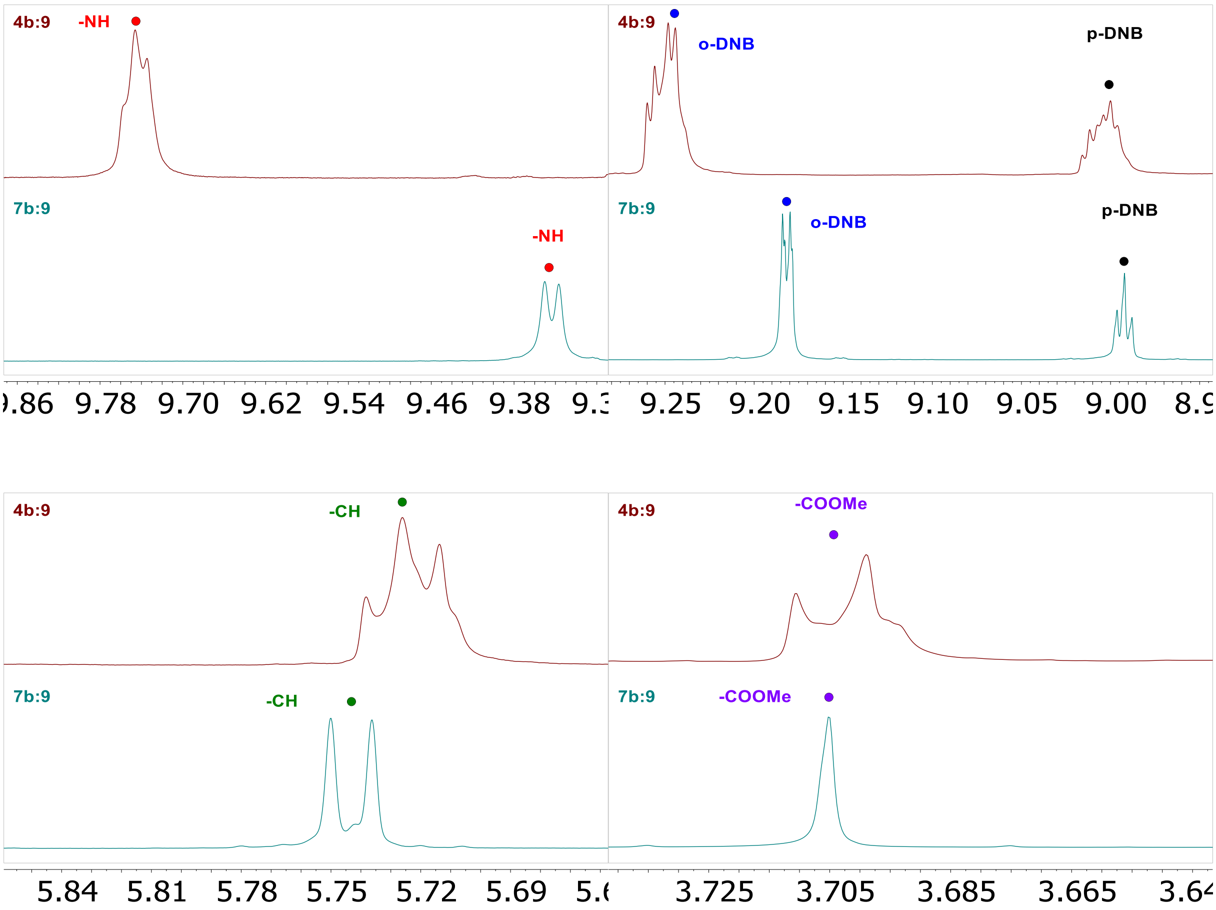

Figure S65. ^{1}H NMR (500 MHz, Chloroform-*d*, 21 °C) spectra of *rac*-**9** (30 mM) in the presence of 1 equivalent of CSA **4b** or **7b** after addition of DMSO-d_6 to have complete dissolution. Red line: spectra of an equimolar mixture of **4b/9** recorded adding 150 μL of DMSO-d_6. Light blue line: spectra of an equimolar mixture of **7b/9** recorded adding 30 μL of DMSO-d_6. Only selected spectral regions containing the signals of **9** are reported.

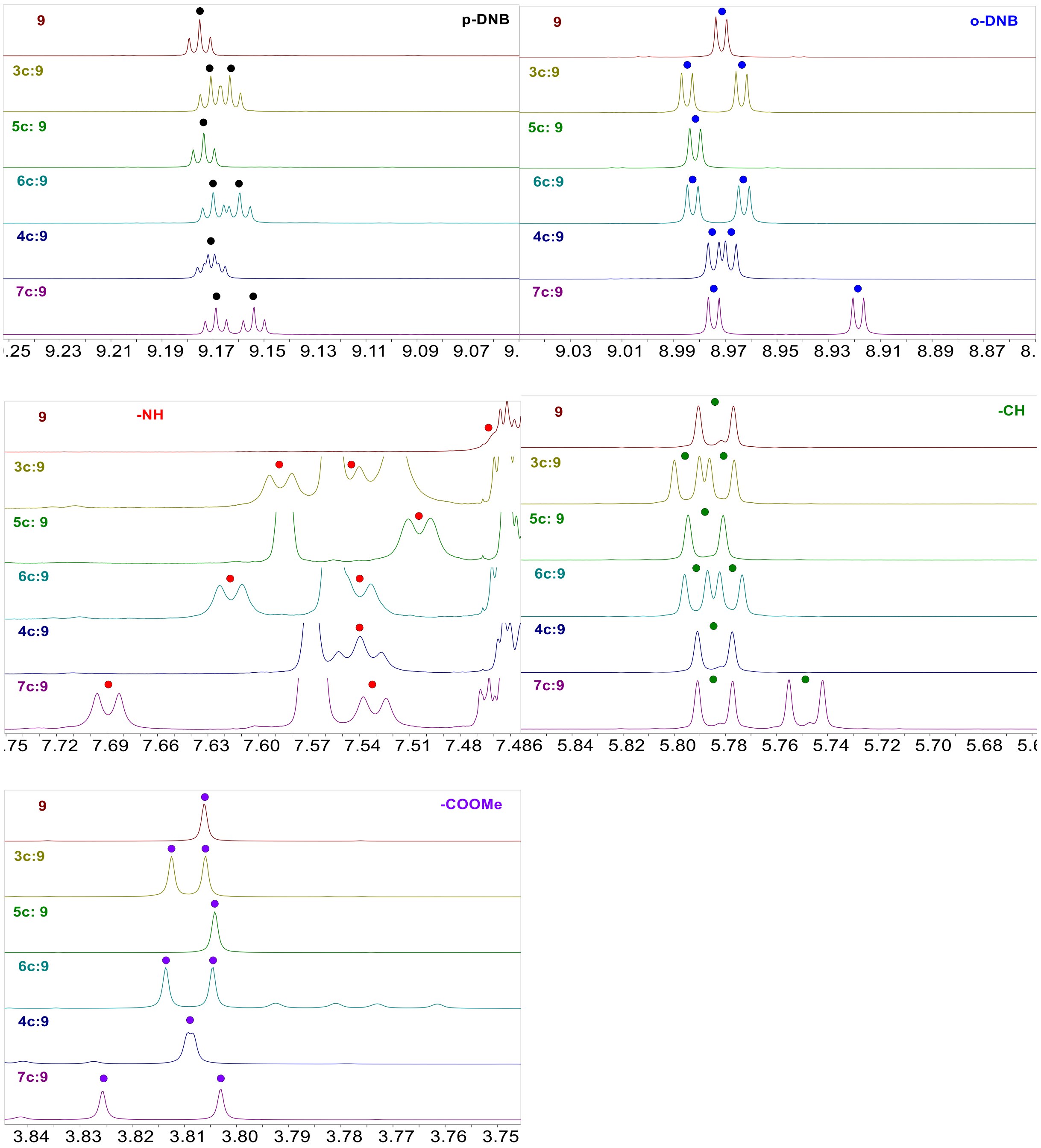

Figure S66. ^{1}H NMR (500 MHz, Chloroform-*d*, 21 °C) spectra of *rac*-**9** (30 mM) in the presence of 1 equivalent of CSA **3c**-**7c**. Only selected spectral regions containing the signals of **9** are reported.

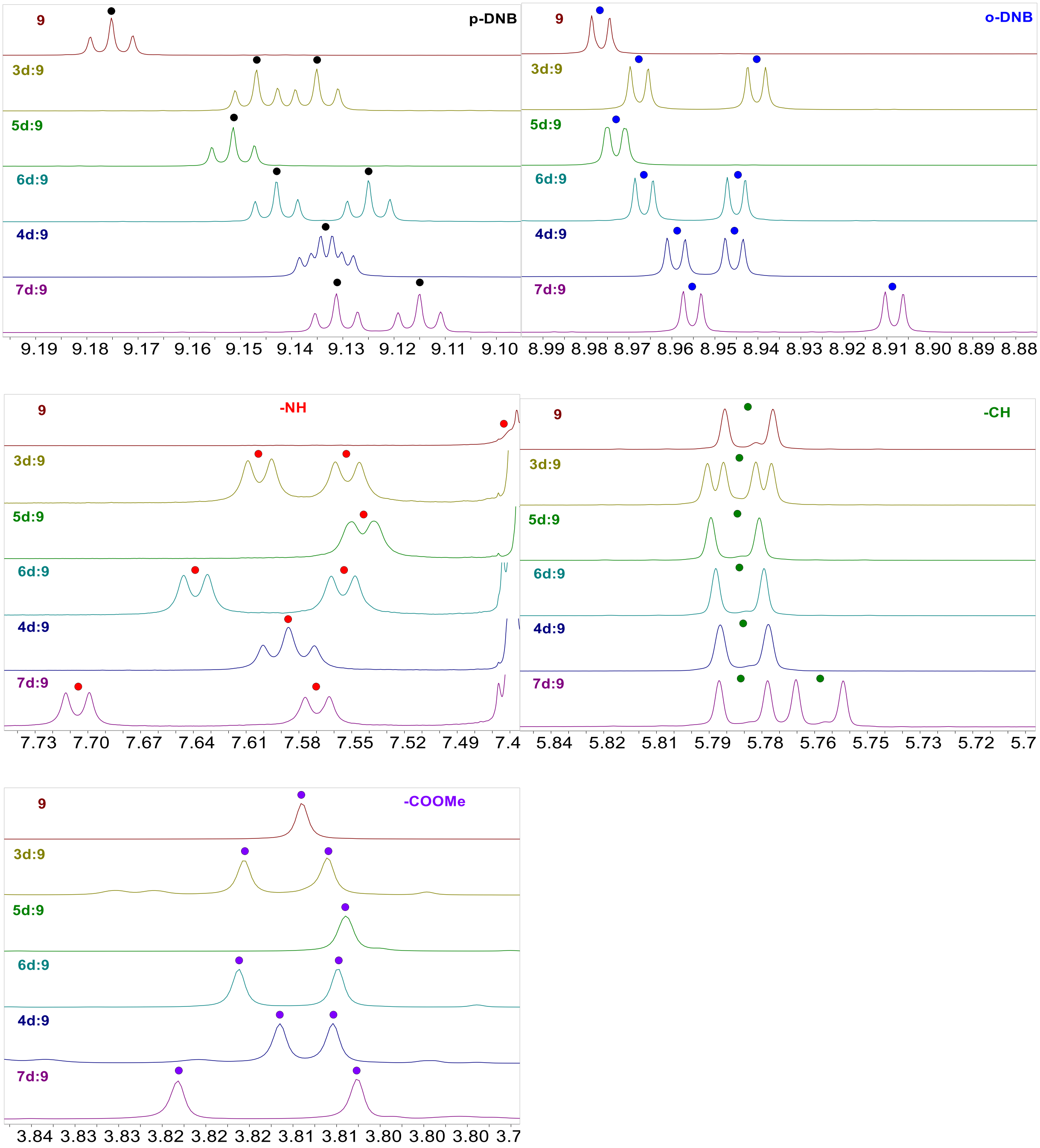

Figure S67. ^{1}H NMR (500 MHz, Chloroform-*d*, 21 °C) spectra of *rac*-**9** (30 mM) in the presence of 1 equivalent of CSA **3d-7d**. Only selected spectral regions containing the signals of **9** are reported.

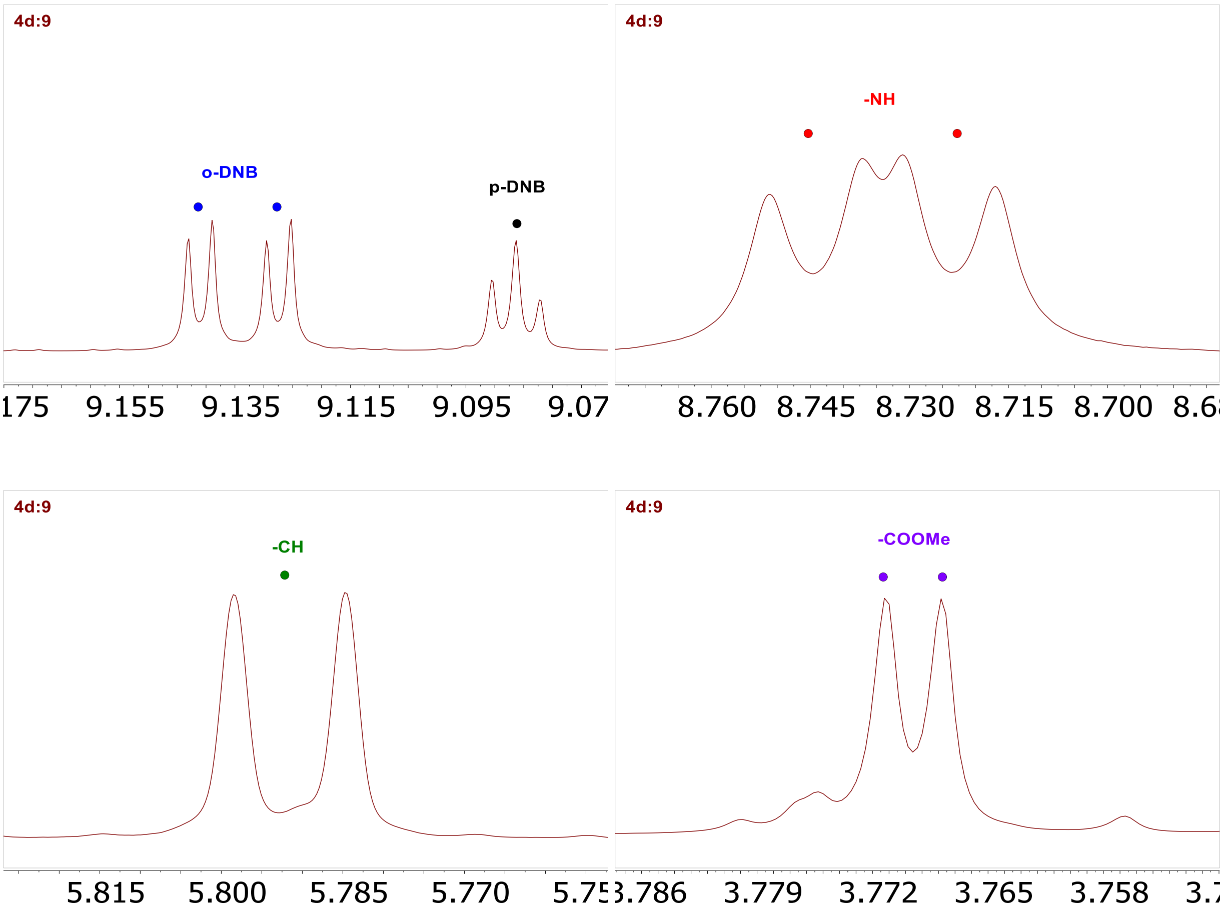

Figure S68. ^{1}H NMR (500 MHz, Chloroform-*d*, 21 °C) spectrum of *rac*-**9** (30 mM) in the presence of 1 equivalent of CSA **4d** after addition of 10 μL of DMSO-d_6 to have complete dissolution. Only selected spectral regions containing the signals of **9** are reported.

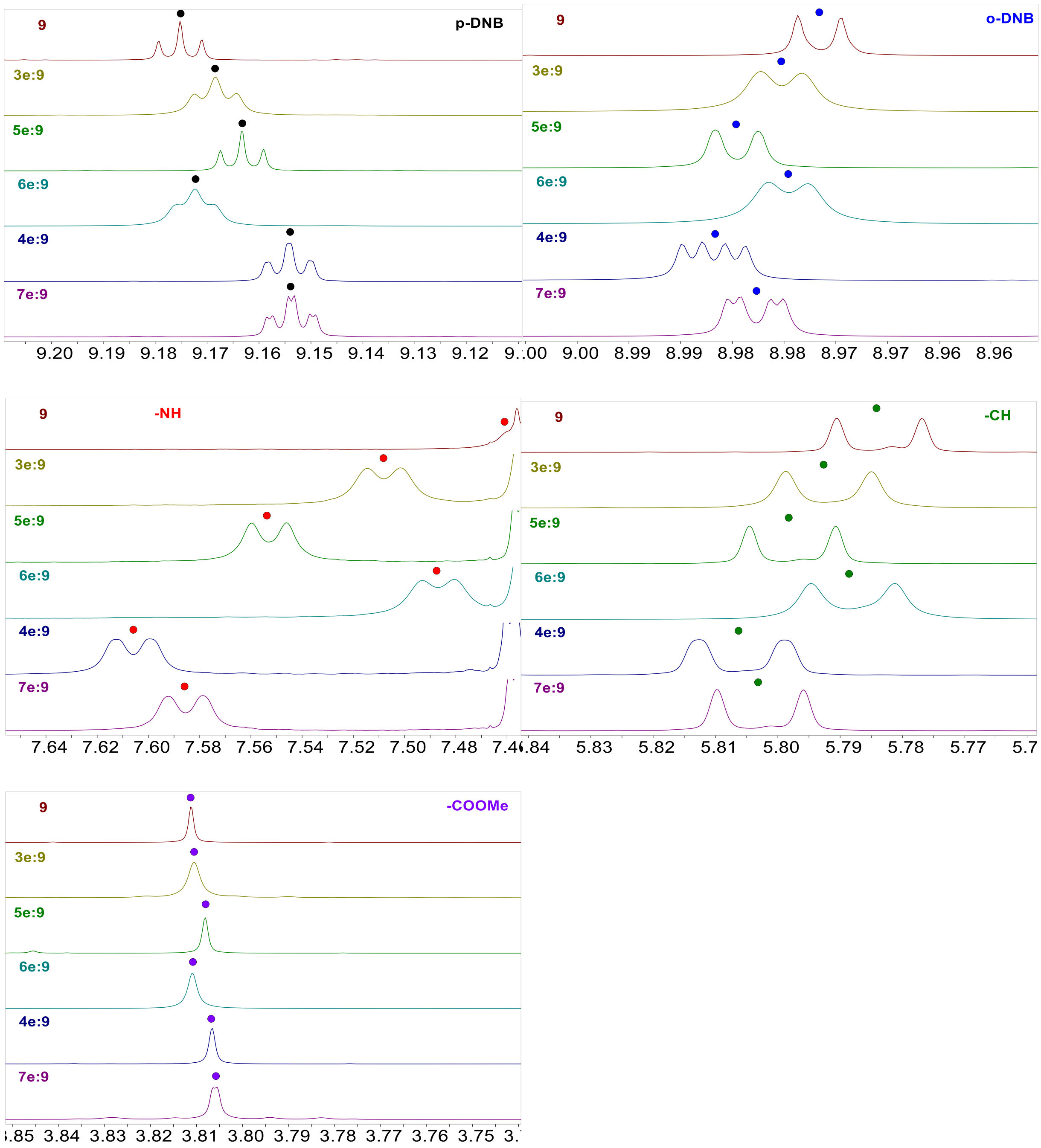

Figure S69. ^{1}H NMR (500 MHz, Chloroform-*d*, 21 °C) spectra of *rac*-**9** (30 mM) in the presence of 1 equivalent of CSA **3e**-**7e**. Only selected spectral regions containing the signals of **9** are reported.

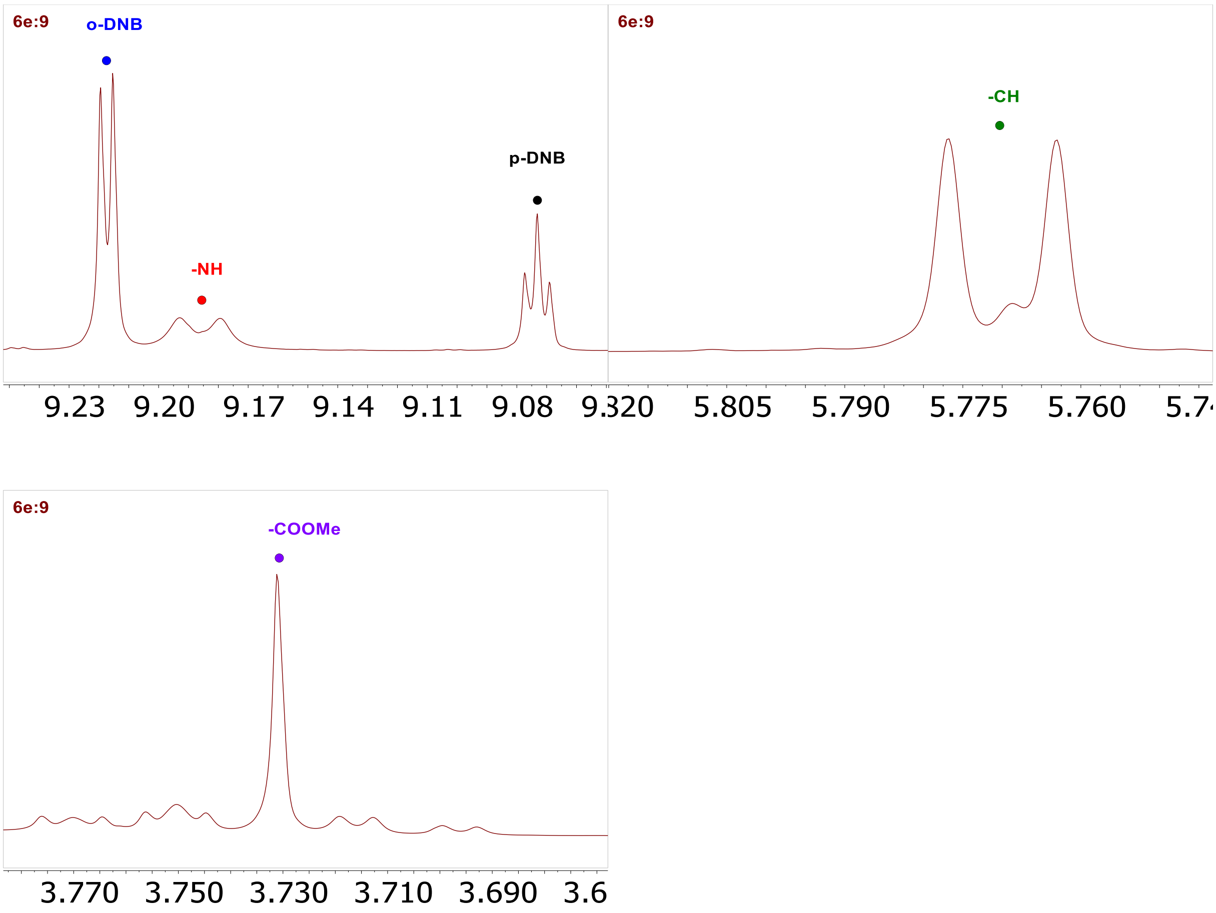

Figure S70. ^{1}H NMR (500 MHz, Chloroform-*d*, 21 °C) spectrum of *rac*-**9** (30 mM) in the presence of 1 equivalent of CSA **6e** after addition of 30 μL of DMSO-d_6 to have complete dissolution. Only selected spectral regions containing the signals of **9** are reported.

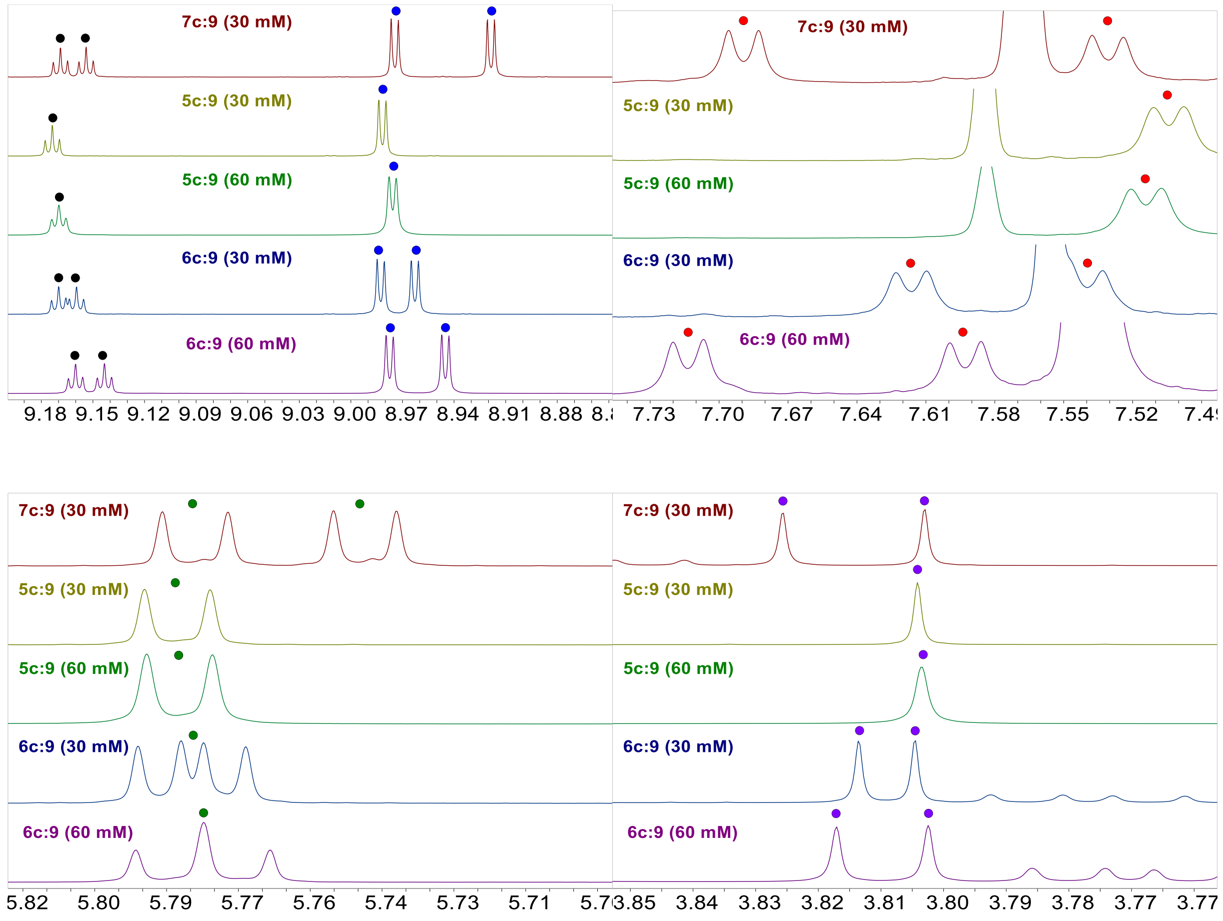

Figure S71. ^{1}H NMR (500 MHz, Chloroform-*d*, 21 °C) spectra of *rac*-**9** (30 mM) in the presence of: 1 equivalent of CSA **7c** (red line), 1 equivalent of CSA **5c** (yellow line), 2 equivalents of CSA **5c** (green line), 1 equivalent of CSA **6c** (blue line), 2 equivalents of CSA **6c** (purple line). Only selected spectral regions containing the signals of **9** are reported.

Enantiodiscrimination tests on compounds ***10-12*** *employing CSA* ***7c***

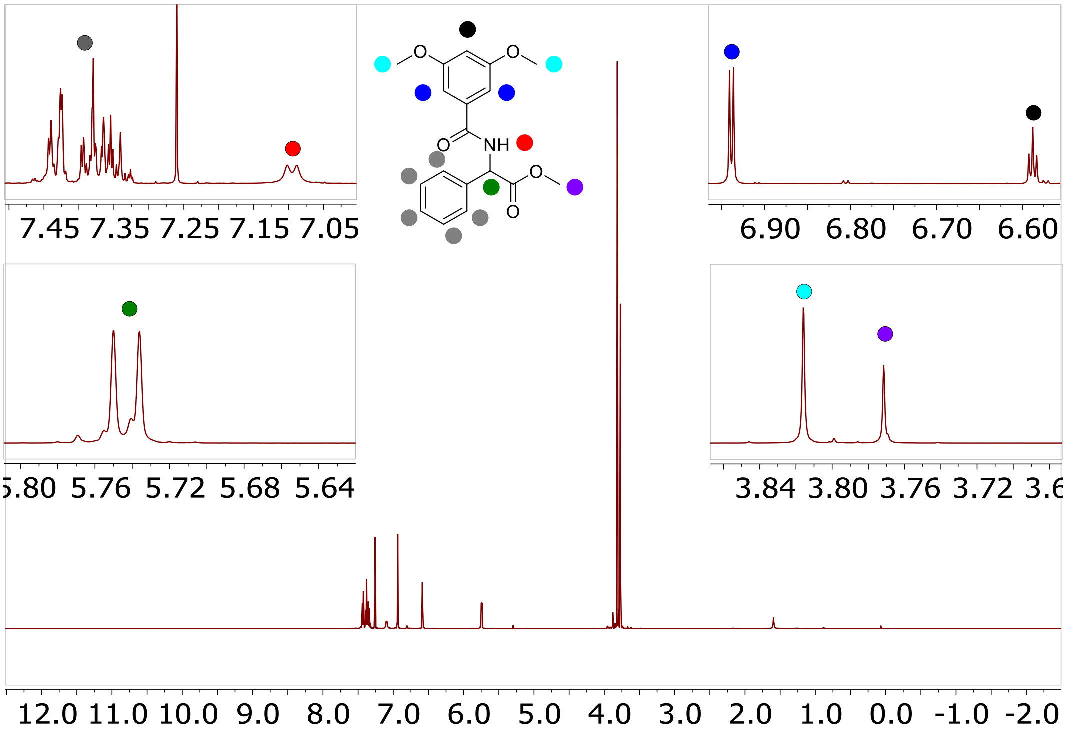

Figure S72. ^{1}H NMR (500 MHz, Chloroform-*d*, 21 °C) spectrum of pure *rac*-**10** (30 mM).

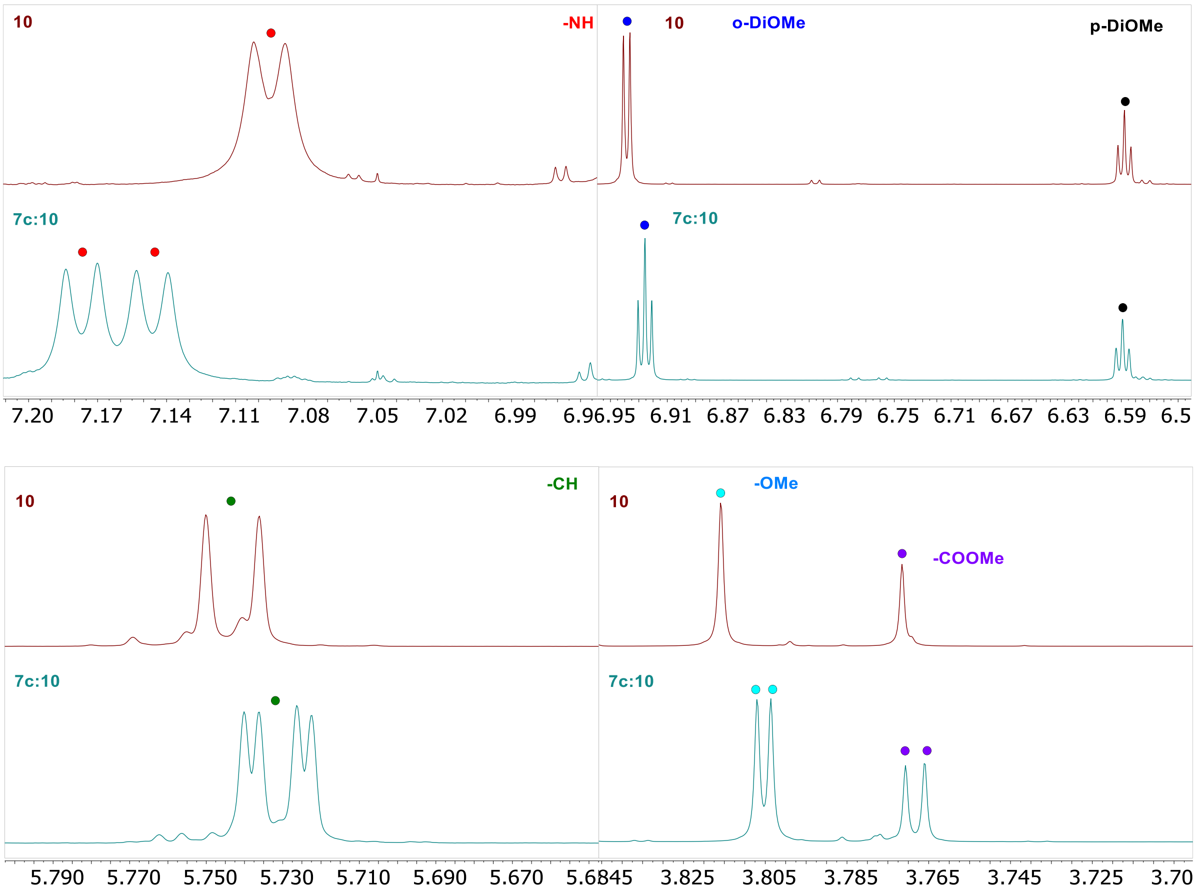

Figure S73. ^{1}H NMR (500 MHz, Chloroform-*d*, 21 °C) spectra of: pure *rac*-**10** (30 mM) (red line) and *rac*-**10** (30mM) in the presence of 1 equivalent of CSA **7c** (light blue line). Only selected spectral regions containing the signals of **10** are reported.

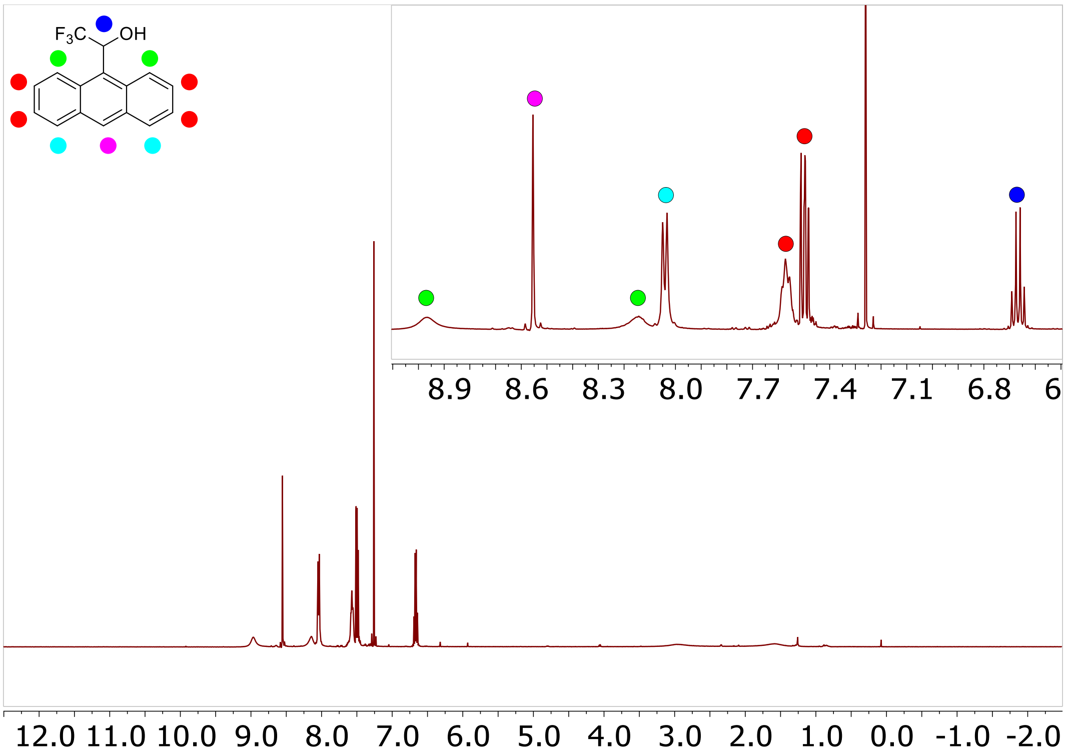

Figure S 74. ^{1}H NMR (500 MHz, Chloroform-*d*, 21 °C) spectra of pure *rac*-**11** (30 mM).

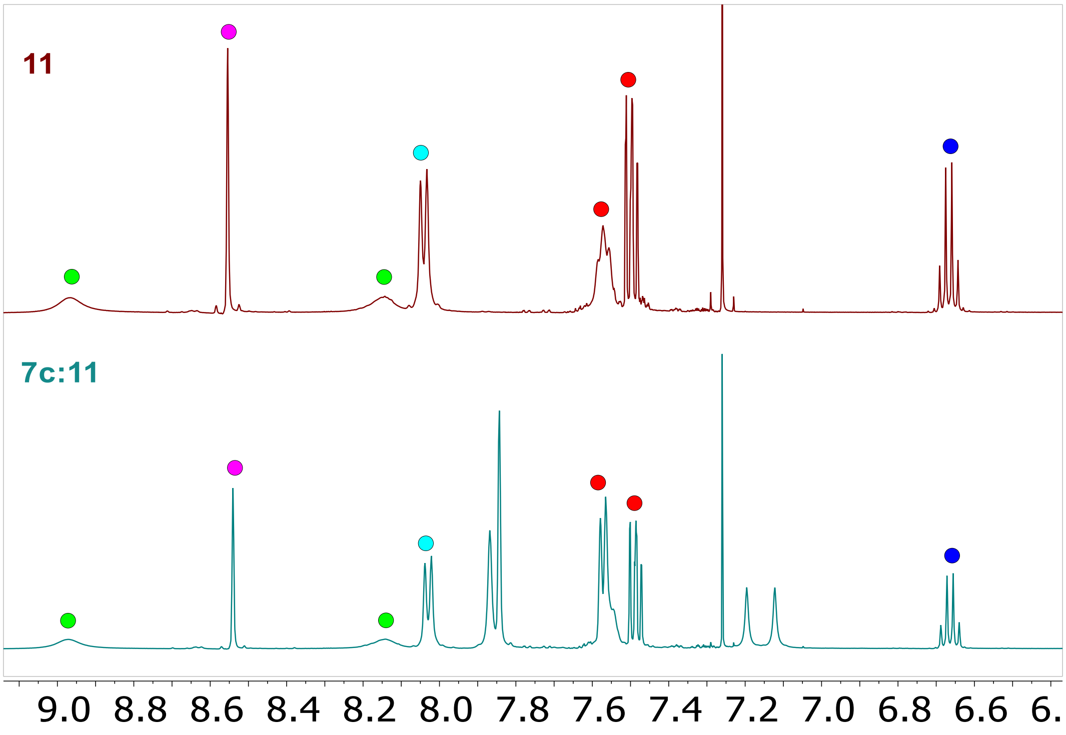

Figure S75. ^{1}H NMR (500 MHz, Chloroform-*d*, 21 °C) spectra of: pure *rac*-**11** (30 mM) (red line) and *rac*-**11** (30mM) in the presence of 1 equivalent of CSA **7c** (light blue line). Only selected spectral regions containing the signals of **11** are reported.

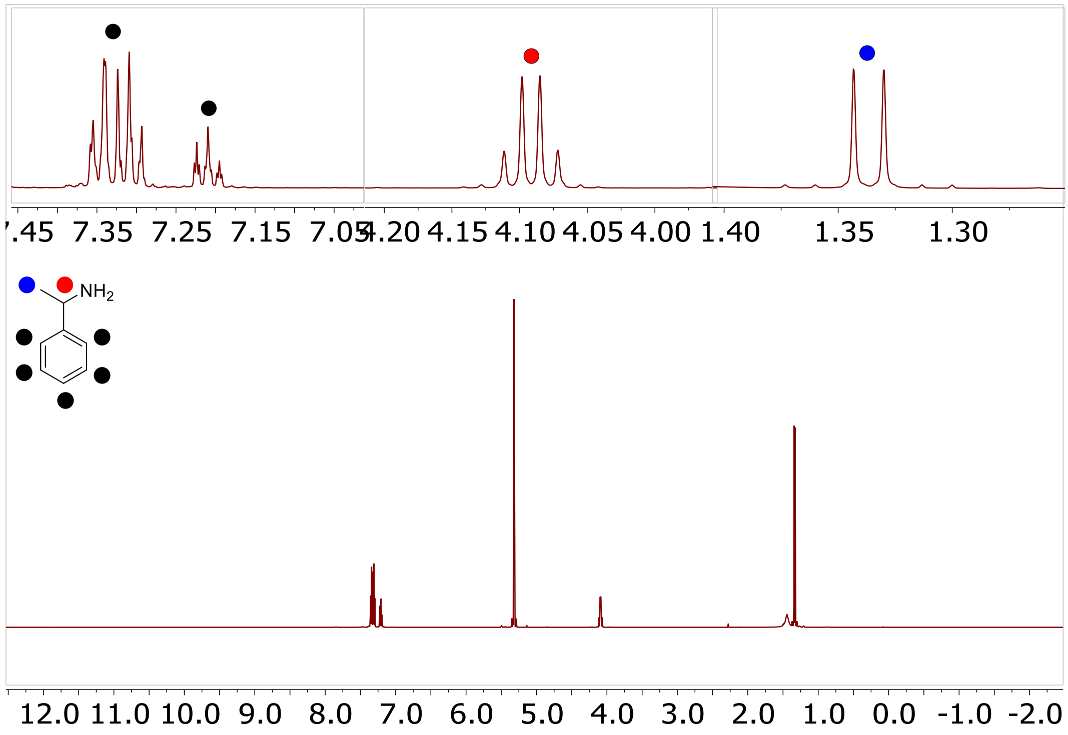

Figure S76. ^{1}H NMR (500 MHz, Methylene chloride-d_2, 21 °C) spectrum of pure *rac*-**12** (30 mM).

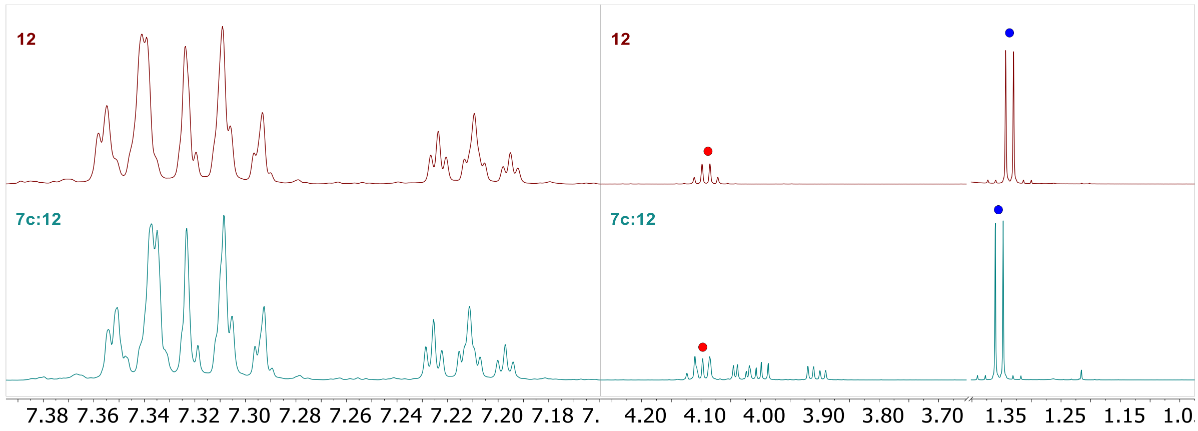

Figure S77. ^{1}H NMR (500 MHz, Methylene chloride-d_2, 21 °C) spectra of: pure *rac*-**12** (30 mM) (red line) and *rac*-**12** (30mM) in the presence of 1 equivalent of CSA **7c** (light blue line). Only selected spectral regions containing the signals of **12** are reported.

*Enantiodiscrimination tests on compound **12** employing CSAs **3e**-**7e***

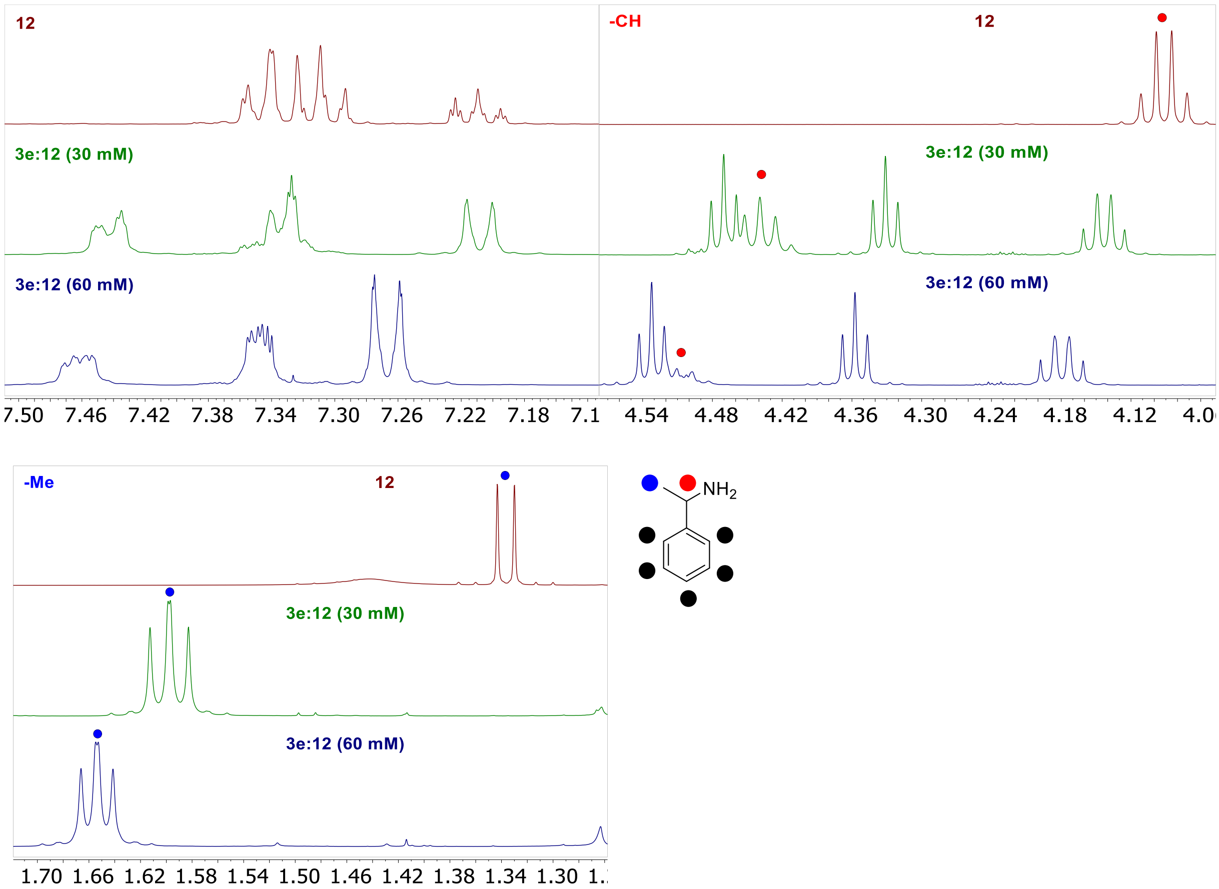

Figure S78. ^{1}H NMR (500 MHz, Methylene Chloride-*d*$_2$, 21 °C) spectra of: pure *rac*-**12** (30 mM) (red line); *rac*-**12** (30 mM) in the presence of 1 equivalent of CSA **3e**; *rac*-**12** (30 mM) in the presence of 2 equivalents of CSA **3e**. Only selected spectral regions containing the signals of **12** are reported.

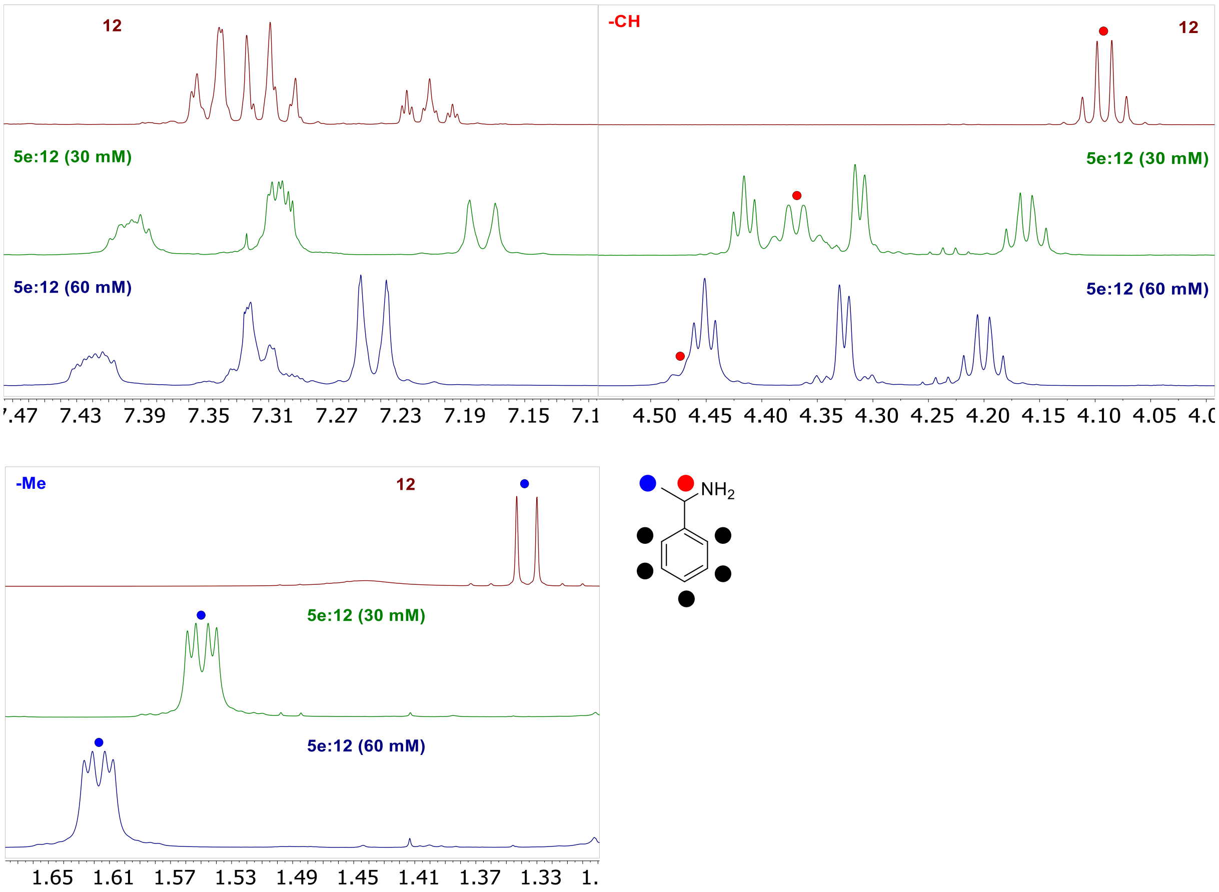

Figure S79. ^{1}H NMR (500 MHz, Methylene Chloride-d_2, 21 °C) spectra of: pure *rac*-**12** (30 mM) (red line); *rac*-**12** (30 mM) in the presence of 1 equivalent of CSA **5e**; *rac*-**12** (30 mM) in the presence of 2 equivalents of CSA **5e**. Only selected spectral regions containing the signals of **12** are reported.

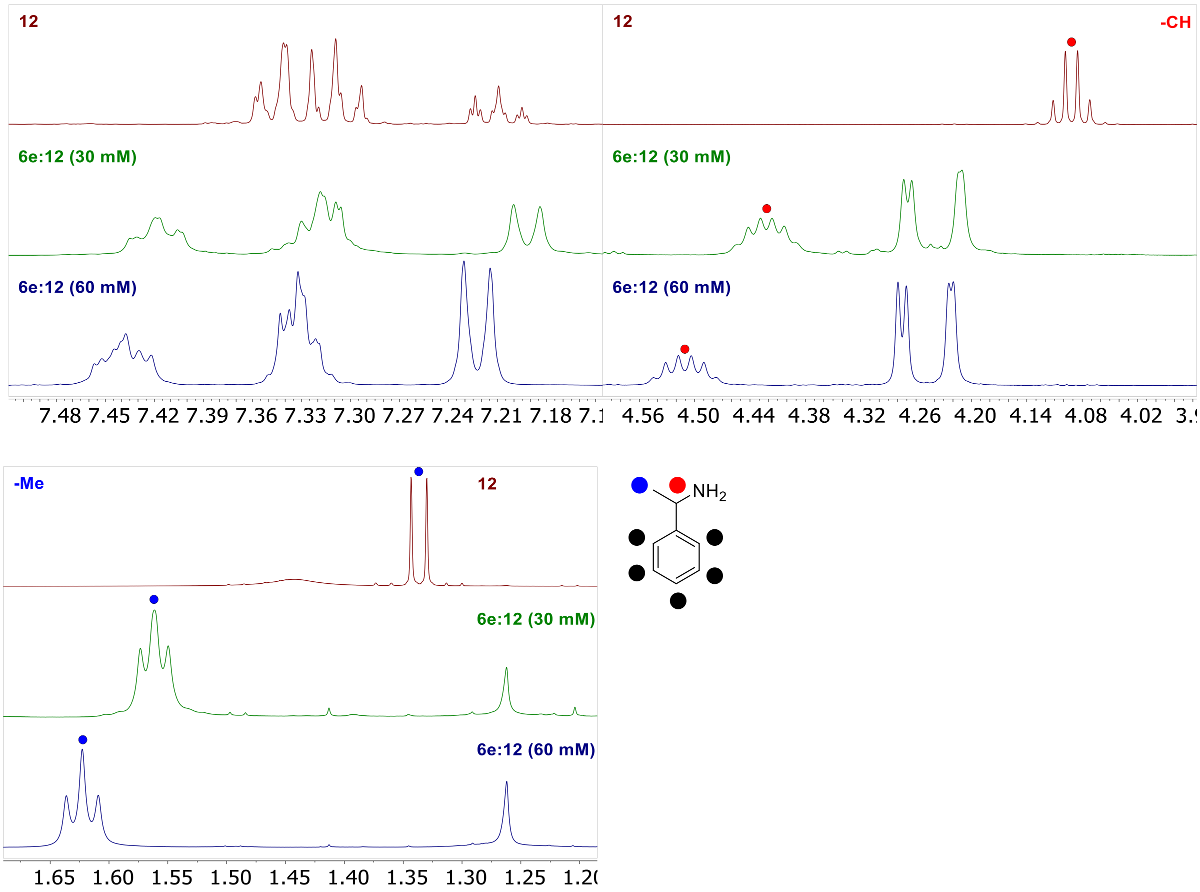

Figure S80. ^{1}H NMR (500 MHz, Methylene Chloride-*d*$_2$, 21 °C) spectra of: pure *rac*-**12** (30 mM) (red line); *rac*-**12** (30 mM) in the presence of 1 equivalent of CSA **6e**; *rac*-**12** (30 mM) in the presence of 2 equivalents of CSA **6e**. Only selected spectral regions containing the signals of **12** are reported.

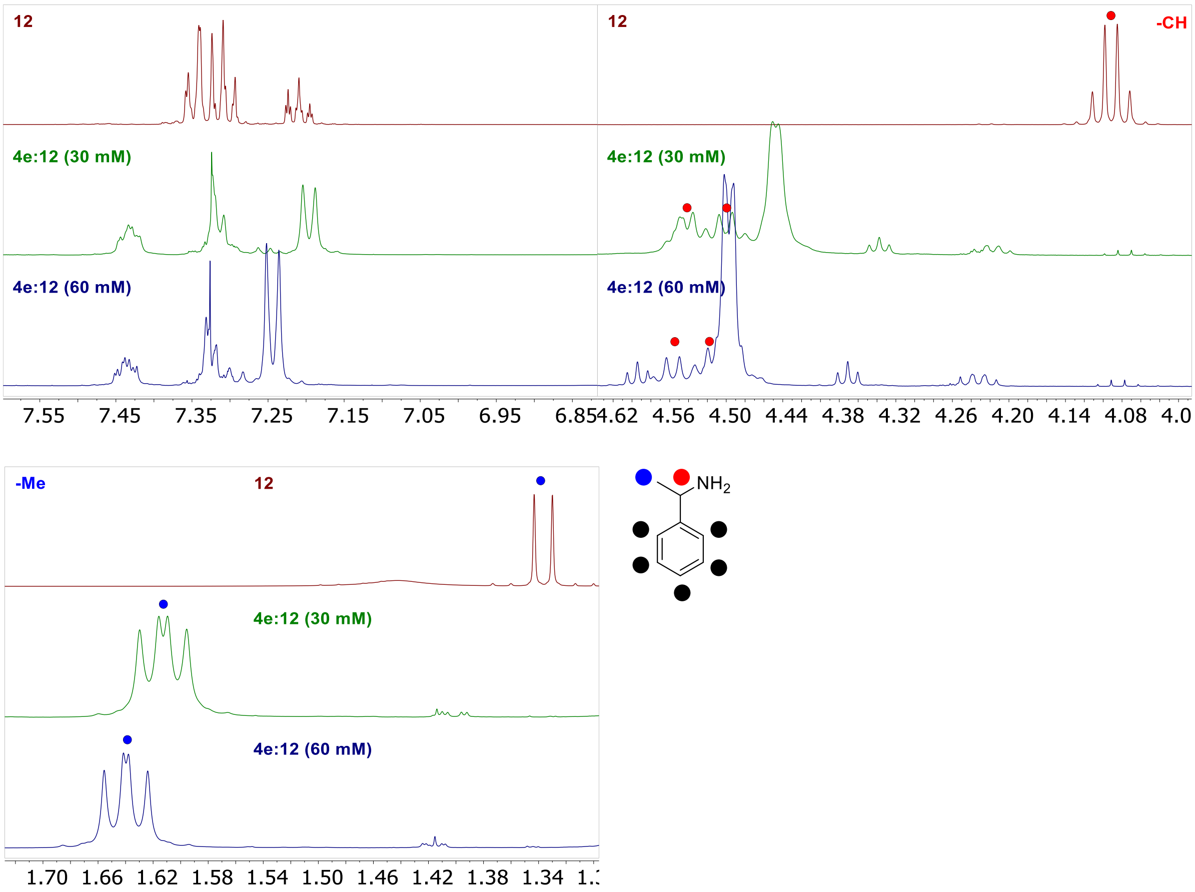

Figure S81. ^{1}H NMR (500 MHz, Methylene Chloride-*d*$_2$, 21 °C) spectra of: pure *rac*-**12** (30 mM) (red line); *rac*-**12** (30 mM) in the presence of 1 equivalent of CSA **4e**; *rac*-**12** (30 mM) in the presence of 2 equivalents of CSA **4e.** Only selected spectral regions containing the signals of **12** are reported.

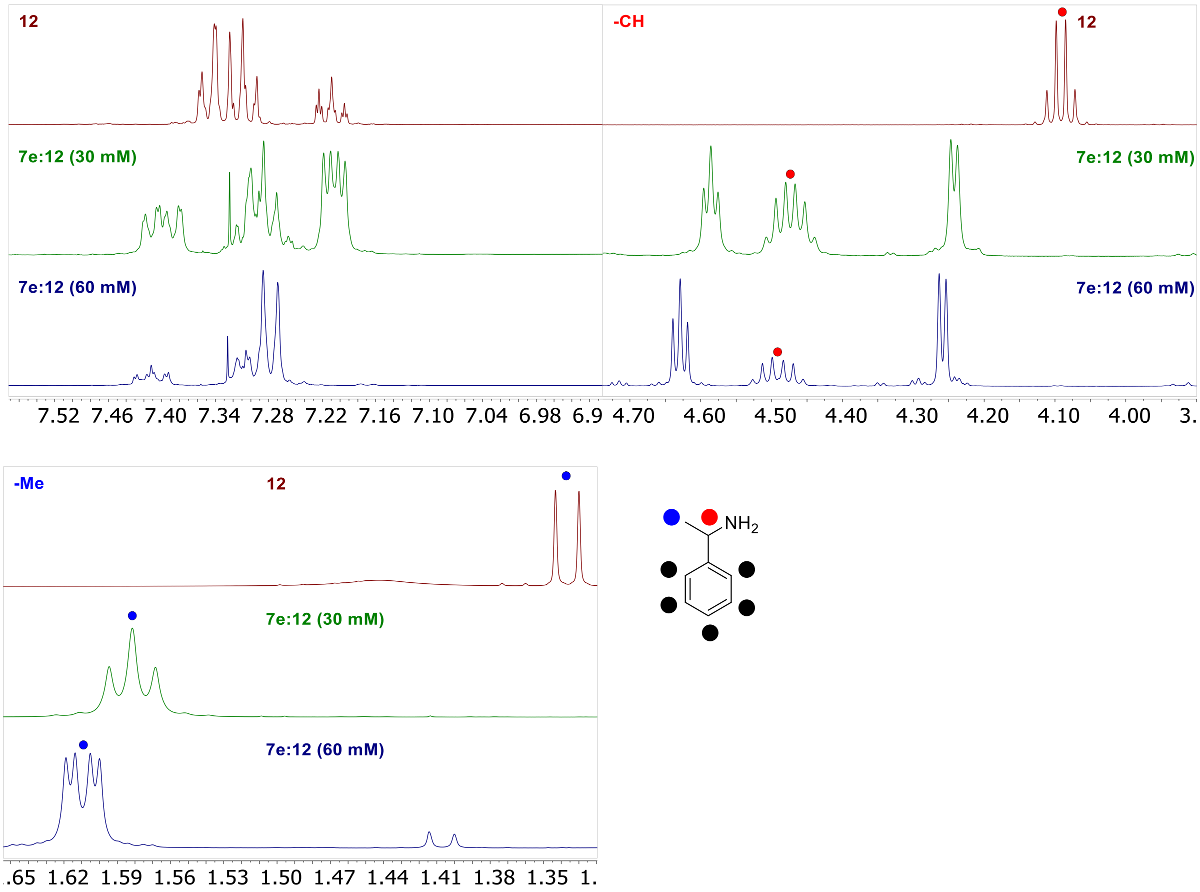

Figure S82. ^{1}H NMR (500 MHz, Methylene Chloride-d_2, 21 °C) spectra of: pure *rac*-**12** (30 mM) (red line); *rac*-**12** (30 mM) in the presence of 1 equivalent of CSA **4e**; *rac*-**12** (30 mM) in the presence of 2 equivalents of CSA **4e**. Only selected spectral regions containing the signals of **12** are reported.

Optimization of enantiodiscrimination conditions of compound ***9*** *employing CSA* ***7c***

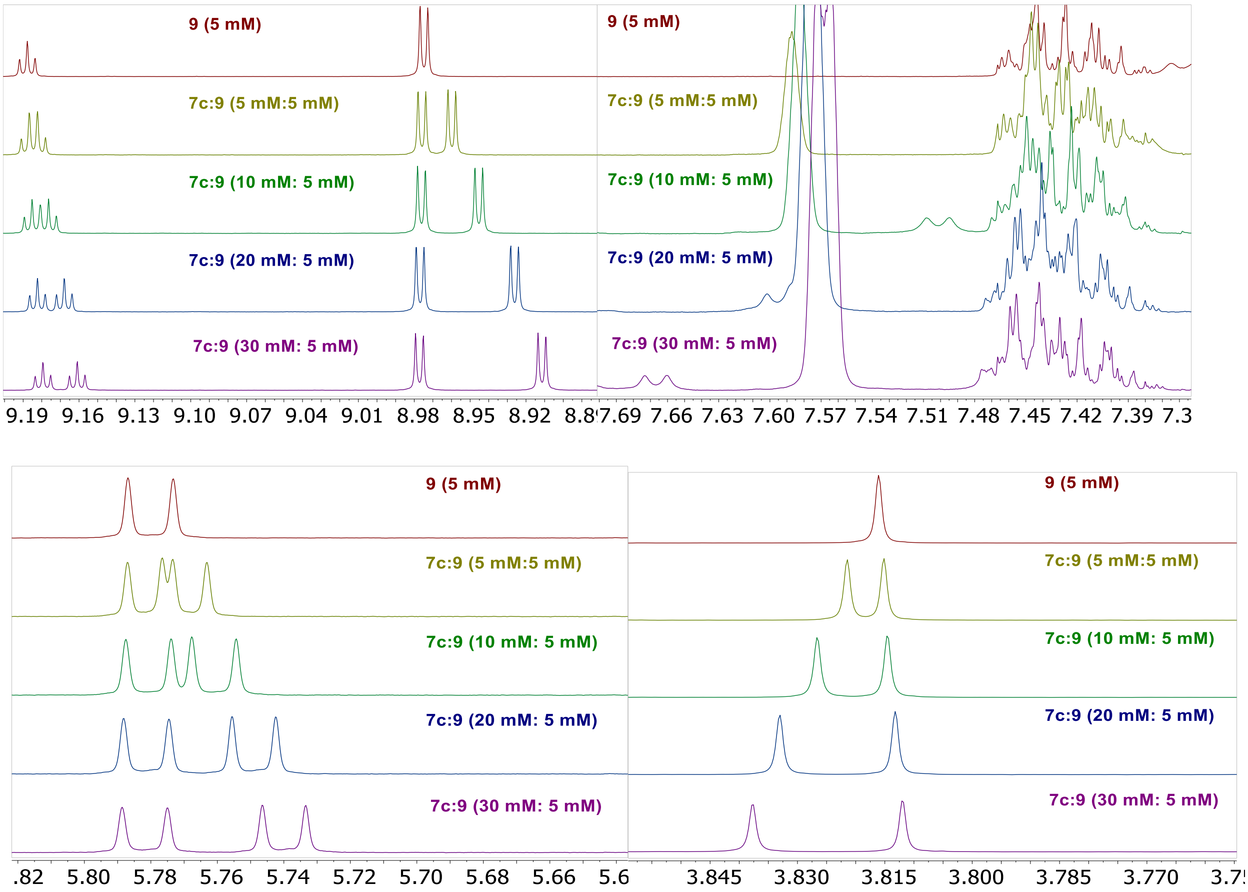

Figure S83. ^{1}H NMR (500 MHz, Chloroform-*d*, 21 °C) spectra of *rac*-**9** (5 mM) in the presence of 1, 2, 4 or 6 equivalents of CSA **7c**. Only selected spectral regions containing the signals of **9** are reported.

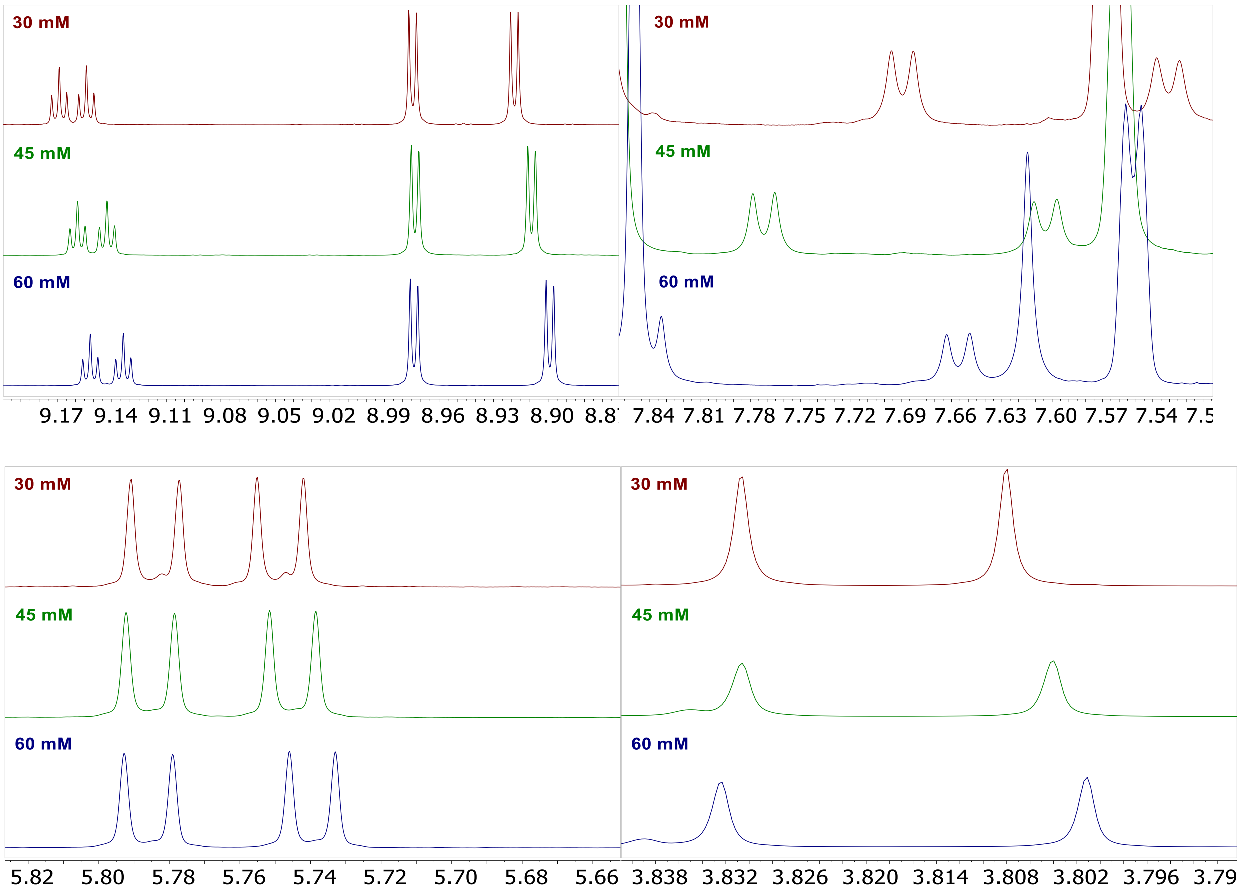

Figure S84. ^{1}H NMR (500 MHz, Chloroform-*d*, 21 °C) spectra of *rac*-**9** in the presence of 1 equivalent of CSA **7c**. Red line: *rac*-**9** (30 mM). Green line: *rac*-**9** (45 mM). Blue line: *rac*-**9** (60 mM). Only selected spectral regions containing the signals of **9** are reported.

NMR characterization of CSA ***7c****.*

On the basis of ^{1}H-^{13}C correlations detected in the HSQC map (**Figure S85**, *Supporting Information*), the signals between 3.8 ppm and 4.2 ppm were assigned to the two pairs of diastereotopic methylene protons (H_3/H_{3a} and H_6/H_{6a}), whereas sharp signals between 4.6 ppm and 5.4 ppm were assigned to methine protons (H_1, H_2, H_4 and H_5).

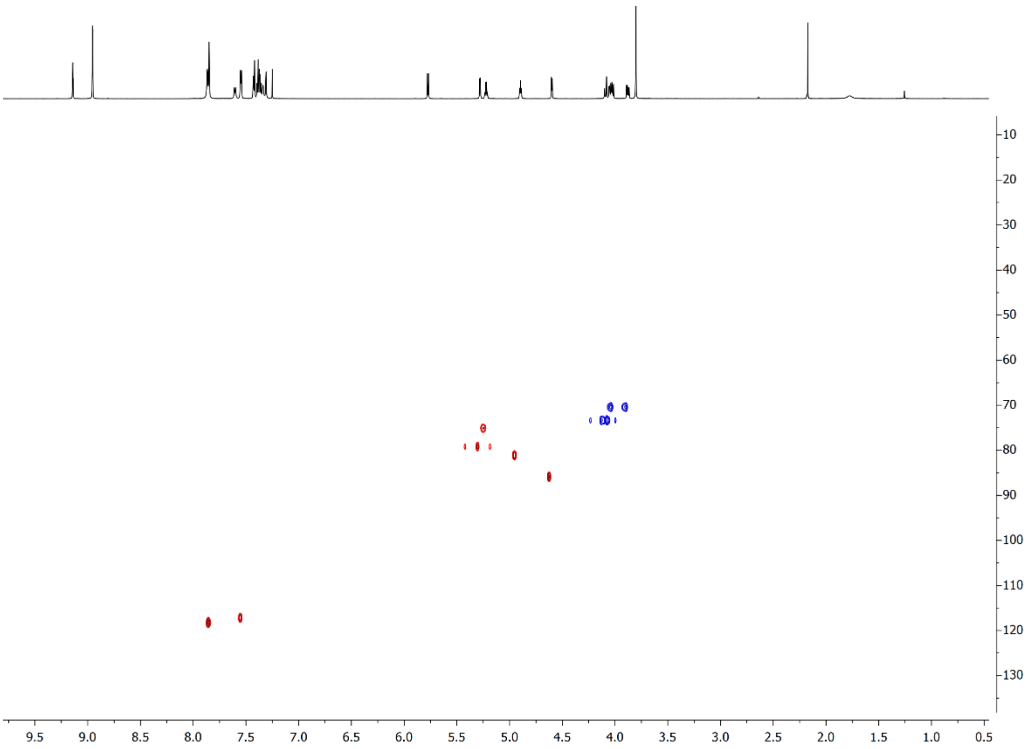

Figure S85. ^{1}H-^{13}C HSQC (600 MHz, $CDCl_3$, 45 mM, 25 °C) map of **7c**: red correlations for methine and methyl carbons; blue correlations for methylene carbons.

The methine protons (H_1, H_2, H_4 and H_5) were attributed taking into account the scalar and dipolar correlations detected in the COSY and ROESY maps, respectively (**Figure S86**, *Supporting Information*).

In particular, the two methine resonances at 4.63 ppm and 4.95 ppm were attributed to the protons at the junction of the pentaatomic rings (H_1/H_4), as they showed reciprocal scalar

correlations and no J-coupling correlations with methylene protons (**Figure S86a**, *Supporting Information*). For proton resonating at 4.95 ppm another scalar correlation was detected at the frequency of 5.25 ppm, due to a methine proton. Among them, resonance centered at 4.95 ppm was attributed to H_1 and that one at 4.63 ppm to H_4, since only H_1 can lead to another scalar correlation with proton H_2 while no J-coupling between H_4 and H_5 could be observed due to a dihedral angle close to 90°. H_1 and H_4 multiplicities are in accordance to above-said attribution, since H_1 is a triplet due to J-couplings with H_4 and H_2 ($^3J_{1-2} = {}^3J_{1-4} = 5.0$ Hz), whereas H_4 is a doublet due to the coupling with H_1. On the basis of these considerations, following the scalar coupling showed in the COSY map, H_2 and H_5 could be assigned (5.25 ppm and 5.30 ppm, respectively) (**Figure S86a**, *Supporting Information*). Methylene couples identified by HSQC map (**Figure S85,** *Supporting Information*) were distinguished still on the basis of their scalar coupling: the protons resonating at 4.05 ppm and 3.90 ppm were attributed to H_3/H_{3a} due to a scalar coupling with H_2 and protons resonating at 4.07 ppm and 4.12 ppm to H_6/H_{6a} due to a J-coupling with H_5.

On the basis of the relative intensities of ROEs originated by H_2 and H_5 (**Figures S86b**, *Supporting Information* and **S87c-d**) at the frequencies of the methylene protons, the methylene protons cisoid (H_3/H_6) and transoid (H_{3a}/H_{6a}) to them were distinguished, with H_3 and H_{3a} respectively at 4.05 ppm and 3.90 ppm, and H_6 and H_{6a} at 4.07 ppm and 4.12 ppm, respectively.

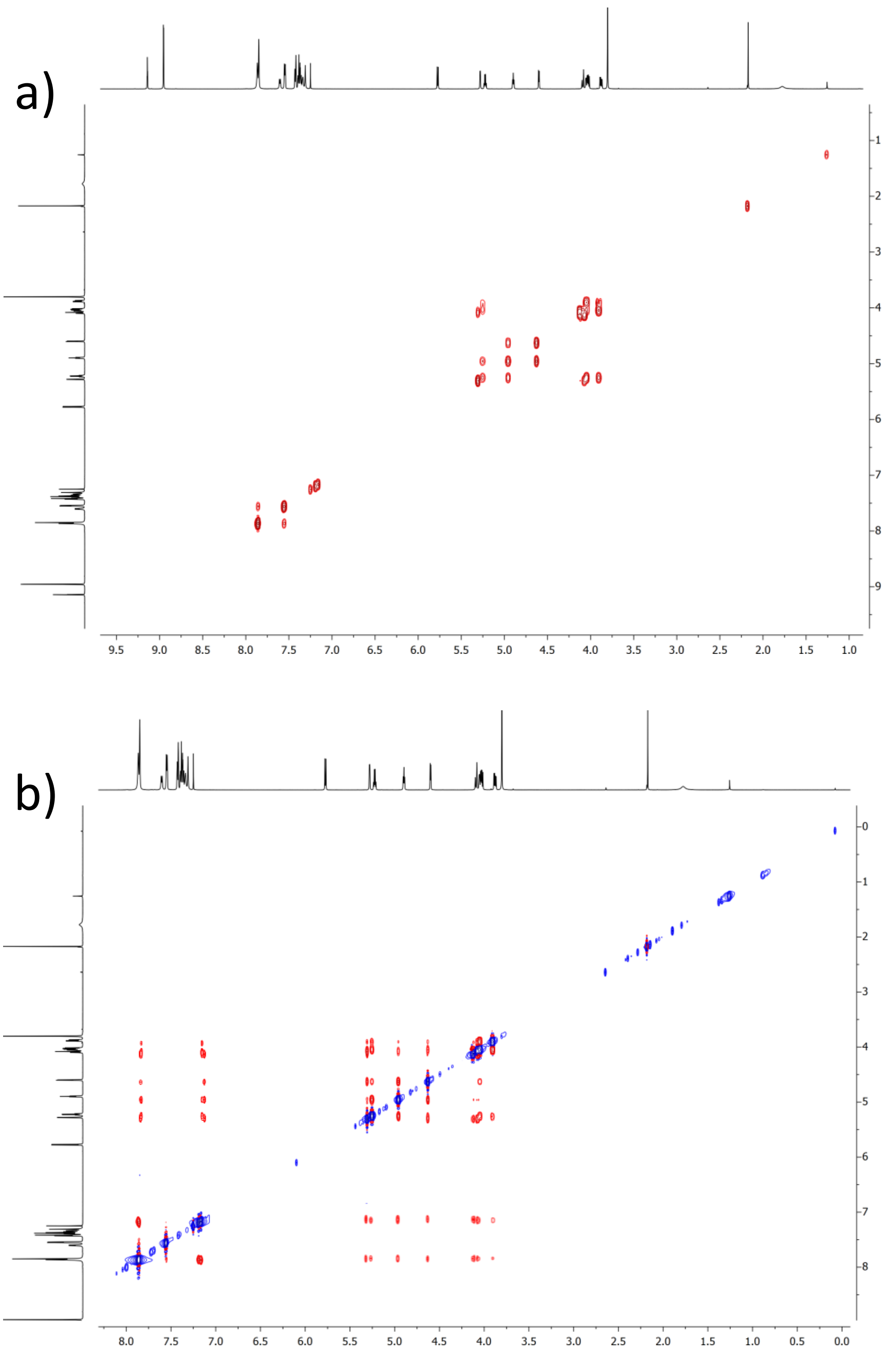

Figure S86. a) COSY (600 MHz, $CDCl_3$, 45 mM, 25 °C) map of **7c**. b) ROESY (600 MHz, $CDCl_3$, 45 mM, 25 °C, mix=0.5 s) map of **7c**.

These attributions were even confirmed by other dipole-dipole interactions (**Figure S86b**, *Supporting Information*, **Figures S87a** and S**87b**). In particular, ROE effects observed for the resonance centered at 4.63 ppm were in completely accordance with its attribution to H_4, since H_4 is the only one which can originate through space dipole-dipole interactions with its vicinal proton H_5 (5.30 ppm) and with proton H_3 (4.05 ppm) lying on the same side of the CSA structure (**Figure S86b**, *Supporting Information* and **Figure** S**87b**). Proton H_1, instead, showed dipolar interactions due to spatial proximity only with H_4 and its vicinal proton H_2 (5.25 ppm) (**Figure S86b**, *Supporting Information*, and **Figure** S**87a**).

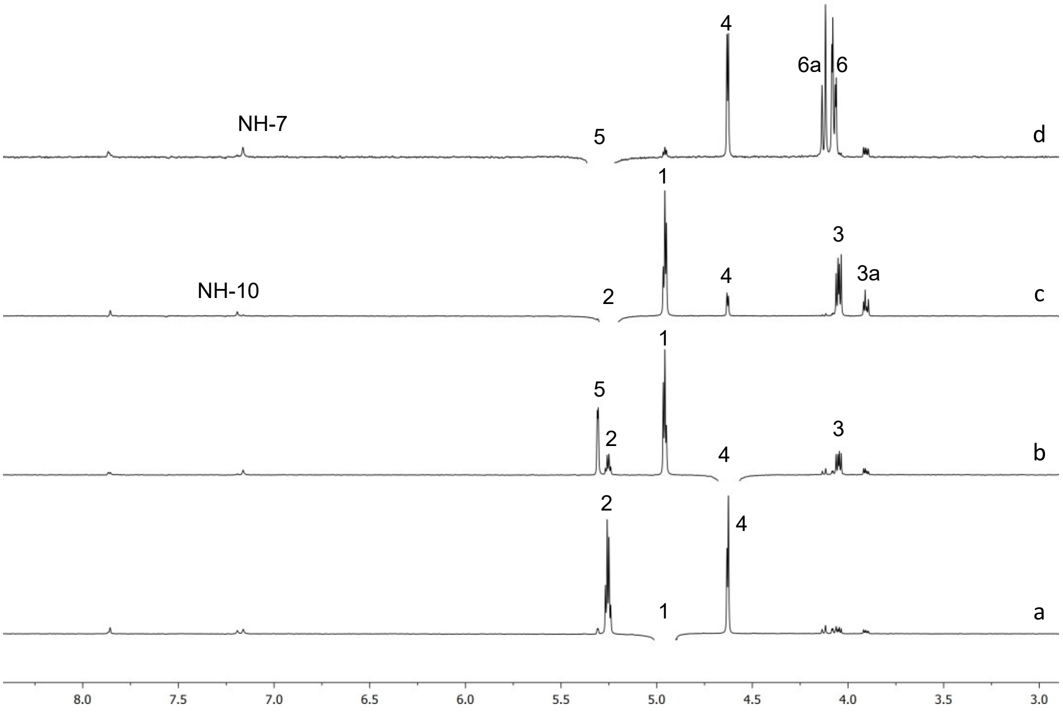

Figure S87. 1D ROESY (600 MHz, $CDCl_3$, 45 mM, 25 °C, mix=0.5 s) spectra of **7c**, corresponding to the perturbation of: a) H_1; b) H_4; c) H_2; d) H_5.

Moving to the high-frequencies spectral region, the two signals at 7.18 ppm and 7.22 ppm, which were not detected in the ^1H-^{13}C HSQC map (**Figure S85**, *Supporting Information*), were attributed to the two NH moieties. In particular, on the basis of reciprocal dipolar correlation

H_5-NH-7, the signal at 7.18 ppm was attributed to NH-7 (**Figure S87d**), while the signal at 7.22 ppm was assigned to NH-10, being detected the H_2-NH-10 dipolar correlation (**Figure S87c**). Finally, ROE effects given by NH-7 and NH-10 allowed us to distinguish partially superimposed *ortho* signals of the 3,5-bis(trifluoromethyl)phenyl moieties, respectively at 7.87 ppm for H_8 and 7.86 ppm for H_{11} (**Figure S86b**, *Supporting Information*). *Para*-protons of the two aromatic rings were assigned at 7.56 ppm and 7.55 ppm, but not unequivocally assigned to H_9/H_{12}. Characterization data are collected in **Table S1**, *Supporting Information*.

Table S1. ^{1}H NMR (600 MHz, $CDCl_3$, 45 mM, 25 °C) characterization of **7c**

**Proton**	**δ (ppm)**	**multiplicity**[a]	**J (Hz)**
1	4.95	t	J_{1-2}: 5.0; J_{1-4}: 5.0
2	5.25	ddd	J_{2-3}: 6.0; J_{2-3a}: 5.3; J_{2-1}: 5.0
3	4.05	dd	J_{3-3a}: 10.0; J_{3-2}: 6.0
3a	3.90	dd	J_{3a-3}: 10.0; J_{3a-2}: 5.3
4	4.63	d	J_{4-1}: 5.0
5	5.30	d	J_{5-6}: 3.4
6	4.07	dd	J_{6-6a}: 10.7; J_{6-5}: 3.4
6a	4.12	d	J_{6a-6}: 10.7
NH-7	7.18	s	
8	7.87	s	
9/12	7.55, 7.56	s	
NH-10	7.22	s	
11	7.86	s	

[a]t=triplet, ddd=double double doublet, dd=double doublet, d=doublet, s=singlet

Determination of association constants of the diastereomeric complexes

Association constants of the two diastereomeric complexes were obtained by analyzing the chemical shifts dependence of H_{ortho} protons of the 3,5-dinitrophenyl moiety in compound **9** and H_1 proton of CSA **7c**. ^1H NMR spectra were recorded for a set of solutions in $CDCl_3$ with variable concentration (total concentration between 120 mM and 1.2 mM) of equimolar mixture CSA/(S)-**9** and CSA/(R)-**9**. The non-linear fitting of chemical shifts *versus* concentration data was performed by using **equation S1**

$$C_0 = \frac{1}{K}\frac{(\delta_{obs}-\delta_f)(\delta_b-\delta_f)}{(\delta_b-\delta_{obs})^2} \quad \textbf{(equation S1)}$$

where K is the association constant, C_0 is the concentration of each component in the equimolar mixture, δ_{obs}, δ_f, and δ_b are the observed chemical shift and the chemical shift for the free and bound state, respectively

*Determination of enantiomeric ratio in scalemic mixtures of **9** by chiral chromatography (HPLC) and ^{1}H NMR employing **7c** as CSA.*

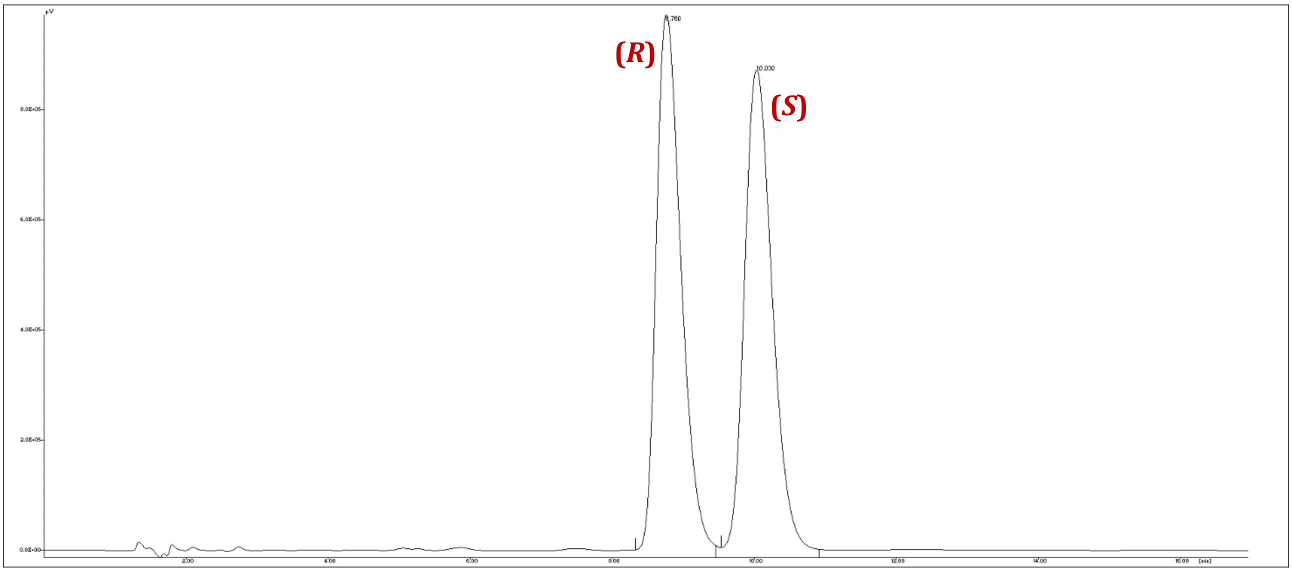

Compound	RT [min]	Area [µV·sec]	Area %
(*R*)-**9**	8.760	22928836.400	49.963
(*S*)-**9**	10.030	22962726.200	50.037

Figure S88. HPLC chromatogram of racemic N-3,5-dinitrobenzoylphenylglycine methyl ester (**9**). (Lux 5 µm Cellulose-1, 250 x 460 mm, Hexane:Ethanol 80:20, 2 mL/min, 25 °C).

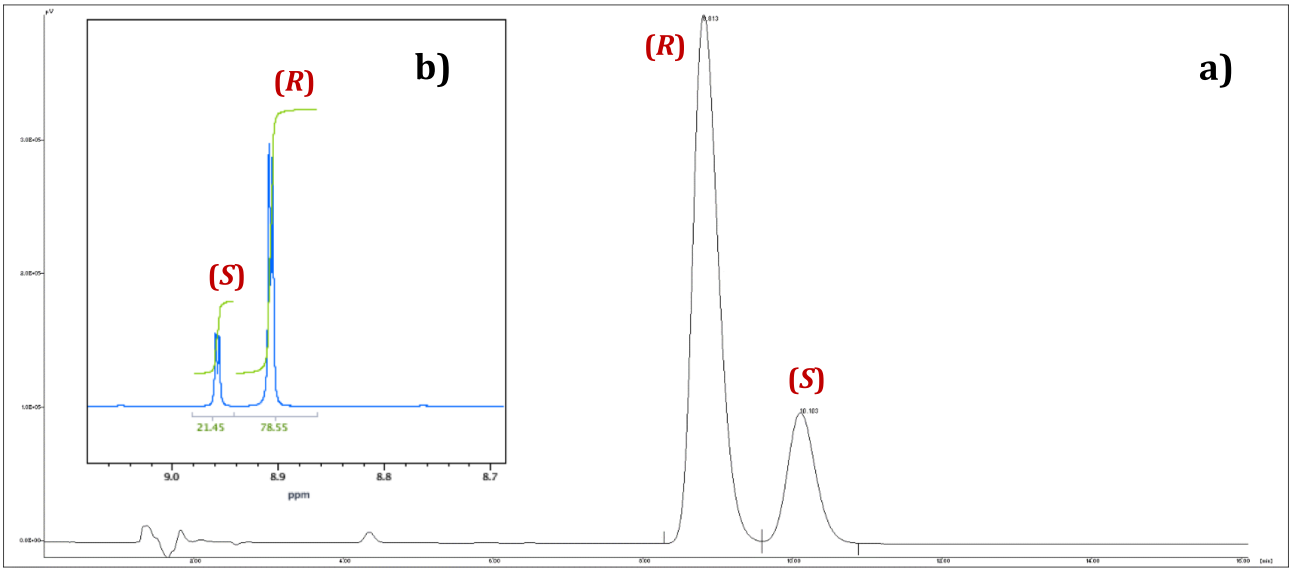

	HPLC			NMR
**Compound**	**RT [min]**	**Area [µV·sec]**	**Area %**	**Area %**
(*R*)-**9**	8.813	9056478.800	79.072	78.55
(*S*)-**9**	10.103	2397031.000	20.928	21.45

Figure S89. Determination of the enantiomeric ratio of a scalemic mixture of compound **9** (nominal enantiomeric ratio (*R*)-**9**/(*S*)-**9** 79:21): a) HPLC chromatogram (Lux 5 µm Cellulose-1, 250 x 460 mm, Hexane:Ethanol 80:20, 2 mL/min, 25 °C), b) ^{1}H NMR spectra in the presence of 1 equivalent of compound **7c** as CSA (600 MHz, Chloroform-*d*, 45 mM).

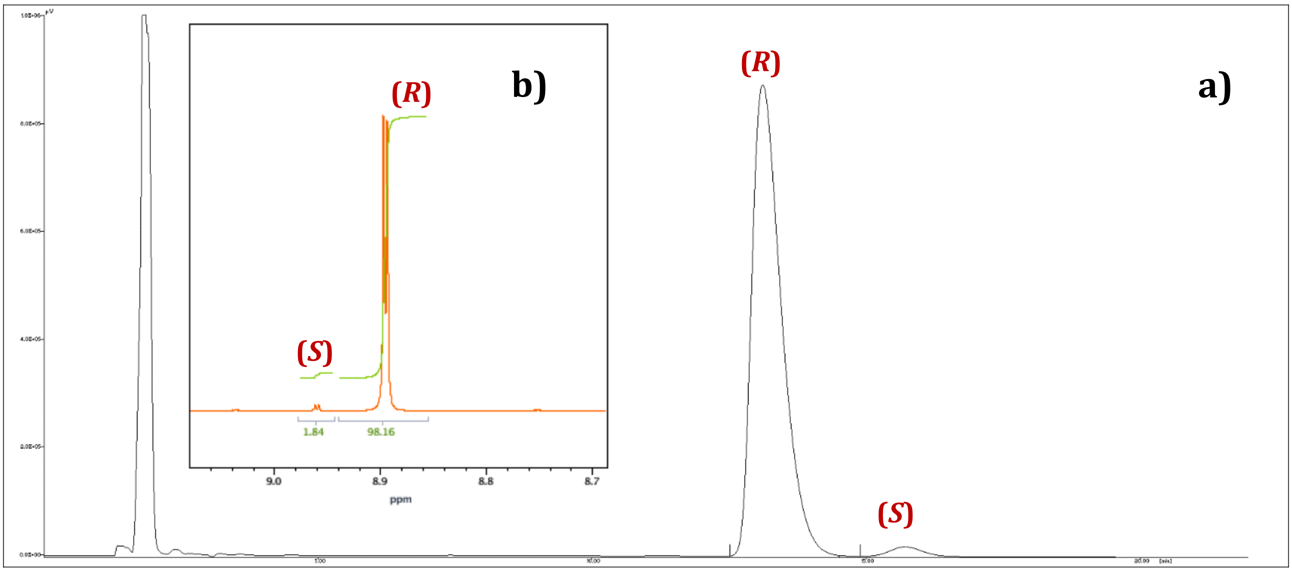

	HPLC			NMR
**Compound**	**RT [min]**	**Area [µV·sec]**	**Area %**	**Area %**
(*R*)-**9**	13.107	31942349.800	98.530	98.16
(*S*)-**9**	15.700	476613.600	1.470	1.84

Figure S90. Determination of the enantiomeric ratio of a scalemic mixture of compound **9** (nominal enantiomeric ratio (*R*)-**9**/(*S*)-**9** 98.5:1.5): a) HPLC chromatogram (Lux 5 µm Cellulose-1, 250 x 460 mm, Hexane:Ethanol 80:20, 1.5 mL/min, 25 °C), b) ^{1}H NMR spectra in the presence of 1 equivalent of compound **7c** as CSA (600 MHz, Chloroform-*d*, 45 mM).

References

(1) Santos Ribeiro, A.; Kanazawa, A.; Navarro, D. M. A. F.; Moutet, J. C.; Navarro, M. Synthesis of (*R*)-(−) and (*S*)-(+)-3-(1-pyrrolyl)propyl-(3,5-dinitrobenzoyl)-_-phenylglycinate and derivatives. A suitable chiral polymeric phase precursor *Tetrahedron: Asymmetry* **1999**, *10*, 3735–3745.

(2) Terada, M.; Tanaka, H.; Sorimachi, K. Enantioselective Direct Aldol-Type Reaction of Azlactone via Protonation of Vinyl Ethers by a Chiral Brønsted Acid Catalyst. *J. Am. Chem. Soc.* **2009**, *131* (10), 3430–3431.

(3) Lu, G.; Birman, V. B. Dynamic Kinetic Resolution of Azlactones Catalyzed by Chiral Brønsted Acids. *Org. Lett.* **2011**, *13* (7), 1896–1896.
